# Water Reduction and Dihydrogen Addition in Aqueous Conditions With *ansa‐*Phosphinoborane

**DOI:** 10.1002/chem.202201927

**Published:** 2022-08-31

**Authors:** Kristina Sorochkina, Konstantin Chernichenko, Vladimir V. Zhivonitko, Martin Nieger, Timo Repo

**Affiliations:** ^1^ Department of Chemistry University of Helsinki A. I. Virtasen aukio 1 00014 Helsinki Finland; ^2^ Chemical Process Research and Development Janssen Pharmaceutica Turnhoutseweg 30 2340 Beerse Belgium; ^3^ NMR Research Unit University of Oulu P.O. Box 3000 90014 Oulu Finland

**Keywords:** DFT, frustrated Lewis pairs, hydrogen activation, mechanism, umpolung of proton, water reduction

## Abstract

*Ortho*‐phenylene‐bridged phosphinoborane (2,6‐Cl_2_Ph)_2_B‐C_6_H_4_‐PCy_2_ **1** was synthesized in three steps from commercially available starting materials. **1** reacts with H_2_ or H_2_O under mild conditions to form corresponding zwitterionic phosphonium borates **1‐H_2_** or **1‐H_2_O**. NMR studies revealed both reactions to be remarkably reversible. Thus, when exposed to H_2_, **1‐H_2_O** partially converts to **1‐H_2_** even in the presence of multiple equivalents of water in the solution. The addition of parahydrogen to **1** leads to nuclear spin hyperpolarization both in dry and hydrous solvents, confirming the dissociation of **1‐H_2_O** to free **1**. These observations were supported by computational studies indicating that the formation of **1‐H_2_** and **1‐H_2_O** from **1** are thermodynamically favored. Unexpectedly, **1‐H_2_O** can release molecular hydrogen to form phosphine oxide **1‐O**. Kinetic, mechanistic, and computational (DFT) studies were used to elucidate the unique “umpolung” water reduction mechanism.

## Graphical abstract

Water reduction and H_2_ activation by an *ortho*‐phenylene‐bridged phosphinoborane compound are demonstrated. The mechanisms of these transformations are investigated using kinetic measurements and DFT calculations. Nuclear spin hyperpolarization is detected as a result of parahydrogen (para‐H_2_) activation by the phosphinoborane in the presence of water, proving the reversibility of water addition to this compound.

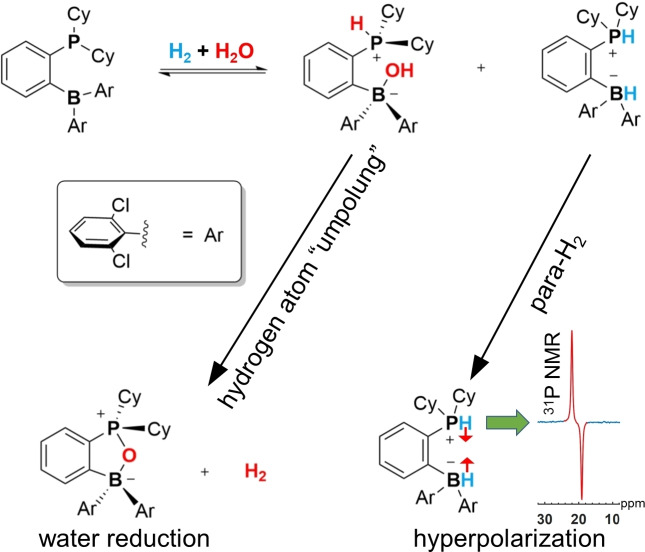

## Introduction

A cooperative reactivity of frustrated Lewis pairs (FLPs) opens up outstanding potential for small molecules activation. FLPs can split C−O, S−O, N−O, N−N, and C−H bonds,^[1]^ but most notable is the activation and the catalytic transfer of molecular hydrogen to various organic substrates.^[2]^ The FLP reactive centers can be represented by various atoms, including metals,^[3]^ however, the majority of reported FLPs are based on highly Lewis acidic (LA) boranes and bulky phosphines or amines as bases (LB). Intrinsically high oxophilicity of boron leads to functional groups sensitivity, particularly, to OH‐containing molecules especially water,[^4^, ^12^] limiting the utility of FLPs as catalysts.

As part of our general interest in extending the boundaries of the FLP chemistry, we have been focused on preparing FLPs that can tolerate water. Several successful attempts to develop FLP hydrogenation catalysts that could tolerate moisture were reported.[^5^, ^6^] Our interest, however, lies in the preparation of stable, i. e., directly detectable, hydrogen adducts existing in the presence of over‐stoichiometric amounts of water, ideally in aqueous solutions. Apart from purely fundamental significance, such FLPs might find practical applications as parahydrogen‐induced polarization (PHIP) tags in biologically relevant media. Parahydrogen is an accessible source for creating nuclear spin hyperpolarization and we have shown previously that adducts of intramolecular FLPs and parahydrogen produce PHIP with orders of magnitude NMR signal enhancement.^[7]^

Our previous works demonstrated that intramolecular FLPs bridged by *o*‐phenylene (*ansa*‐scaffold) exhibited enhanced reactivity in comparison to intermolecular FLPs.^[8]^ In addition, fixed proximity of LA and LB sites in the *ansa*‐FLPs offers flexible possibilities for their modification: *ansa*‐FLP that was built even with the smallest LA group, ‐BH_2_, exhibited hydrogen splitting reactivity.^[9]^ Herein we report the design of a highly sterically hindered activation pocket that can disfavor water binding by steric exclusion.

**Figure 1:**
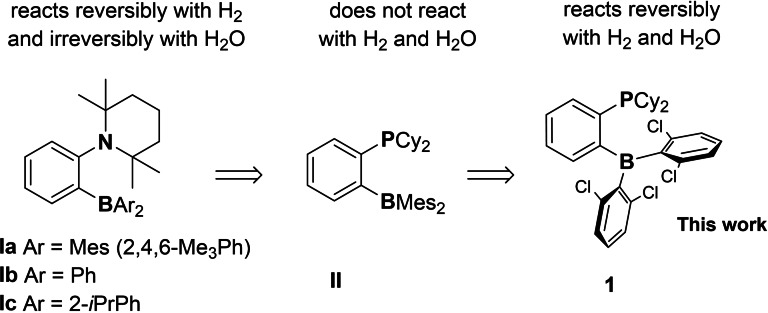
Efforts in designing water tolerant *ansa*‐FLPs.

Previously we attempted preparation of water tolerant FLPs but they either strongly favored the formation of water adducts (Figure 1, **Ia**–**c**)[^7b^, ^7c^] or were reactive to neither water nor hydrogen (Figure 1, **II**).^[10]^ In continuation of these efforts we designed new *ansa*‐phosphinoborane 2‐[(2,6‐Cl_2_Ph)_2_B]‐C_6_H_4_‐PCy_2_ **1**. Replacement of the CH_3_ groups with Cl was expected to enhance the acidity of the boryl site while retaining necessary steric effect. This opens unique capability for the addition of H_2_ and H_2_O, both in a reversible manner, and rather unexpected reduction of H_2_O to H_2_.

## Results and Discussion

### Synthesis of 1, splitting of H_2_ and H_2_O

**Scheme 1:**
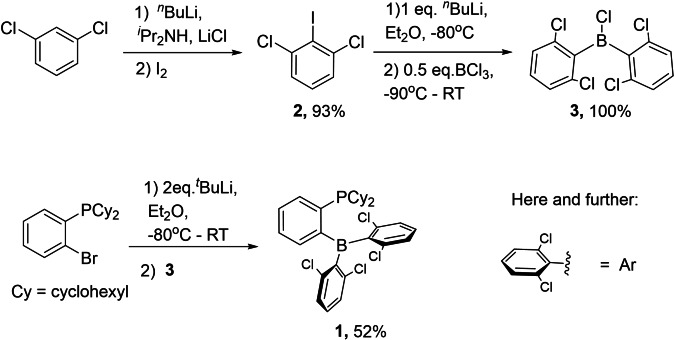
Three‐step synthesis of *ansa*‐phosphinoborane **1**.

**Figure 2:**
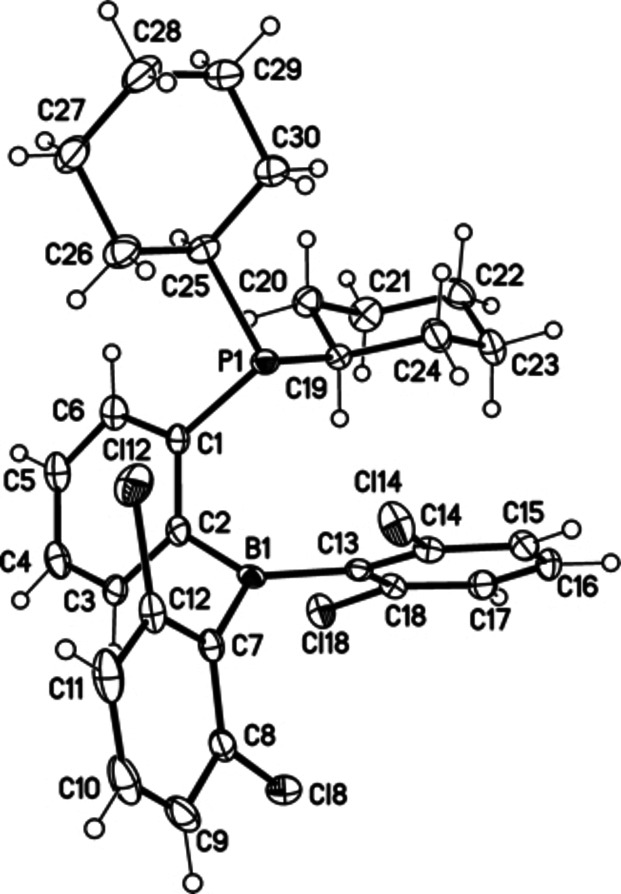
Crystal structure of *ansa*‐phosphinoborane **1** reveals P^…^B separation 3.170(4) Å (displacement parameters are drawn at 50 % probability level).

We developed a three‐step synthesis of *ansa*‐phosphinoborane **1** from commercially available 1,3‐dichlorobenzene, 2‐bromophenyl(dicyclohexyl)phosphine, and BCl_3_ (Scheme 1). Recrystallization of **1** from a hexane‐toluene mixture at −20 °C gave the pure product as yellow crystals with 48 % overall yield. ^11^B and ^31^P NMR displayed singlet signals at 67.03 and 0.51 ppm, respectively, revealing no dative P−B coordination in solution.^[11]^ The structure determined from the X‐ray diffraction analysis featured P⋅⋅⋅B separation 3.170(4) Å, thus excluding a dative P−B bond in solid **1** (Figure 2).

**Scheme 2:**
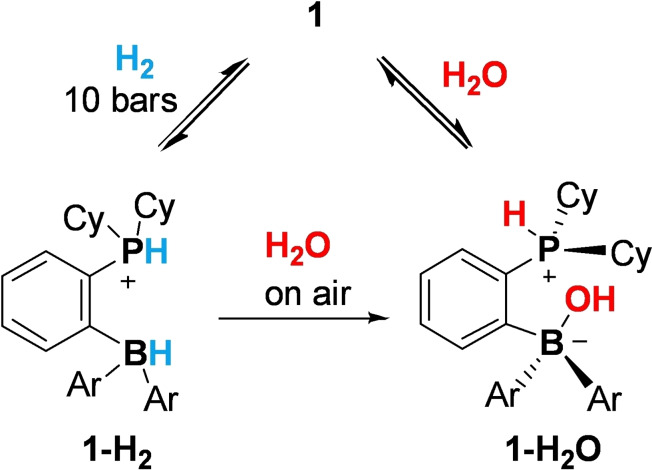
Reactivity of the *ansa*‐phosphinoborane **1** towards H_2_O and H_2_.

Having **1** in hands, we explored its reactivity towards H_2_ and H_2_O (Scheme 2). Exposing solutions of **1** in CD_2_Cl_2_ or C_6_D_6_ to 10 bars of H_2_ at room temperature led to the formation of zwitterionic adduct **1‐H_2_** identified by the characteristic P−H and B−H ^1^H NMR signals (see Table 1).^[12]^ Exposing the solution of **1‐H_2_** in C_6_D_6_ to D_2_ led to H/D exchange and isotopic scrambling, revealing that hydrogen activation is reversible (75 % conversion of the H_2_/D_2_ mixture to HD after being kept at room temperature for 12 h under 5 bar pressure, see Supporting Information for details).

**Table 1:** ^1^H, ^31^P and ^11^B NMR spectral data for **1**, adducts **1‐H_2_** and **1‐H_2_O**, and **1‐O** in CD_2_Cl_2_ (brm – broad multiplet, s – singlet, brs – broad singlet, dt – doublet of triplets, m ‐ multiplets).

	δ(^1^H NMR)/ppm			δ(^31^P NMR)/ppm	δ(^11^B NMR)/ppm
	P‐H	B(O)‐H	PC_Cy_‐H		
**1**	–	–	1.79 (brm)	0.51 (s)	67.03 (brs)
**1‐H_2_**	6.22 (dt)	4.06 (q)	2.64 (brm)	19.59 (brs)	−11.79 (d)
**1‐H_2_O**	4.87 (dt)	3.55 (s)	2.56 (brm)	43.30 (d)	0.67 (s)
**1‐O**	–	–	2.20 (m)	83.67 (s)	7.46 (brs)

**Figure 3:**
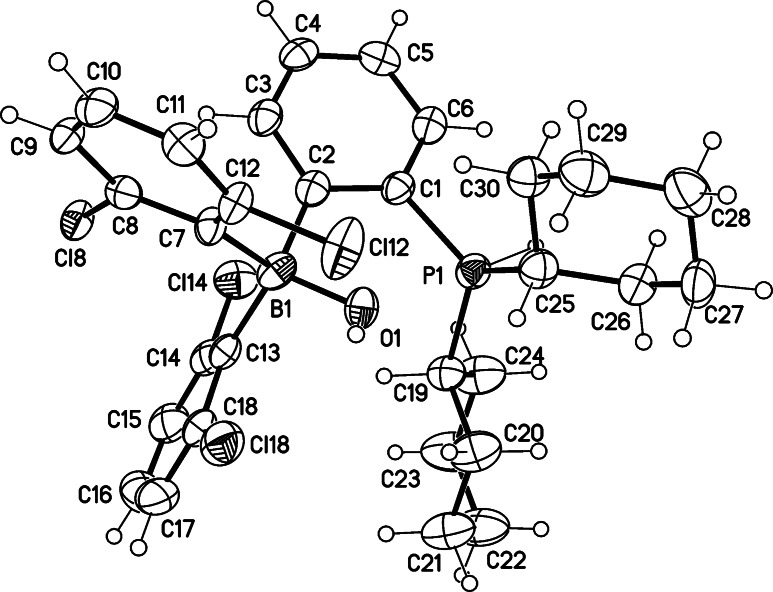
Crystal structure of phosphinoborane **1‐H_2_O** reveals *exo‐*configuration (displacement parameters are drawn at 50 % probability level). Selected distances (Å): B1‐O1 1.463(7), P1⋅⋅⋅O1 2.311(4), P1⋅⋅⋅B1 3.195(6).

Upon release of H_2_ pressure and exposure of the NMR sample to air, further monitoring by the NMR spectroscopy showed rapid conversion of **1‐H_2_** to water adduct **1‐H_2_O**. Alternatively, **1‐H_2_O** can be prepared directly from **1** and water. ^1^H NMR spectroscopy exhibited two distinct signals corresponding to the P−H and BO−H groups (See Table 1). Recrystallization of **1‐H_2_O** followed by X‐ray diffraction analysis revealed *exo*‐configuration of **1‐H_2_O** wherein the B‐OH group is directed to the phosphorus atom while the P−H proton is directed outside of the FLP pocket (Figure 3). This geometry agrees well with our computational studies (see below) and previous in silico studies of similar compounds derived from **II**.^[10]^ In contrast, previously reported HX (X=OH, OR, F, Cl) adducts of *ansa*‐FLPs adopted *endo*‐configuration featuring intramolecular hydrogen bonding, which was thought to substantially stabilize these species.^[13]^ Such *endo*‐adducts feature slightly longer B−O bonds (1.52–1.53 Å)[^13a^, ^13g^] as compared to the experimentally observed *exo*‐form of **1‐H_2_O** (1.463(7) Å).

**Figure 4:**
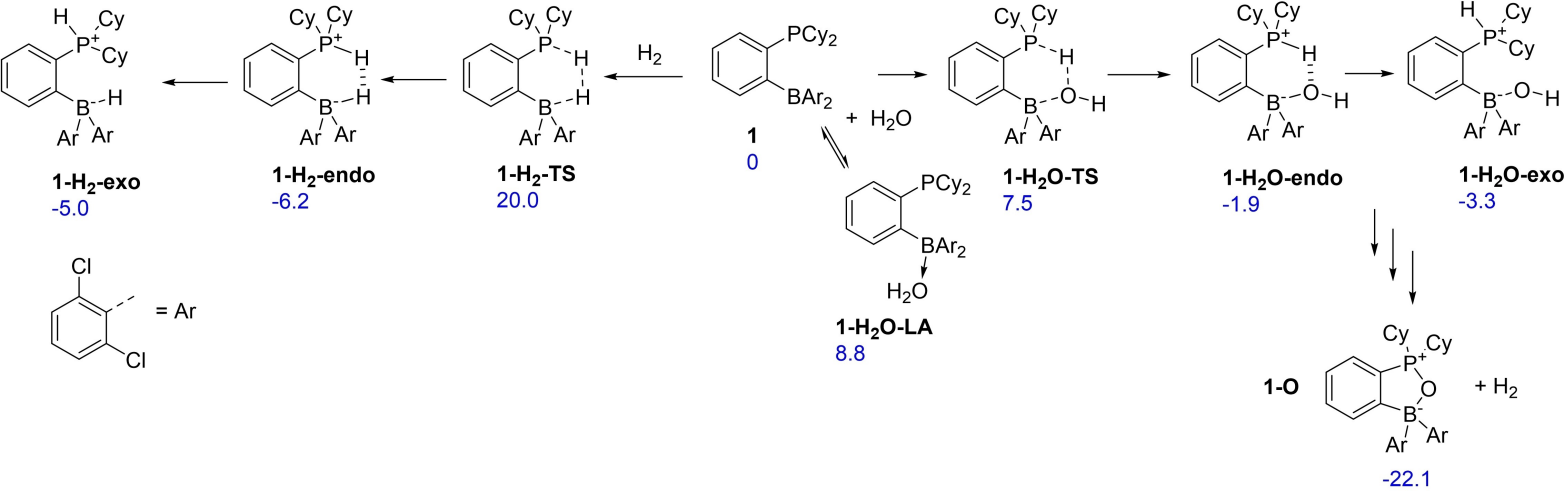
DFT studies of water and hydrogen addition to **1**. Solution phase Gibbs free energies computed at ωB97XD/6‐311++G(3df,3pd) level of theory are given in kcal/mol with respect to reactants in dichloromethane.

Quantum mechanical DFT calculations of water and hydrogen activation were in good agreement with experimental observations (Figure 4, see Supporting Information for details). Both processes were found to be thermodynamically favorable and kinetically feasible. Exo‐adduct **1‐H_2_O‐exo** was found to be by 1.4 kcal/mol more stable than **1‐H_2_O‐endo**, whereas for **1‐H_2_** the *endo*‐form was more stable.^[12]^ In line with the much higher propensity of polar O−H bonds for the heterolytic splitting, the addition of water to **1** is hindered by a very low, 7.5 kcal/mol, kinetic barrier whereas the hydrogen addition transition state **1‐H_2_‐TS** lies much higher at 20.0 kcal/mol. Interestingly, computations predict hydrogen addition to be more preferred over the addition of water by almost 3 kcal/mol in DCM. It was also possible to computationally identify unstable classical Lewis adduct **1‐H_2_O‐LA**.

### Water reduction

**Figure 5:**
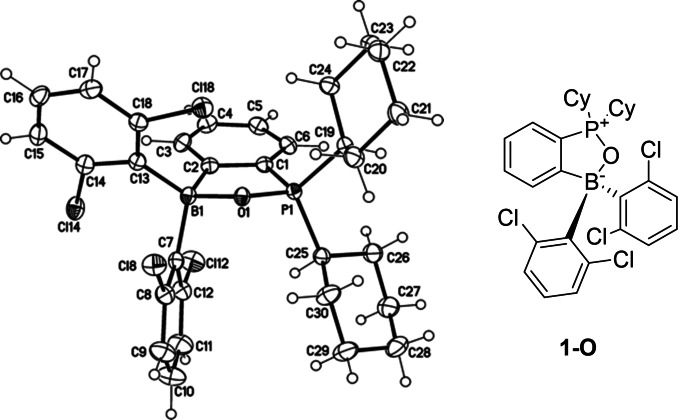
Crystal structure of phosphinoborane oxide **1‐O** (displacement parameters are drawn at 50 % probability level). Selected distances (Å): B1‐O1 1.569(0), P1‐O1 1.550(1), P1⋅B1 2.6074(16).

When **1‐H_2_O** or **1** with various amounts of water in the 1 : 1 CD_2_Cl_2_:CD_3_CN solution were monitored by the NMR spectroscopy at 25 °C or elevated temperatures, new signals were observed in ^1^H NMR spectra along with additional singlets at 83.67 ppm in the ^31^P and at 7.46 ppm in the ^11^B NMR spectra. The new species was isolated and, based on the results of the XRD analysis, identified as phosphinoborane oxide **1‐O** (Figure 5). Its structure features P−O−B fragment and can be described as an intramolecular Lewis adduct of the respective phosphine oxide.^[14]^ Accompanied formation of hydrogen in this reaction was observed by ^1^H NMR.

**Scheme 3:**
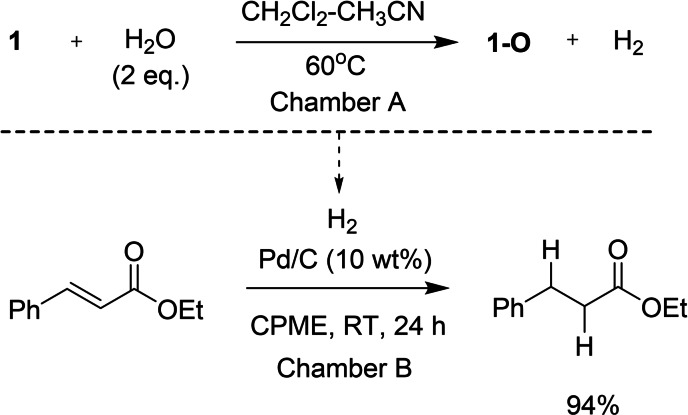
*In situ* generation of H_2_ via the stoichiometric reduction of H_2_O with **1** and its utilization as a reductant using a two‐chamber reactor.

To demonstrate that reduction of water with **1** is stoichiometric and produces an equimolar amount of hydrogen, we performed hydrogenation of ethyl cinnamate in a commercial two‐chamber reactor.^[15]^ Placed in one of the chambers a mixture of **1** with 2 equivalents of H_2_O served as the source of H_2_. The other chamber contained equimolar to **1** amount of ethyl cinnamate in cyclopentyl methyl ether (CPME) with Pd/C as a catalyst. Nearly quantitative (94 %) reduction of ethyl cinnamate to ethyl 3‐phenylpropanoate was observed after 24 h (Scheme 3).

Although formal oxidation of phosphines by water is a thermodynamically favourable process and is the main driving force for several synthetically important reactions such as Mitsunobu reaction^[16]^ or reductive disulfide bond cleavage,^[17]^ the direct reaction of water with phosphines under mild conditions is scarce. In this context, a bicyclic P(III) amidoester is remarkable for its ability to oxidatively add water and form P(V) derivative.^[18]^ We note that while our article was in preparation, an alike deoxygenation of water with stoichiometric amount of ortho‐phenylene linked bisborane‐functionalized phosphine was reported,^[14d]^ constituting the only other example of such chemistry to the best of our knowledge.

Considering other non‐metals, two metal‐free systems capable of H_2_O reduction to H_2_ have been reported, namely intramolecular silylene‐borane^[19]^ and sp^3^‐sp^3^ diboron compounds.^[20]^ Although the reports are lacking detailed mechanistic insights, heterolytic splitting of water is suggested as a key step in both cases.

We hypothesized that the bifunctional nature of **1** could trigger its reactivity with water. To verify the necessity of preorganized P/B sites for the observed unusual reactivity of **1**, we probed the reaction with a combination of separated FLP components chemically and structurally comparable to our system, namely PCy_3_ and B(2,6‐Cl_2_C_6_H_3_)_3_. Heating their equimolar mixture with 6 equivalents of H_2_O in a 1 : 1 CH_3_CN:CH_2_Cl_2_ mixture at 70 °C over night yielded no phosphine oxide (see Supporting Information for details).

### Mechanistic studies of water reduction with 1

**Scheme 4:**
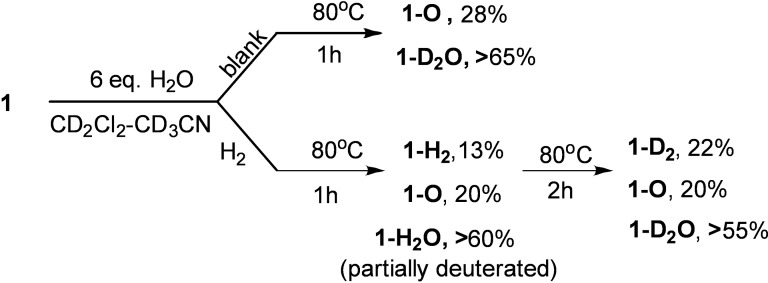
Transformations of **1‐H_2_O** upon heating with and without H_2_ pressure monitored by NMR spectroscopy (^1^H, ^11^B) in 1 : 1 CD_2_Cl_2_:CD_3_CN.

To elucidate mechanistic insights, we followed the kinetics of water reduction with **1** by ^31^P NMR spectroscopy and supported it by DFT computations. Inverse gated proton decoupling pulse sequence ensured quantitative measurements whereas utilization of non‐deuterated solvents prevented any isotopic exchange side effects. The samples were prepared in gas‐tight NMR tubes by dissolving **1** in 1 : 1 CH_3_CN/CH_2_Cl_2_ mixtures containing precise concentrations of water (Scheme 4). Kinetics of **1** reacting with variable H_2_O concentrations at 65 °C obey second order in **1‐H_2_O** and reverse first order in H_2_O. However, similar experiments carried out at 25 °C revealed first order in **1‐H_2_O** along with −0.5 order in water (see Supporting Information for details).

**Figure 6:**
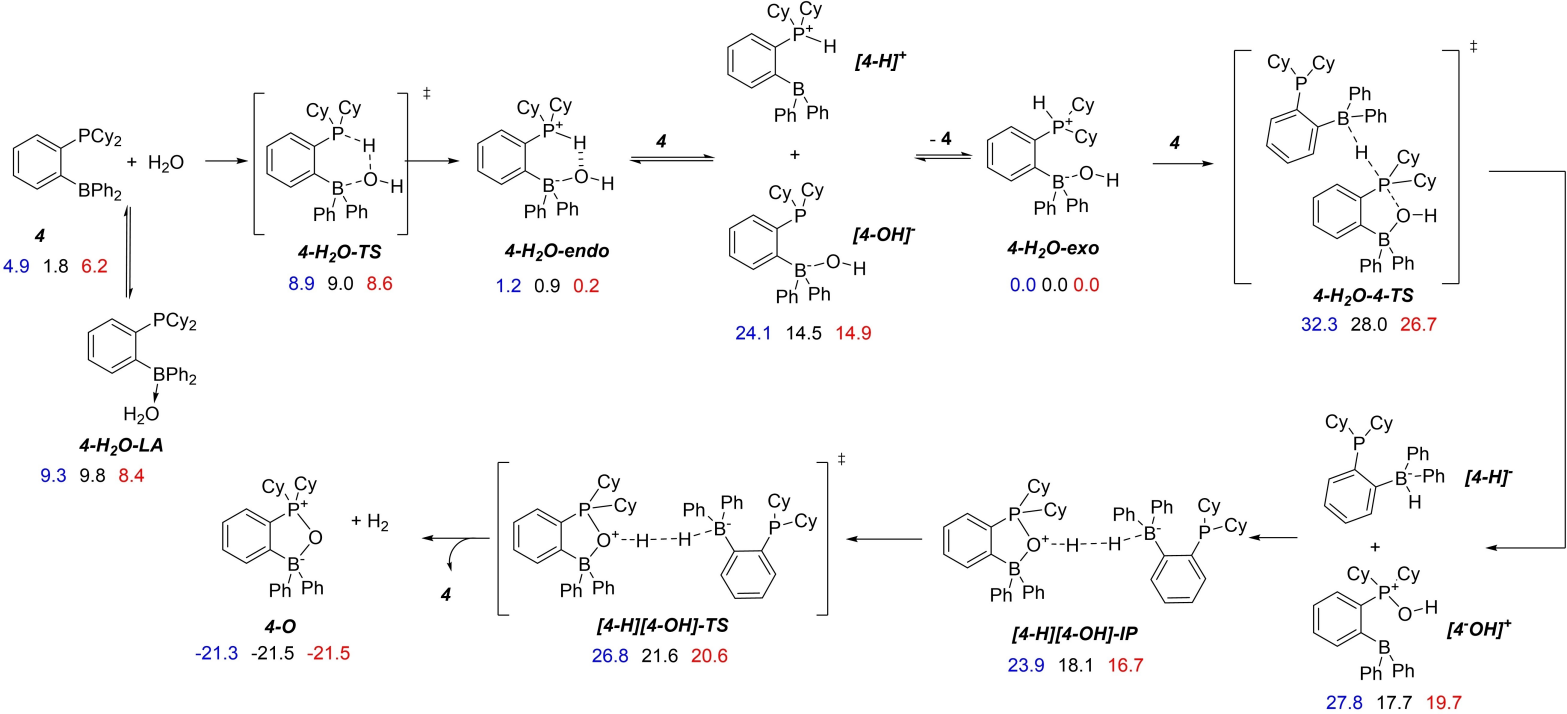
DFT studies of the water reduction mechanism with a model des‐chlorophosphinoborane **4**. Solution phase Gibbs free energies computed at the ωB97XD/6‐311++G(3df,3pd) level of theory (DFT) are given in kcal/mol with respect to **4‐H_2_O‐exo** in dichloromethane (blue), acetonitrile (black), and water (red).

In computational DFT studies we explored several mono‐ and bimolecular (with respect to phosphinoborane) mechanisms (Figure 6, see Supporting Information for details). In the interest of optimal utilization of computational resources, we modeled the water reduction mechanism using des‐chloro compound **4**. The possibility of unimolecular hydrogen release from **4‐H_2_O** via 4‐centered transition state **4 c‐TS** can be ruled out due to the high kinetic barrier (42.5 kcal/mol, Figure S52). Guided by kinetic results, we considered alternative mechanisms in which free **1** facilitates water reduction. We found that the LA center of **4** can accept a water‐derived hydrogen atom from the phosphine as a hydride. This “umpolung” of the protic P−H atom is accompanied by the migration of the OH group from the boron to the phosphorus atom inside the FLP pocket. The corresponding transition state **4‐H_2_O‐4‐TS** lies only 27.4 kcal/mol above **4**. The resultant phosphoxonium **[4‐OH]^+^** and borohydride **[4‐H]^−^** ions react further to give **4‐O**, **4**, and H_2_ via the proton‐hydride recombination. Notably, computations of a similar mechanism with triphenylborane as a hydride acceptor demonstrated the feasibility of a similar mechanism starting from the *endo*‐form of the water adduct **4‐H_2_O‐endo** (Figure S49). The respective alternative transition state was found to be higher in energy than the one starting from the *exo*‐adduct by 15 kcal/mol (see Supporting Information for details).

The results of the DFT computations were consistent with the kinetic experiments carried out at 65 °C. The most energetic transition state, the hydrogen atom transfer from **1‐H_2_O** to **1**, should manifest in the first kinetic orders in each of these compounds. Provided free **1** exists in rapid equilibrium with **1‐H_2_O** and water, the expected kinetic orders 2 in **1‐H_2_O** and −1 in H_2_O match the observed ones. The experimental data were fit to the above kinetic model via numeric kinetic simulations (see Supporting Information for details) with satisfactory accuracy and allowed for extracting of the kinetic parameters. We found the water reduction rate constant *k*=0.183 mM^−1^ h^−1^ and the equilibrium constant for the water dissociation *K*=2.86 mM or 3.9 kcal/mol, the latter was in good agreement with the value found by the DFT calculations. Notably, the reaction does not occur in pure CH_2_Cl_2_ at ambient temperatures or upon heating. However, the addition of free Lewis acid (C_6_F_5_)_3_B catalyzes the reaction at room temperature, which supports our mechanistic proposal. Water reduction by **1** is affected by a strong kinetic isotopic effect. Adduct of **1** with D_2_O, **1‐D_2_O** remains intact even upon prolonged heating in 1 : 1 CH_3_CN:CH_2_Cl_2_ mixtures.

As we noted above, an alike reactivity, namely a stoichiometric reduction of water with phosphine giving molecular hydrogen and phosphine oxide, was reported recently for an ortho‐phenylene linked bisborane‐functionalized phosphine, while our manuscript was under preparation.^[14d]^ A computational study presented in that study revealed conceptually the same mechanism as we proposed in our study: heterolityc splitting of water by phosphine and one Lewis acidic borane site followed by shuttling the borane‐bound OH group to the phosphorus center and concurrent abstraction of the hydride from P−H group by the second Lewis acidic boron center. Thus, the reaction reported by Shang et al.^[14d]^ represents an intramolecular version of the water reduction process providing a further support to our mechanistic proposal.

### Hydrogen addition to 1 in aqueous mixtures

**Figure 7:**
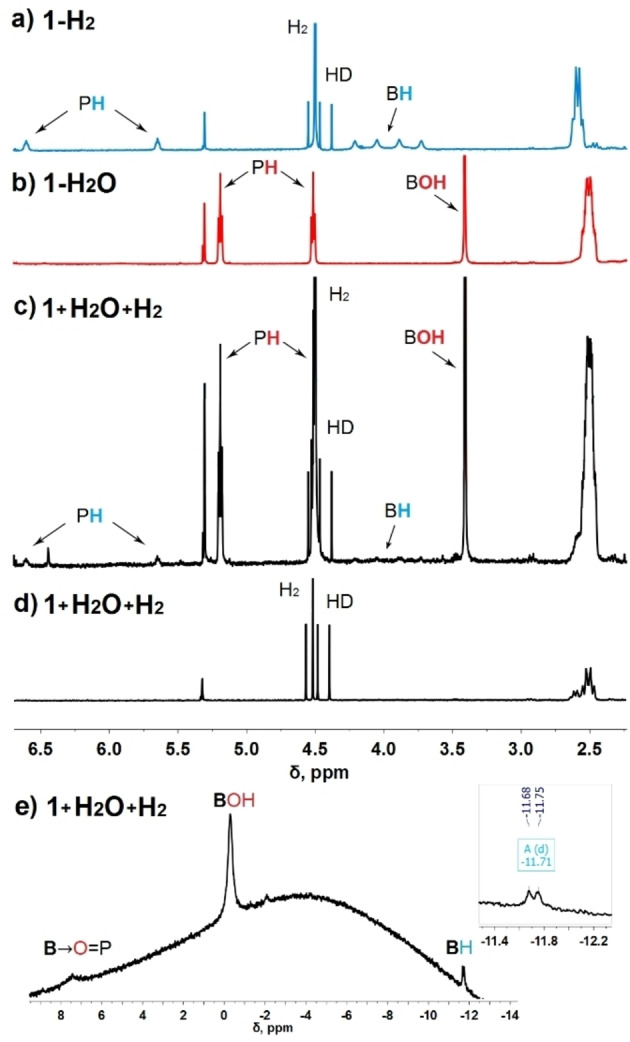
^1^H NMR spectra of **1‐H_2_** (a) and **1‐H_2_O** (b). (c) ^1^H NMR spectrum of **1** and 6 equivalents H_2_O after 1 h of heating at 80 °C under 10 bars of H_2_. ^1^H (d) and ^11^B (e) NMR spectra of the same sample recorded after additional 2 h of heating at 80 °C. All spectra were recorded in the 1 : 1 CD_2_Cl_2_:CD_3_CN mixture at 27 °C.

Since mechanistic studies indicated notable dissociation of **1‐H_2_O**, we further examined its reactivity in the 1 : 1 CD_2_Cl_2_:CD_3_CN mixture. In a gas‐tight heavy wall NMR tube, the solution of **1** and 6 equivalents of H_2_O was exposed to 10 bar of H_2_. A control sample was prepared in the same way but omitting H_2_. After 1 h of heating at 80 °C, both samples were analyzed by ^1^H, ^11^B, and ^31^P NMR spectroscopy (Scheme 4). The ^1^H NMR spectrum of the control sample (without H_2_) featured a set of signals corresponding to **1‐D_2_O** and newly formed **1‐O**. ^1^H NMR of the sample containing H_2_ revealed the presence of partially deuterated **1‐H_2_O**, **1‐O**, but also **1‐H_2_** was detected at low concentrations by the appearance of characteristic signals corresponding to the B−H and P−H hydrogens. (Figure 7c, signals of **1‐H_2_** are indicated). A triplet at 4.47 ppm corresponding to HD revealed isotope scrabbling arising from facile proton transfer between phosphonium centers and the deuterated solvent. After another 2 h of heating, corresponding to **1‐H_2_O** and **1‐H_2_** ^1^H NMR signals of exchangeable BO−H, P‐H, and B−H hydrogen atoms disappeared due to the complete deuteration. According to ^1^H and ^11^B NMR spectroscopies, the deuterated species **1‐D_2_O**, **1‐D_2_**, and **1‐O** were formed in the 3 : 1 : 1 ratio (Figure 7d, e).

### Parahydrogen experiments

**Figure 8:**
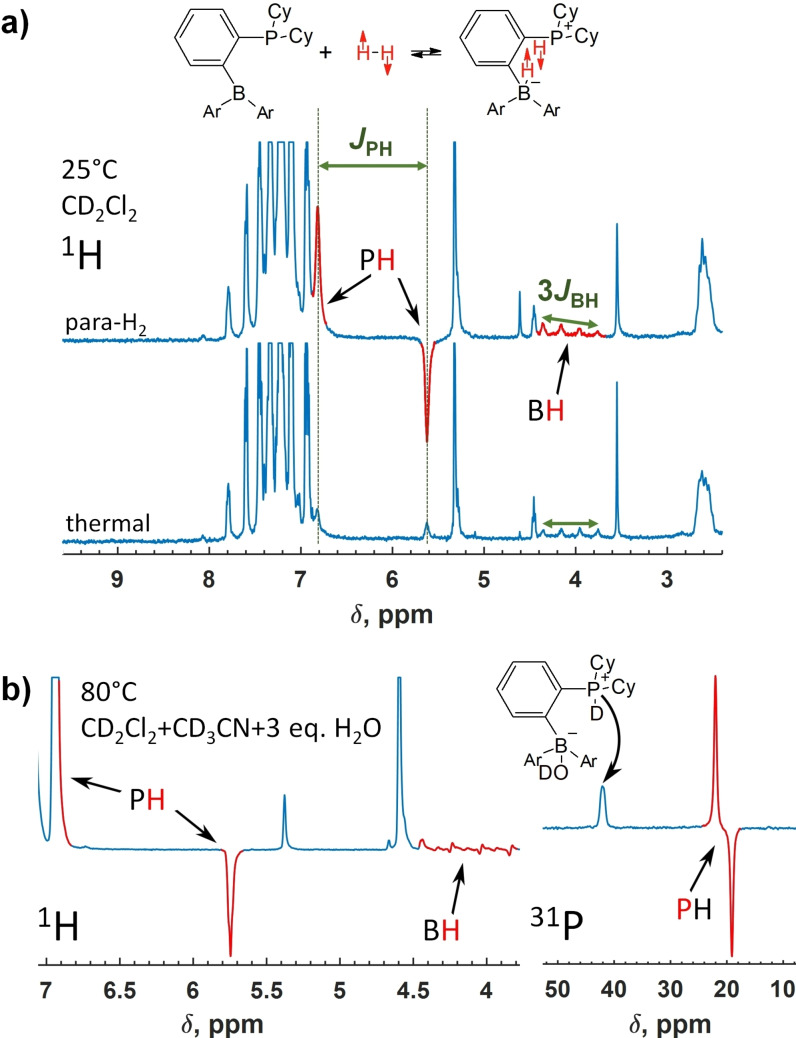
^1^H and ^31^P NMR spectra detected after exposing a 0.03 M solution of **1** in (a) dry and (b) moist (3 equivalents H_2_O) 1 : 1 CD_2_Cl_2_:CD_3_CN solvent at 25 and 80 °C, respectively. The signals revealing the hyperpolarization effects are colored in red. The intensity scale is adjusted for better visibility of the hyperpolarization.

The above‐described experiments revealed the formation of **1‐H_2_** in the presence of overstoichiometric amounts of water. Although **1‐H_2_** was not observed in the control experiment, the possibility of **1‐H_2_** to be an unstable intermediate of the background water reduction process during high pressure H_2_ experiments could not be ruled out completely. To decouple the formation of **1‐H_2_** via direct addition of H_2_ to **1** from the mediation of the water reduction mechanism, we studied the interaction of **1** with parahydrogen. Detection of hyperpolarized **1‐H_2_** in the presence of water would prove the direct H_2_ activation mechanism because hyperpolarization is rapidly destroyed during secondary processes. We found that the interaction of *ansa*‐phosphinoborane **1** with parahydrogen leads to nuclear spin hyperpolarization effects in both dry and aqueous solvents. In the first experiments, we bubbled parahydrogen through a 0.05 M solution of **1** in a dry CD_2_Cl_2_ at 295 K under 3.2 bar pressure. The corresponding ^1^H NMR spectra after the bubbling and after the relaxation to thermal equilibrium showed an enhanced signal for the P−H group with splitting equal to the corresponding spin‐spin coupling constant (*J*_PH_=478.0 Hz) (Figure 8a). The individual components of the doublet show opposite signs, revealing the created non‐equilibrium nuclear spin order. The B−H group signal appearing as a 1 : 1 : 1 : 1 quartet under the thermal equilibrium due to ^11^B nuclei (*J*_BH_=79.5 Hz, spin 3/2) develops a slight distortion of the multiplet structure after the parahydrogen bubbling that also indicates the non‐equilibrium state.

In the next experiments, **1** was dissolved in the aqueous solvent (3 equivalents H_2_O in 1 : 1 CH_3_CN:CH_2_Cl_2_), letting the complete transformation of **1** into **1‐H_2_O**. Neither **1‐H_2_** nor hyperpolarization effects were observed while bubbling parahydrogen at 25 °C through this solution. Heating to 80 °C, however, unfroze the **1‐H_2_O** dissociation that enabled the formation of hyperpolarized **1‐H_2_** (Figure 8b), confirming the reversibility of interaction of **1** with both H_2_O and H_2_. As in the dry solvent, ^1^H hyperpolarization was observed for the P−H and B−H hydrogens with some differing fine details such as the shapes of the B−H multiplet. In addition to ^1^H, ^31^P hyperpolarization was detected for **1‐H_2_** that manifested as an antiphase doublet in ^31^P NMR (Figure 8b, right).

We note that these hyperpolarization effects differ from those commonly observed in high magnetic fields in PASADENA experiments.[^7b^, ^21^] Normally, only ^1^H NMR multiplets resulting from the homonuclear *J*‐coupling between two parahydrogen nascent ^1^H nuclei reveal the hyperpolarization. In contrast, heteronuclear *J*‐couplings (^31^P‐^1^H and much less ^11^B‐^1^H) reveal the hyperpolarization in our case. Practically, it means that ^31^P (and slightly ^11^B) nuclei are hyperpolarized in addition to ^1^H. The mechanism of this effect must involve relaxation‐driven transitions between different magnetization modes similarly as it was described for ^1^H, ^15^N, and ^11^B hyperpolarization in *ansa*‐aminoboranes.[^7c^, ^7d^]

## Conclusion

New *ansa*‐phosphinoborane **1** features the ability for the reversible heterolytic splitting of H_2_ and H_2_O. The zwitterionic water adduct **1‐H_2_O** can release H_2_ through a multistep reaction pathway and form heterocyclic oxide **1‐O**. The hydrogen atom “umpolung” mechanism of the reaction was investigated experimentally and computationally, and dissociation of H_2_O from the adduct **1‐H_2_O** was shown to be vital for the observed reactivity. The nuclear spin hyperpolarization in parahydrogen experiments indicated that the addition of H_2_ to *ansa*‐phosphinoborane **1** is pairwise, meaning that the H atoms do not lose each other in the H_2_ activation process. It also confirmed the reversibility of H_2_O and H_2_ additions, supporting the viability of dissociation steps in the proposed mechanism of the water reduction by the *ansa*‐phosphinoborane. Moreover, this is the first time FLPs showing hyperpolarization effects in the presence of excess H_2_O, providing proof that metal‐free activators for parahydrogen can be moisture tolerant.

## Conflict of interest

The authors declare no conflict of interest.

1

## Supporting information

As a service to our authors and readers, this journal provides supporting information supplied by the authors. Such materials are peer reviewed and may be re‐organized for online delivery, but are not copy‐edited or typeset. Technical support issues arising from supporting information (other than missing files) should be addressed to the authors.

Supporting Information

## Data Availability

Deposition Numbers 2109878 (for **1**), 2109879 (for **1‐H**_**2**_ **O**), 2109880 (for **1‐O**) contain the supplementary crystallographic data for this paper. These data are provided free of charge by the joint Cambridge Crystallographic Data Centre and Fachinformationszentrum Karlsruhe Access Structures service.
