# Supplementary material for: Water Reduction and Dihydrogen Addition in Aqueous Conditions With ansa‐Phosphinoborane
Source: Chemistry. 2022 Aug 31;28(61):e202201927. doi: 10.1002/chem.202201927 (PMC9804508; doi:10.1002/chem.202201927)
Supplement: Supplementary file 1 — Supporting Information [file CHEM-28-0-s001.pdf]

Chemistry–A European Journal

Supporting Information

Water Reduction and Dihydrogen Addition in Aqueous Conditions With *ansa*-Phosphinoborane

Kristina Sorochkina, Konstantin Chernichenko,* Vladimir V. Zhivonitko, Martin Nieger, and Timo Repo*

Contents

General experimental S3

Synthesis and Characterization S3

Reaction of 1 with H_2/D_2 .... S20

NMR Monitoring of PCy_3/ H_2O /$B(2,6\text{-}Cl_2C_6H_3)_3$ in the solution. S21

Hydrogen activation by 1 in presence of water........ S25

Two-chamber experiment S30

Crystal Structure Determinations of 1, 1-H_2O and 1-O S31

Kinetic studies........ S34

Sample preparation and procedure S34

25 °C experiments........ S34

65 °C experiments........ S35

Kinetic modeling S36

Kinetic modeling report:........ S37

DFT calculations S40

Methods S40

Hydrogen and water addition to 1 .... S40

Optimized geometries of derivatives of 1: S41

Computationally modelled mechanism of water reduction by 4 as model phosphinoborane and triphenylborane (5) as a catalyst S43

Computational modeling of water reduction mechanism with 4 as model phosphinoborane S43

Computational study of monomolecular water reduction mechanisms S45

Optimized geometries of derivatives of 4 and 5........ S46

Tables of energies S52

XYZ coordinates S54

Parahydrogen experiments........ S101

References........ S102

General experimental

All operations with air- or moisture-sensitive compounds were performed under argon atmosphere by a conventional Schlenk technique or in a glove box (Mbraun Unilab). Solvents were dried using a VAC solvent purification system, additionally degassed if needed, and stored over 3Å molecular sieves in a glovebox. Deuterated solvents were purchased from Eurisotop and used without additional purification or, if needed, dried by standing over molecular sieves (3 Å) and stored in the glovebox. Reagents were purchased from Sigma-Aldrich or ABCR and used as received. 1,3-dichloro-2-iodobenzene (**2**)[1] and $B(2,6\text{-}Cl_2C_6H_3)_3$[2] were synthesized according to the reported literature methods. Hydrogen (5.0) was purchased from Oy Aga Ab and used as such. Gas-tight (J. Young valve) NMR tubes were purchased from Wilmad.

^{1}H, ^{13}C, ^{11}B, ^{31}P NMR spectra were recorded at Bruker Advance Neo 500 spectrometers at 27 °C, if not otherwise stated. Chemical shifts are quoted in δ (ppm) and coupling constants in Hz. All ^{13}C NMR spectra were proton-decoupled; proton decoupled ^{11}B and ^{31}P NMR spectra are indicated as $^{11}B\{^{1}H\}$ or $^{31}P\{^{1}H\}$ respectively. Chemical shifts were referenced internally to residual protio-solvent (^{1}H) or solvent (^{13}C) resonances and are reported relative to tetramethylsilane (δ = 0 ppm), ^{31}P resonances are referenced externally to H_3PO_4 (85 %), and ^{11}B chemical shifts to $BF_3{\cdot}Et_2O$.

Elemental analyses were performed with vario Micro cube instrument in CHN mode.

Synthesis and Characterization

Chlorobis(2,6-dichlorophenyl)borane (3)

At -78 °C, a solution of *n*-BuLi (4.6 ml, 1.6 M in hexane, 7.3 mmol) was added dropwise to a solution of 1,3-dichloro-2-iodobenzene (**2**) (2.0 g, 7. mmol) in Et_2O (15 ml). The reaction mixture was stirred at -78 °C for 5 minutes. The reaction mixture was cooled down to -90 °C and a solution of BCl_3 (3.7 ml, 1 M in hexane, 3.7 mmol) was added via syringe. The mixture was allowed to warm to room temperature and was left stirred overnight. Evaporation of the solvent to dryness under vacuum of oil pump gave the product with sufficient purity as pale yellow crystalline solid (2.47 g, >95%). The product was used in the following step without further purification.

^{1}H NMR (500 MHz, CD_2Cl_2, 25°C): δ 7.34.

^{1}H NMR (500 MHz, C_6D_6, 25°C): δ 6.79 (d, J_{HH} = 8.1 Hz, 4H), 6.42 (t, J_{HH} = 8.1 Hz, 2H).

^{13}C NMR (126 MHz, CD_2Cl_2, 25°C): δ 161.35, 156.13, 151.91.

^{11}B NMR (160 MHz, C_6D_6, 25°C): δ 65.78.

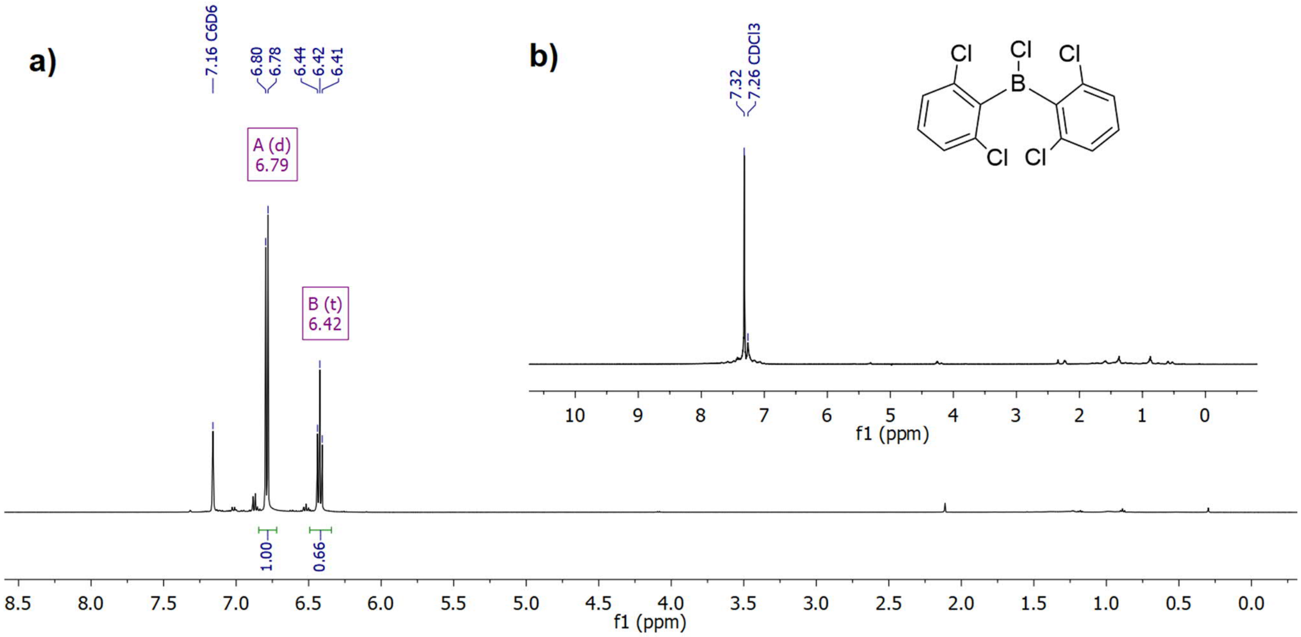

Figure S1.^{1}H NMR (500 MHz, 25°C) spectrum of compound **2 (a)** in C_6D_6 and **(b)** in $CDCl_3$.

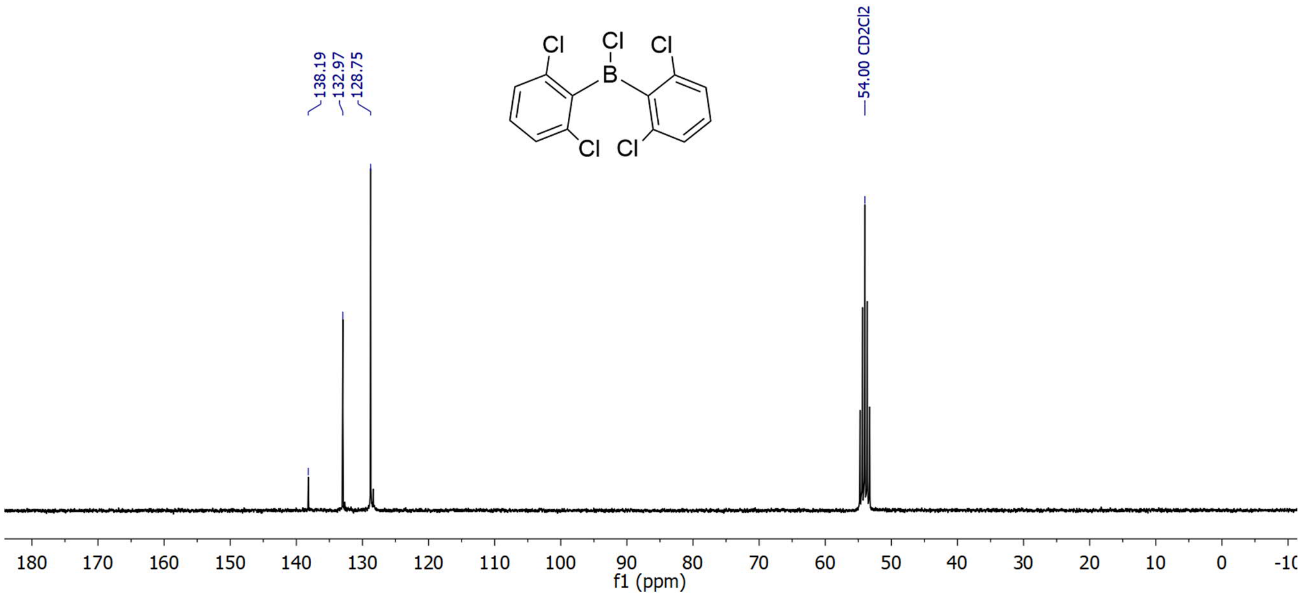

Figure S2.^{13}C NMR (126 MHz, CD_2Cl_2, 25°C) spectrum of compound **2**.

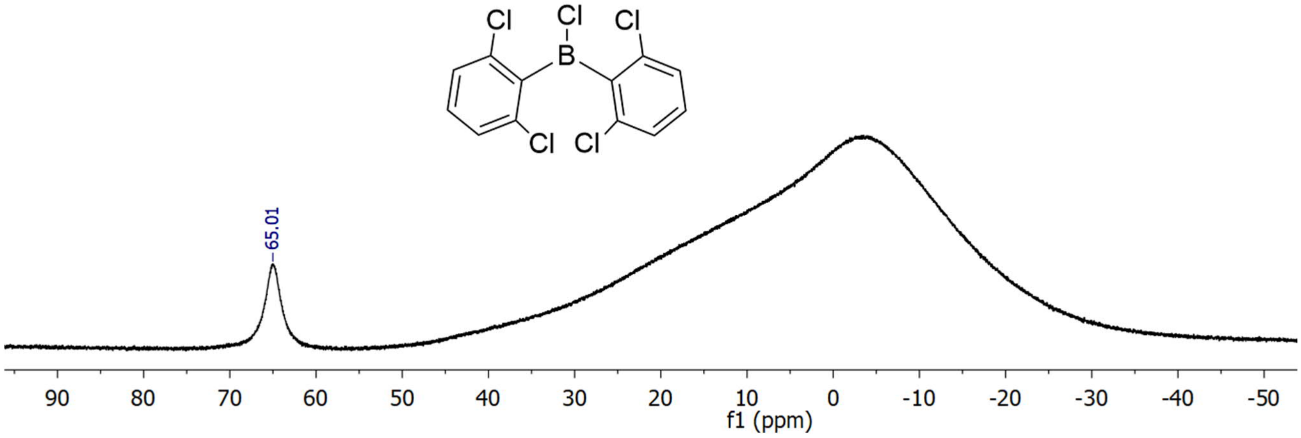

Figure S3. ^{11}B NMR (160 MHz, C_6D_6, 25°C) spectrum of compound **2**.

(2-(bis(2,6-dichlorophenyl)boraneyl)phenyl)dicyclohexylphosphane (1)

At - 78°C, a solution of *t*-BuLi (3.3 ml, 1.7 M, 5.7 mmol) was added dropwise to a solution of (2-bromophenyl)dicyclohexylphosphane (1.0 g, 2.8 mmol) in Et_2O (10 ml). The reaction mixture was stirred at -78°C for 1 hour, then warmed up to room temperature and stirred for 30 minutes. The reaction mixture was cooled down to –90°C and a solution of chlorobis(2,6-dichlorophenyl)borane (960 mg, 2.8 mmol) in 7 ml Et_2O was added via syringe. The mixture was allowed to warm to room temperature and was left stirred overnight. Then the solvent was removed to dryness under vacuum of oil pump, the residue was suspended in 10 ml of dry toluene and filtered through a glass filter. Filter cake was washed with toluene (5ml×3). The combined filtrate was evaporated under vacuum of oil pump to dryness. The residue was recrystallized from the mixture 1:1 hexane:toluene (10 ml) at -20°C to give spectroscopically pure product as yellow crystals (848 mg, 52%).

^{1}H NMR (500 MHz, CD_2Cl_2, 25°C): δ 7.60 (dd, J_{HH} = 7.6, 2.7 Hz, 1H, C_6H_4), 7.45 (td, J_{HH} = 7.5, 1.7 Hz, 1H, C_6H_4), 7.37-7.29 (2H, C_6H_4), 7.23(m, 6H, Cl_2Ph), 1.79 (m, 4H, Cy), 1.70 (m, 2H, Cy), 1.60 (m, 4H, Cy), 1.44 (m, 2H, Cy), 1.24-1.04(m, 8H, Cy), 0.95 (m, 2H, Cy).

^{13}C NMR (126 MHz, CD_2Cl_2, 25°C): δ 154.04 (br, C-B, C_6H_4),144.61 (d, J_{CP} = 14.4 Hz, C-P, C_6H_4), 143.91 (brs, C-B, Cl_2Ph), 138.86 (C-Cl_{ortho}), 135.65 (d, J_{CP} = 15.3 Hz, C_6H_4), 132.34 (d, J_{CP} = 2.7 Hz, C_6H_4), 131.33(*p*-C,Cl_2Ph), 130.88 (C_6H_4), 128.72 (d, J_{CP} = 0.9 Hz, C_6H_4), 128.16 (*m*-C, Cl_2Ph), 35.98 (d, J_{CP} = 14.8 Hz, Cy), 31.47 (d, J_{CP} = 15.0 Hz, Cy), 30.09 (d, J_{CP} = 11.4 Hz, Cy), 28.12 (d, J_{CP} = 11.1 Hz, Cy), 27.89 (d, J_{CP} = 8.9 Hz, Cy), 27.00 (Cy), C-B (Cl_2Ph) is not observed.

gHSQC (F2/F1, ^{1}H/^{13}C, 500/126 MHz, CD_2Cl_2, 25°C): δ 7.34/135.76, 7.59/132.47, 7.23/131.45, 7.44/131.07, 7.32/128.74, 7.23/128.28, 1.79/36.17, 0.96/31.59, 1.44/31.56, 1.12/30.26, 1.80/30.20, 1.60/28.26, 1.11/28.19, 1.70/28.07, 1.18/27.91, 1.60/27.13, 1.11/27.07

^{31}P NMR (121 MHz, CD_2Cl_2, 25°C): δ 0.51 (s).

^{11}B NMR (160 MHz, C_6D_6, 25°C): δ 67.03 (brs).

Elemental microanalysis: calc. for $C_{30}H_{32}BCl_4P$ + 0.1 Toluene, C 62.98% H 5.66% Elem. found: C 63.18 % H 5.89 %

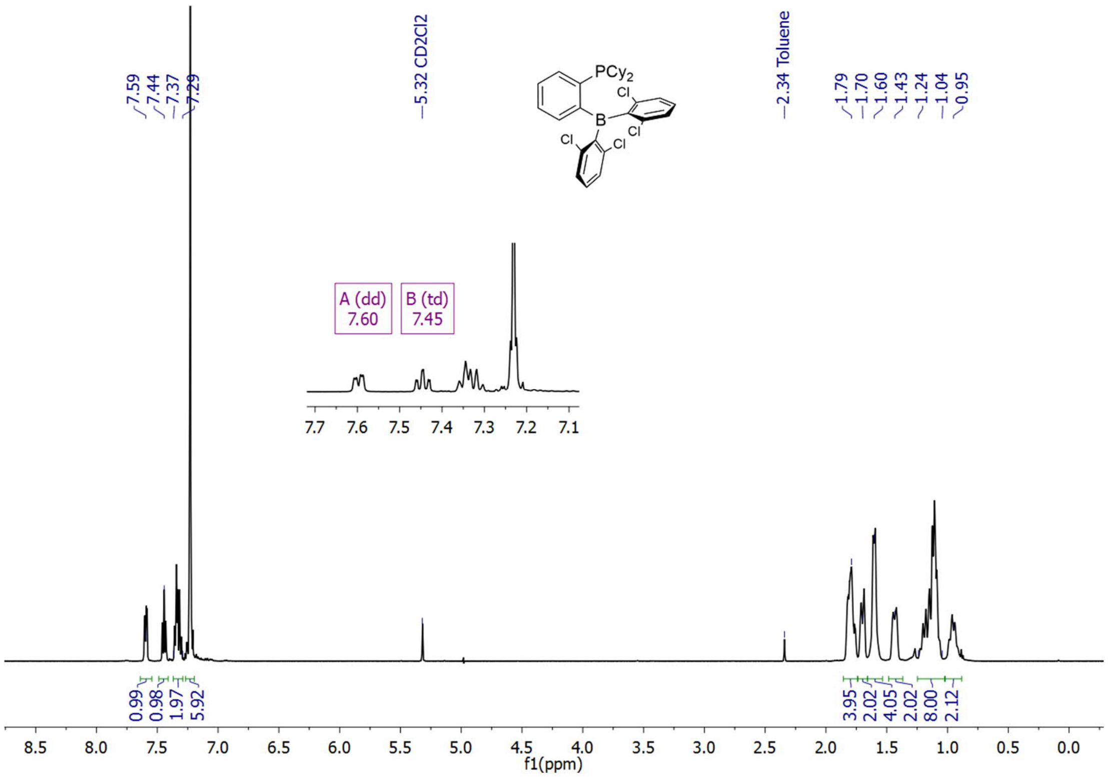

Figure S4.^{1}H NMR (500 MHz, CD_2Cl_2, 25°C) spectrum of compound **1**.

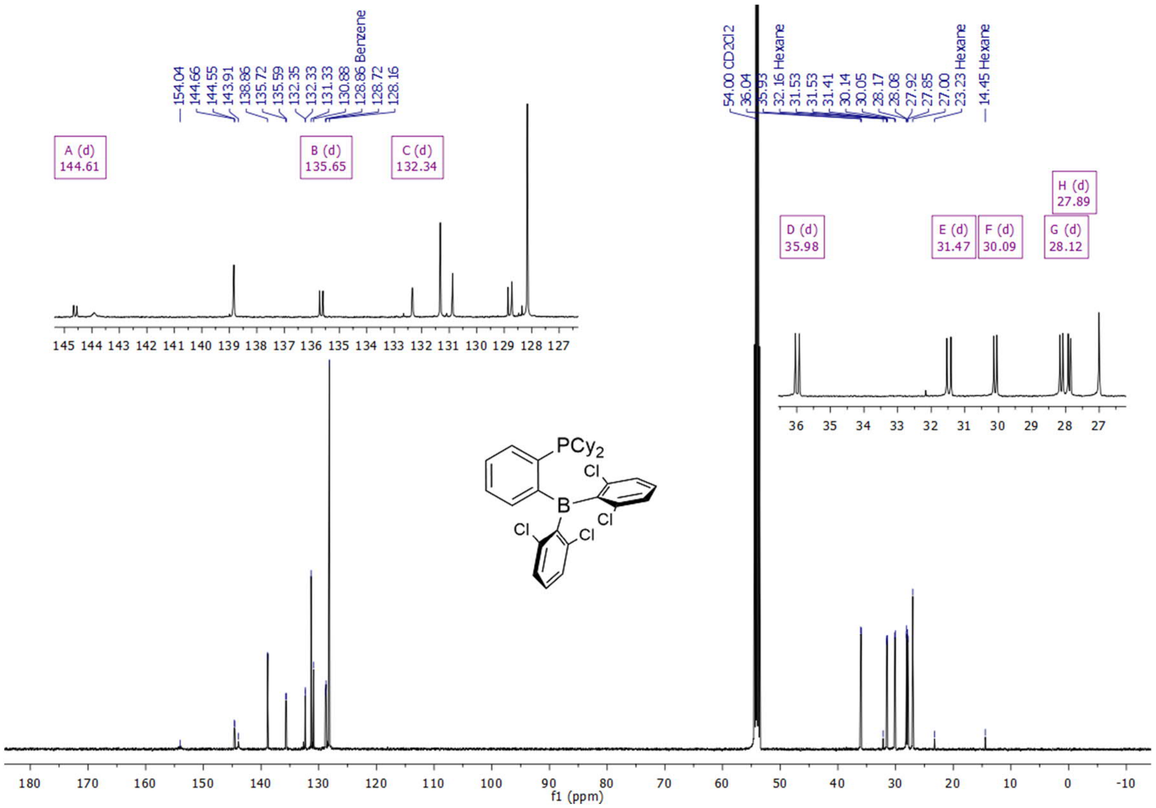

Figure S5.^{13}C NMR (126 MHz, CD_2Cl_2, 25°C) spectrum of compound **1**.

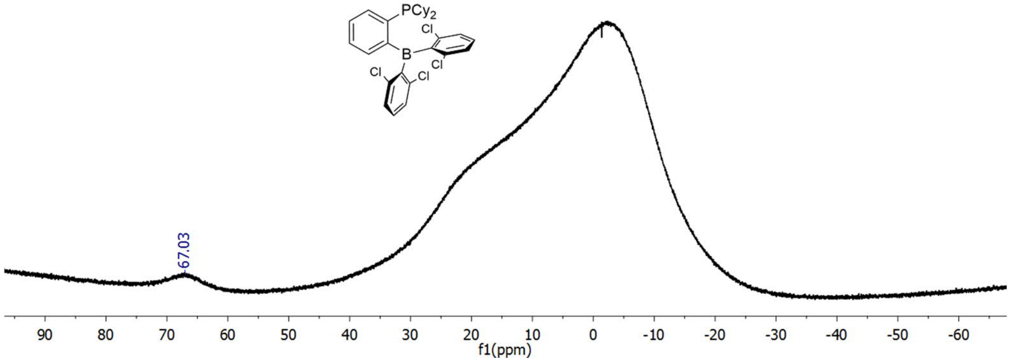

Figure S6. ^{11}B NMR (160 MHz, CD_2Cl_2, 25°C) spectrum of compound **1**.

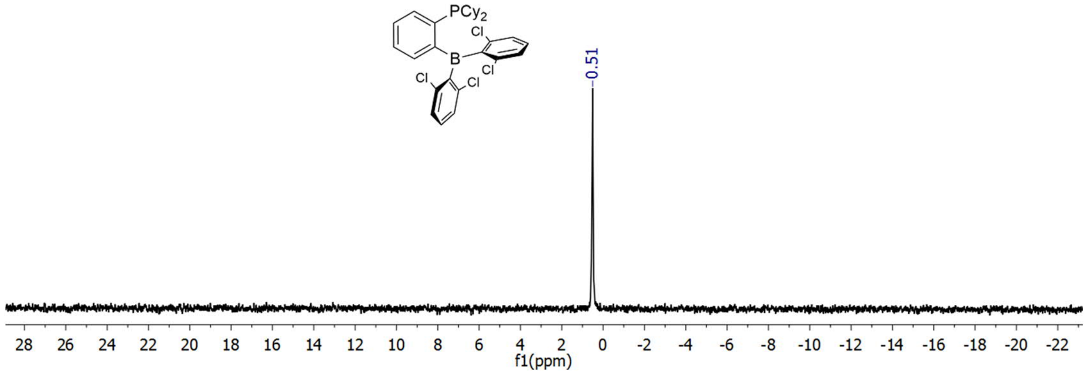

Figure S7. ^{31}P NMR (121 MHz, CD_2Cl_2, 25°C) spectrum of compound **1**.

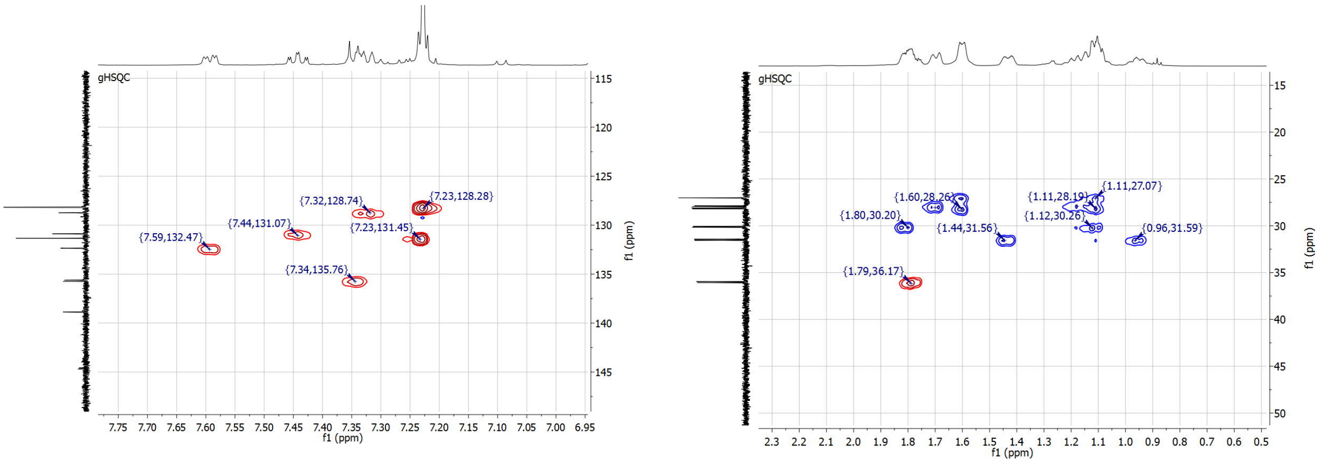

Figure S8. gHSQC NMR (500/126 MHz, CD_2Cl_2, 25°C) spectrum of compound **1** (aromatic(left, bottom) and aliphatic(right, top) regions).

Hydrogen addition to 1 (synthesis of 1-H_2)

In a glovebox, a thick-wall gas-tight NMR tube was charged with **1** (7 mg, 11.8 μmol) and 0.3 ml of CD_2Cl_2. The sample was then taken out of the glovebox and pressurized with 10 bars of dihydrogen. Full conversion of **1** to **1-H_2** was observed according to heteronuclear NMR analysis.

^{1}H NMR (500 MHz, CD_2Cl_2, 25°C): δ 7.46 (brm, 1H, C_6H_4), 7.32 (m, 1H, C_6H_4), 7.25 (m, 1H, C_6H_4), 7.19 (m, 1H, C_6H_4), 7.12 (d, J_{HH} =7.8, 4H), 6.93 (t, J_{HH} = 7.9 Hz, 2H), 6.22 (brd, J_{HP} = 478.0 Hz, 1H), 4.06 (q, J_{HB} = 79.5 Hz, 1H), 2.64 (brm, 2H, Cy), 2.05(brm, 2H, Cy), 1.96(brm, 2H), 1.88-1.64 (brm, 6H, Cy), 1.44 (m, 2H, Cy), 1.36-1.14 (brm, 8H, Cy).

^{13}C NMR (126 MHz, CD_2Cl_2, 25°C): δ 168.11 (br, C-B, C_6H_4), 153.49 (br, C-B, Cl_2Ph), 142.68 (*o*-CCl), 138.30 (d, J_{CP} = 14.5 Hz), 132.43 (s, C_6H_4), 130.42 (brs, C_6H_4), 128.24 (*m*-C, Cl_2Ph), 126.49 (*p*-CCl), 124.78 (d, J_{CP} = 13.4 Hz), 118.72 (d, J_{CP} = 82.4 Hz), 31.23 (d, J_{CP} = 43.0 Hz Cy), 26.85 (d, J_{CP} = 13.4 Hz, Cy), 26.70 (d, J_{CP} = 13.2 Hz, Cy), 25.83 (d, J_{CP} = 1.4 Hz, Cy).

^{31}P NMR (202 MHz, CD_2Cl_2, 25°C): δ 19.59 (brs).

^{11}B NMR (160 MHz, C_6D_6, 25°C): δ -11.79 (d, J_{BH} = 80.6 Hz).

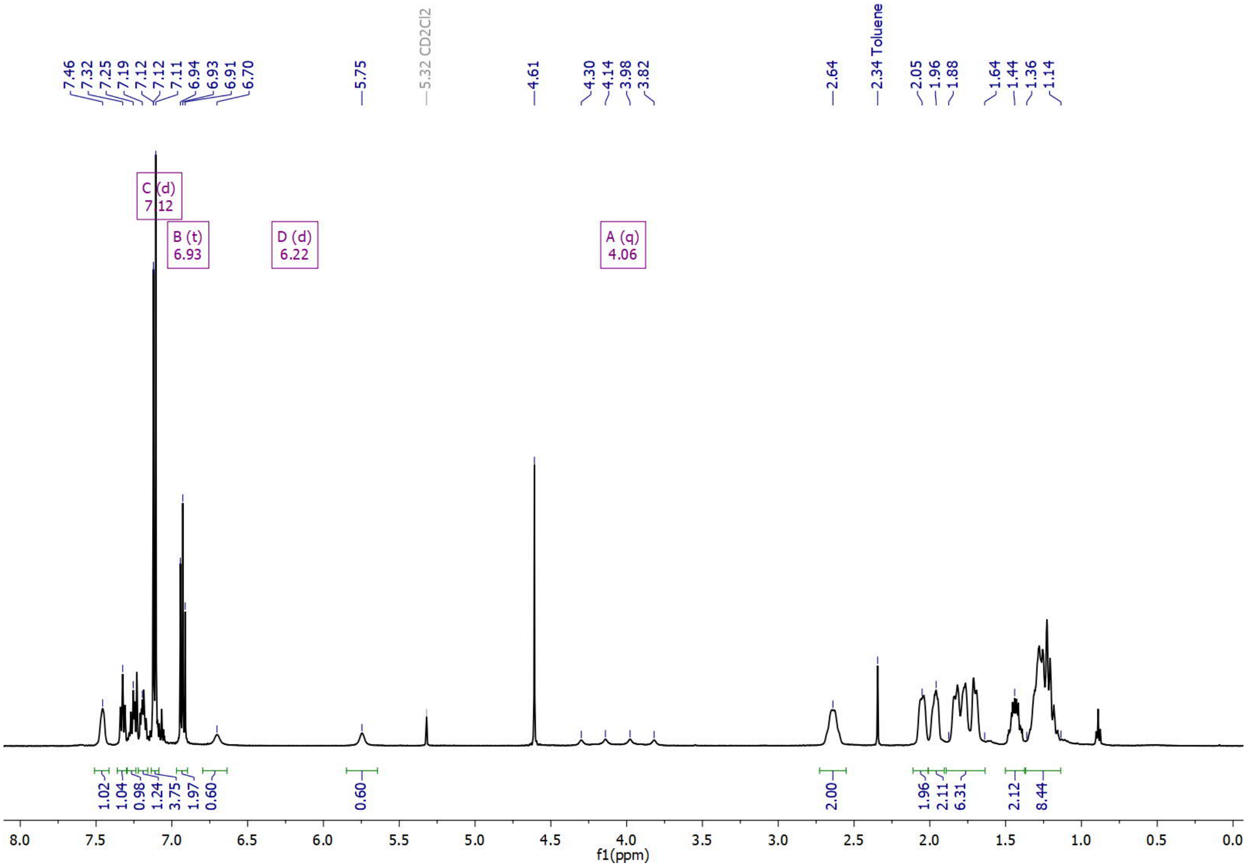

Figure S9. ^{1}H NMR (500 MHz, CD_2Cl_2, 25°C) spectrum of compound **1-H_2**.

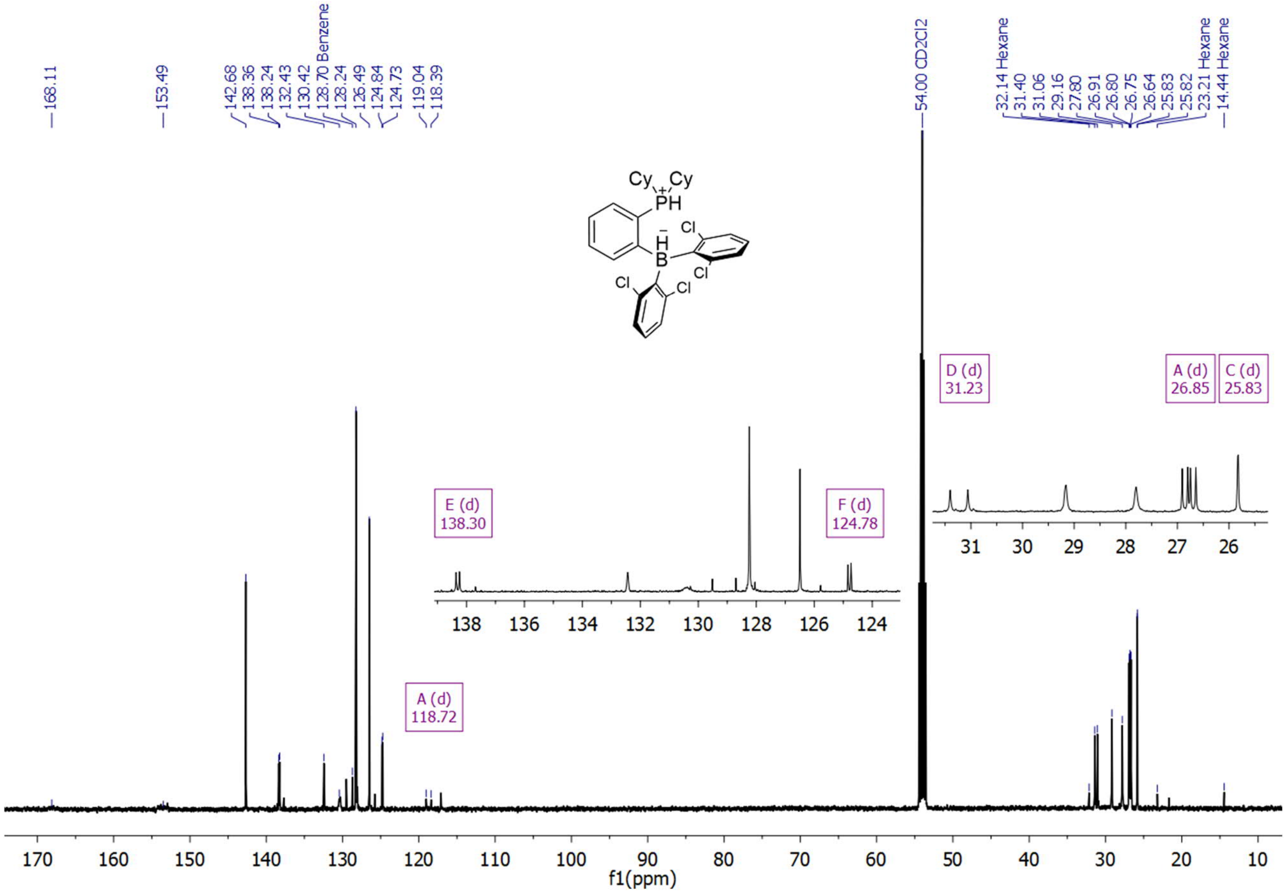

Figure S10. ^{13}C NMR (126 MHz, CD_2Cl_2, 25°C) spectrum of compound **1-H$_2$**.

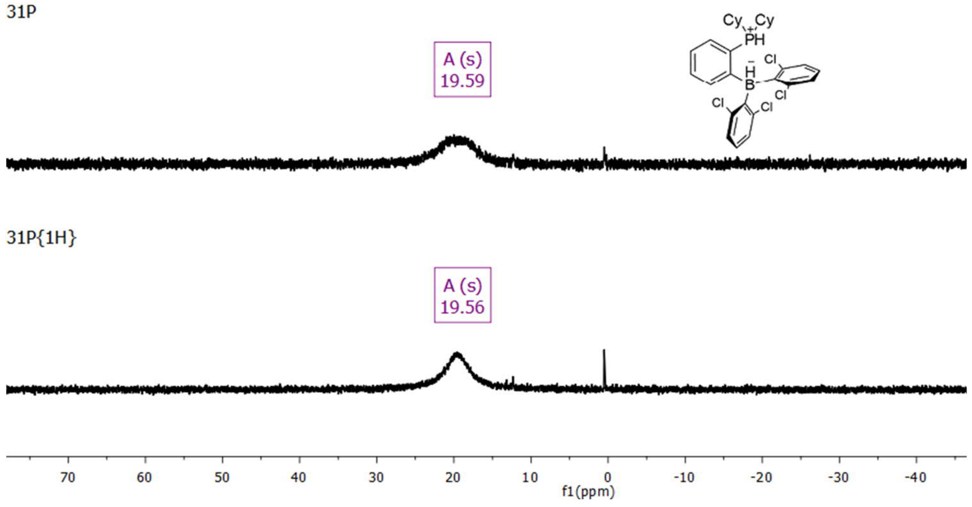

Figure S11. Proton-coupled (top) and proton-decoupled (bottom) ^{31}P NMR (121 MHz, CD_2Cl_2, 25°C) spectra of compound **1-H$_2$**.

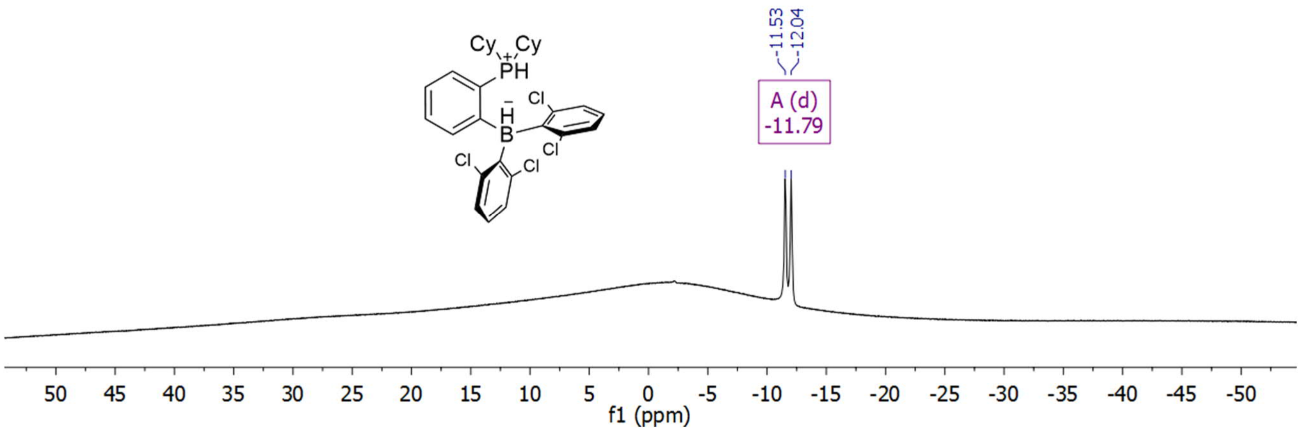

Figure S12. ^{11}B NMR (160 MHz, CD_2Cl_2, 25°C) spectrum of compound **1-H$_2$**.

Deuterium addition to 1 (synthesis of 1-D$_2$)

In a glovebox, a thick-wall gas-tight NMR tube was charged with **1** (7 mg, 11.8 μmol) and 0.3 ml of CD_2Cl_2. The sample was then taken out of the glovebox and pressurized with 5 bars of dideuterium. Full conversion of **1** to **1-D$_2$** was observed according to heteronuclear NMR analysis.

^{1}H NMR (500 MHz, CD_2Cl_2, 25°C): δ 7.46 (brm, 1H, C_6H_4), 7.33 (m, 1H, C_6H_4), 7.24 (m, 1H, C_6H_4), 7.19 (m, 1H, C_6H_4), 7.11 (d, J_{HH} =7.8, 4H), 6.93 (t, J_{HH} = 7.9 Hz, 2H), 2.64 (brm, 2H, Cy), 2.05(brm, 2H, Cy), 1.96(brm, 2H), 1.88-1.64 (brm, 6H, Cy), 1.44 (m, 2H, Cy), 1.36-1.14 (brm, 8H, Cy).

^{31}P NMR (202 MHz, CD_2Cl_2, 25°C): δ 17.95 (brs).

^{11}B NMR (160 MHz, C_6D_6, 25°C): δ -12.01 (s).

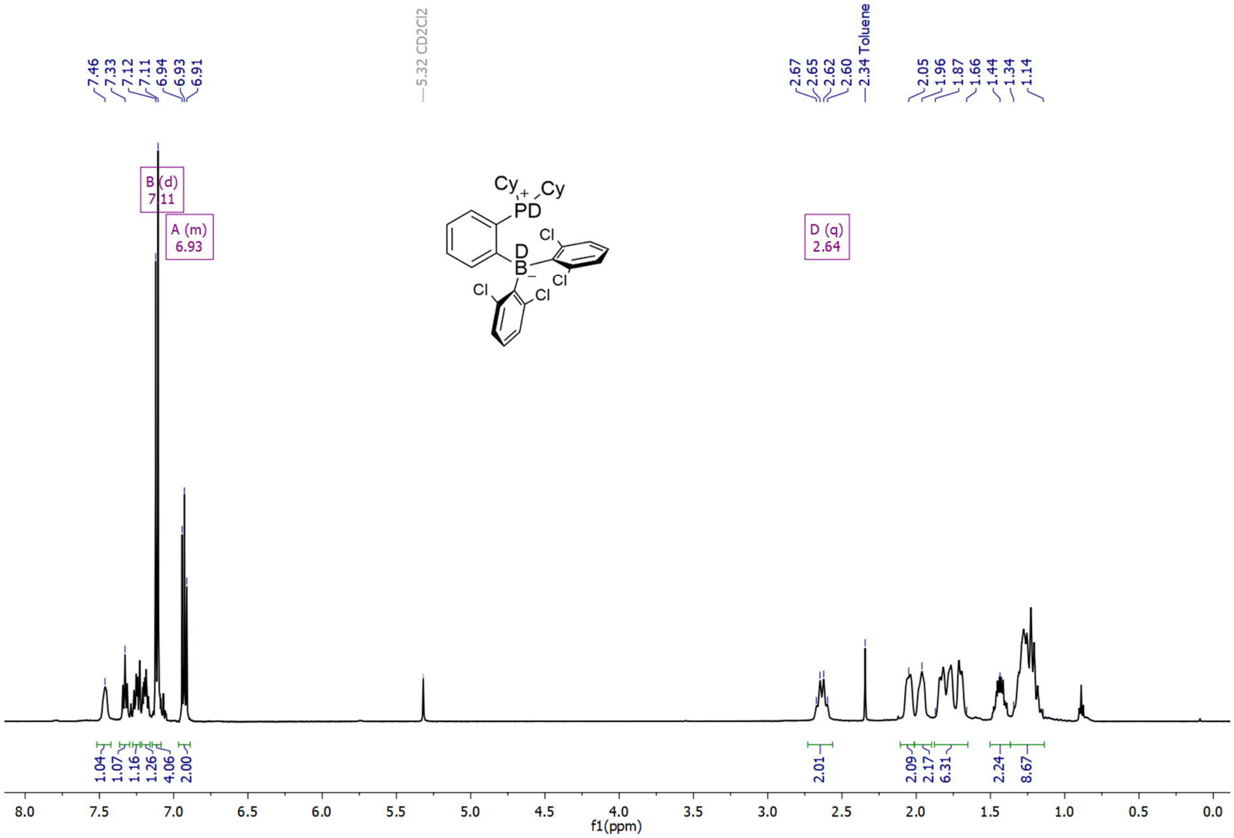

Figure S13. ^{1}H NMR (500 MHz, CD_2Cl_2, 25°C) spectrum of compound **1-D$_2$**.

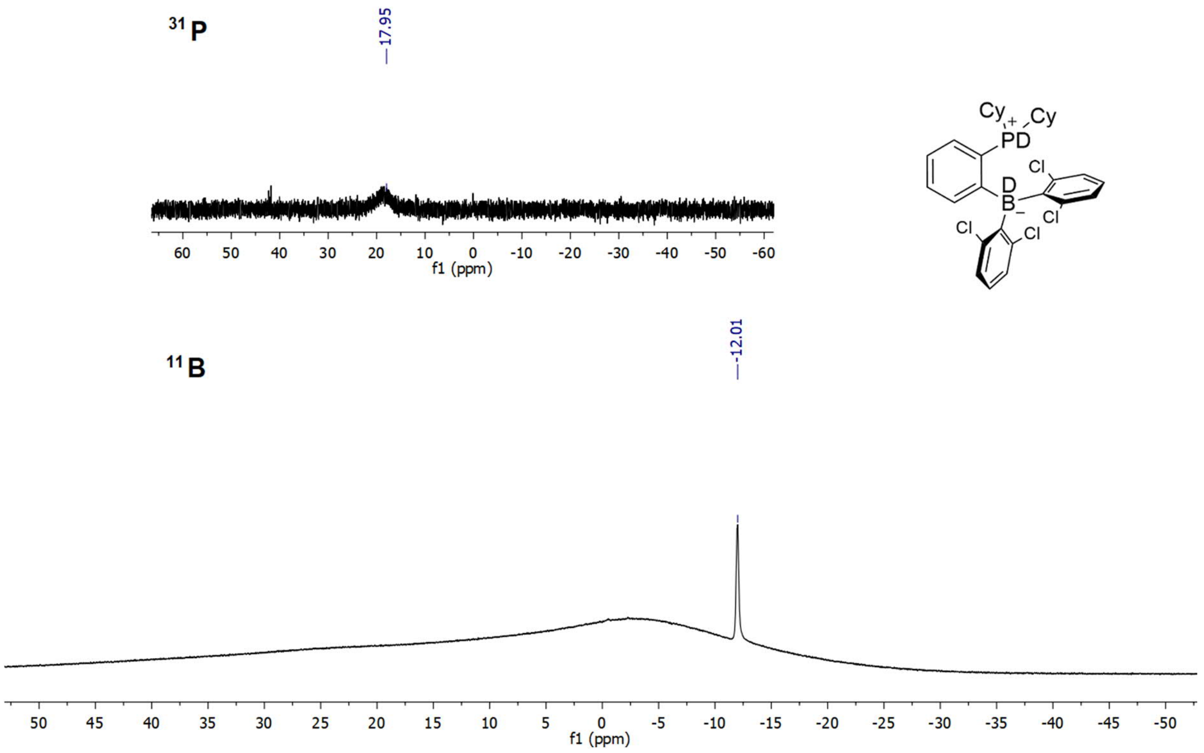

Figure S14. ^{31}P NMR (121 MHz, CD_2Cl_2, 25°C)(top) and ^{11}B NMR (160 MHz, CD_2Cl_2, 25°C)(bottom) spectra of compound **1-D$_2$**.

Water addition to 1 (synthesis of 1-H_2O).

In a glovebox, 10 ml Schlenk tube was charged with **1** (100mg, 0.17mmol), 3 equivalents of water (~9 mg) and 3 ml of CD_2Cl_2. The reaction mixture was stirred for 10 minutes. The solvent was evaporated to dryness under the vacuum of the oil pump to give the product quantitatively as a colorless crystalline solid.

^{1}H NMR (500 MHz, CD_2Cl_2 , 25°C): δ 7.80 (m, 1H, C_6H_4), 7.43 (dd, J_{HP}= 16.9, J_{HH} =7.6 Hz, 1H), 7.37 (m, 1H, C_6H_4), 7.20 (m, 1H, C_6H_4), 7.10 (d, J_{HH} = 7.9 Hz, 4H, $Cl_2C_6H_3$), 6.94 (t, J_{HH} = 8.0 Hz 2H, $Cl_2C_6H_3$), 4.87 (dt, J_{HP} = 332.7, J_{HH} = 6.0 Hz, 1H, PH), 3.55 (s, 1H, OH), 2.56(m, 2H, Cy), 2.10 (brm, 2H, Cy), 1.88 (brm, 2H, Cy), 1.78-1.54(brm, 6H, Cy+H_2O), 1.39 (m, 2H, Cy), 1.20-0.95 (m, 6H, Cy), 0.88 (m, 2H, Cy).

^{13}C NMR (126 MHz, CD_2Cl_2, 25°C): δ 165.93 (br, C-B, C_6H_4), 153.41 (br, C-B, Cl_2Ph), 140.48 (*o*-CCl), 137.17 (d, J_{CP} = 17.4 Hz, C_6H_4), 136.98 (d, J_{CP} = 15.7 Hz, C_6H_4), 132.49 (C_6H_4), 129.39 (*m*-C, Cl_2Ph), 126.90 (*p*-CCl), 125.55 (d, J_{CP} = 15.3 Hz, C_6H_4), 120.66 (d, J_{CP} = 104.1 Hz, C-P, C_6H_4), 38.26 (d, J_{CP} = 61.1 Hz, Cy), 30.75 (d, J_{CP} = 90.6 Hz, Cy), 27.14 (d, J_{CP} = 7.6 Hz, Cy), 27.01 (d, J_{CP} = 7.3 Hz, Cy), 26.16 (d, J_{CP} = 2.4 Hz, Cy).

gHSQC (F2/F1, ^{1}H/^{13}C, 500/126 MHz, CD_2Cl_2, 25°C): δ 7.43/137.09, 7.79/136.94, 7.37/132.45, 7.10/129.36, 6.94/129.35, 6.94/126.85, 7.10/126.80, 7.20/125.52, 2.58/38.23, 1.88/31.13, 1.08/31.03, 2.10/30.41, 1.39/30.41, 1.65/27.17, 1.03/27.06, 0.88/27.05, 1.71/27.00, 1.58/26.15, 1.14/26.14

^{31}P NMR (202 MHz, CD_2Cl_2, 25°C): δ 43.30 (d, J_{PH} = 332.0 Hz).

^{11}B NMR (160 MHz, C_6D_6, 25°C): δ 0.67 (s).

Elemental microanalysis: calc. for $C_{30}H_{34}BCl_4OP$, C 60.64% H 5.77% Elem. found: C 59.54 % H 5.98 %

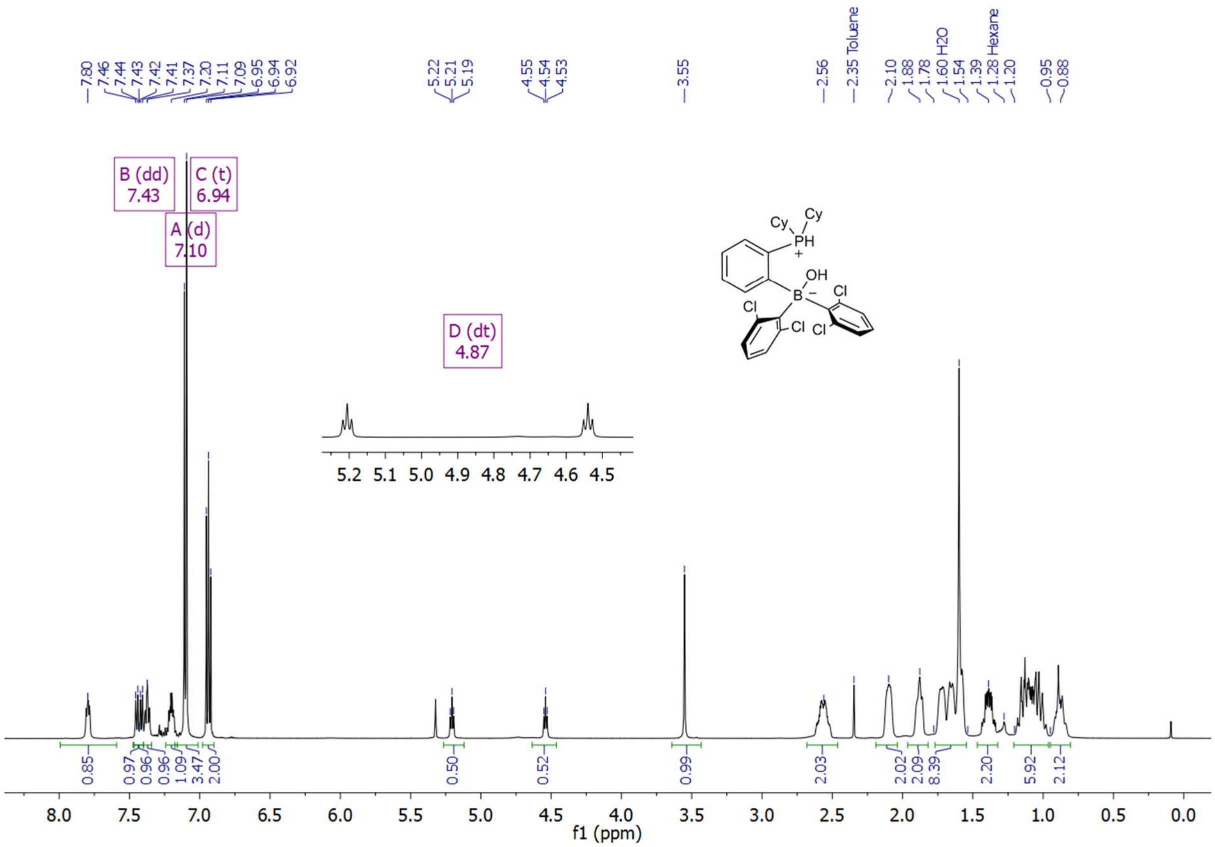

Figure S15. ^{1}H NMR (500 MHz, CD_2Cl_2, 25°C) spectrum of compound **1-H$_2$O**.

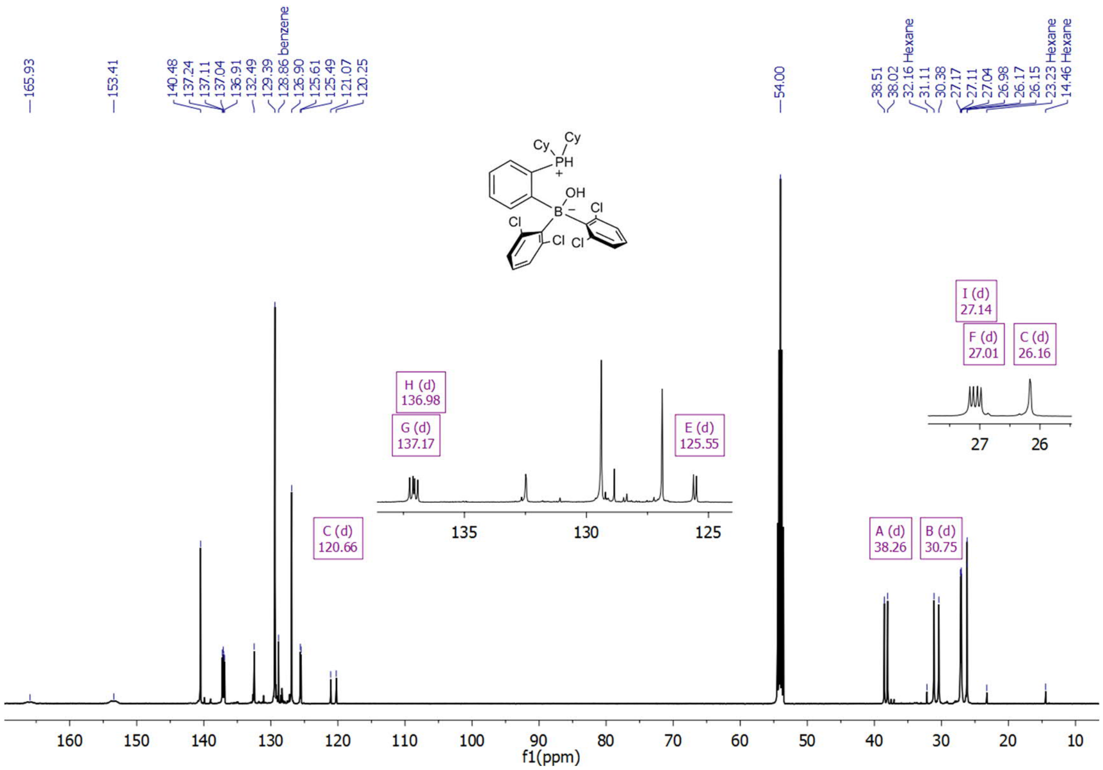

Figure S16. ^{13}C NMR (126 MHz, CD_2Cl_2, 25°C) spectrum of compound **1-H$_2$O**.

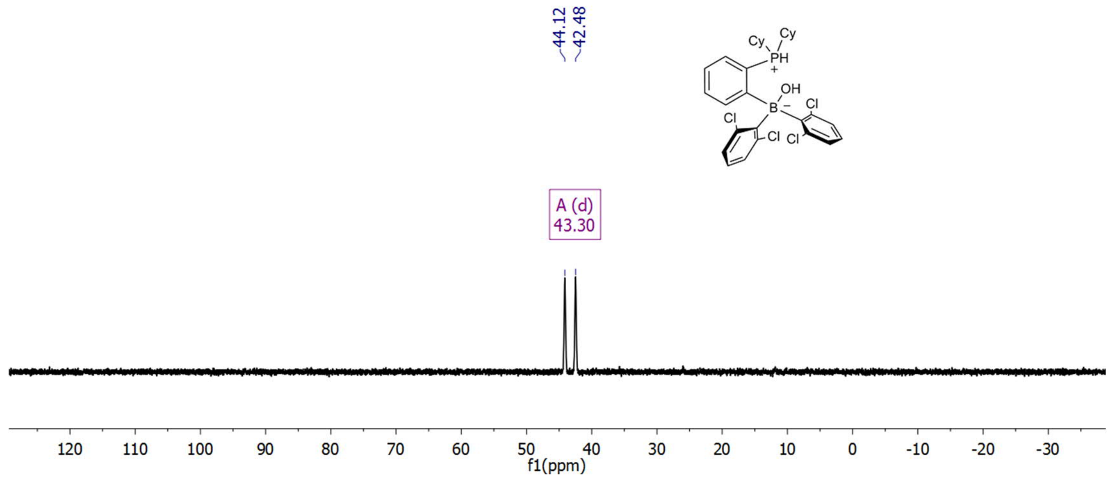

Figure S17. ^{31}P NMR (121 MHz, CD_2Cl_2, 25°C) spectra of compound **1-H_2O**.

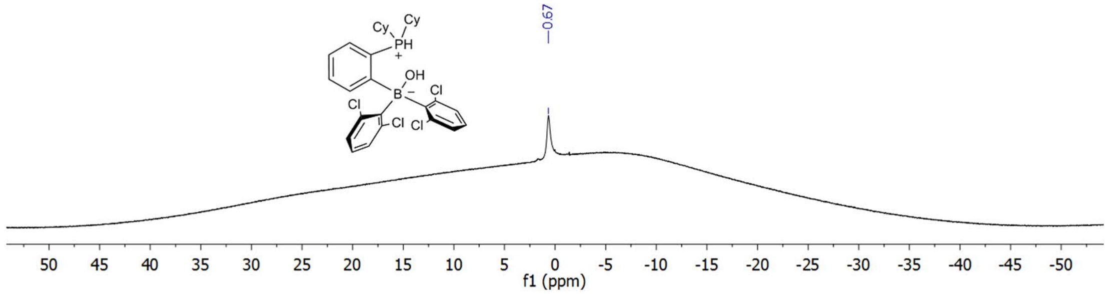

Figure S18. ^{11}B NMR (160 MHz, CD_2Cl_2, 25°C) spectrum of compound **1-H_2O**.

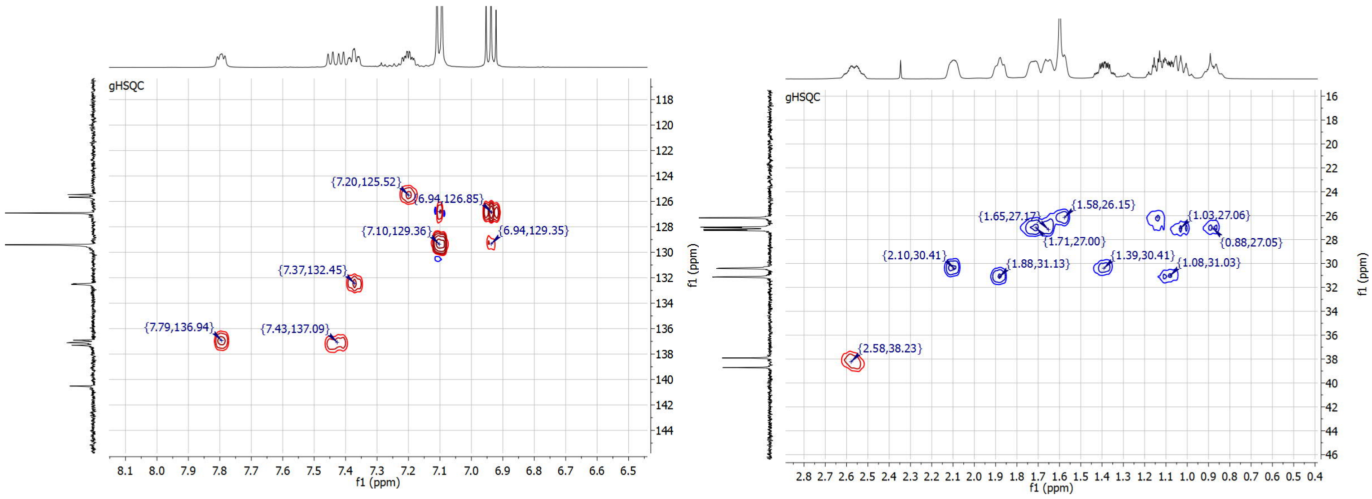

Figure S19. gHSQC NMR (500/126 MHz, CD_2Cl_2, 25°C) spectrum of compound **1** (aromatic and aliphatic regions).

Deuterated water addition to 1 (synthesis of 1-D_2O).

In a glovebox, a gas-tight NMR tube was charged with **1** (11 mg, 18.5 µmol), 8 equivalents of deuterated water (~3 mg), and 0.5 ml of 1:1 CD_3CN:CD_2Cl_2 mixture. Full conversion of **1** to **1-D_2O** was observed according to heteronuclear NMR analysis.

^{1}H NMR (500 MHz, CD_2Cl_2+CD_3CN, 25°C): δ 7.66 (m, 1H, C_6H_4), 7.41 (dd, J_{HP}= 16.9, J_{HH} =7.6 Hz, 1H), 7.27 (m, 1H, C_6H_4), 7.13 (m, 1H, C_6H_4), 7.02 (d, J_{HH} = 7.9 Hz, 4H, $Cl_2C_6H_3$), 6.87 (t, J = 7.9 Hz, 2H, $Cl_2C_6H_3$), 2.50(m, 2H, Cy), 2.02 (brm, 2H, Cy), 1.76 (brm, 2H, Cy), 1.66-1.44 (brm, 6H, Cy), 1.33 (m, 2H, Cy), 1.06 (m, 4H, Cy), 0.94 (m, 2H), 0.80 (m, 2H, Cy).

^{31}P NMR (202 MHz, CD_2Cl_2+CD_3CN, 25°C): δ 42.07 (t, J_{HP} = 52.0 Hz).

^{11}B NMR (160 MHz, CD_2Cl_2+CD_3CN, 25°C): δ 0.54 (s).

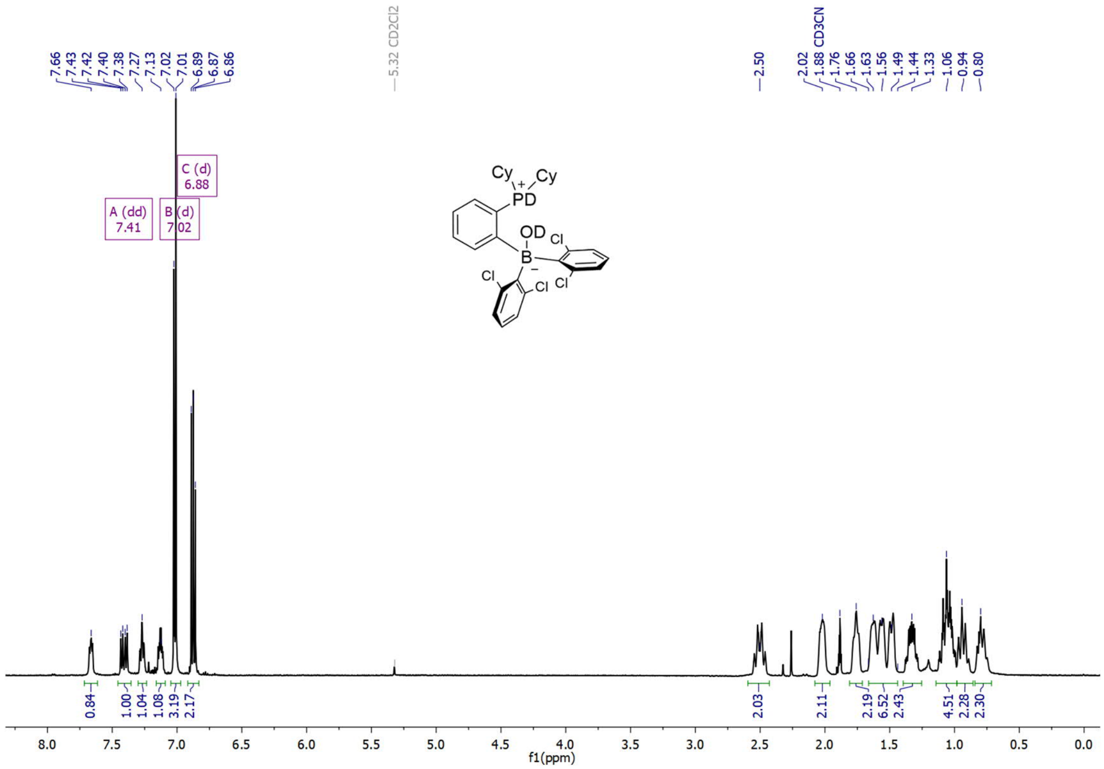

Figure S20. ^{1}H NMR (500 MHz, CD_2Cl_2, 25°C) spectrum of compound **1-D_2O**.

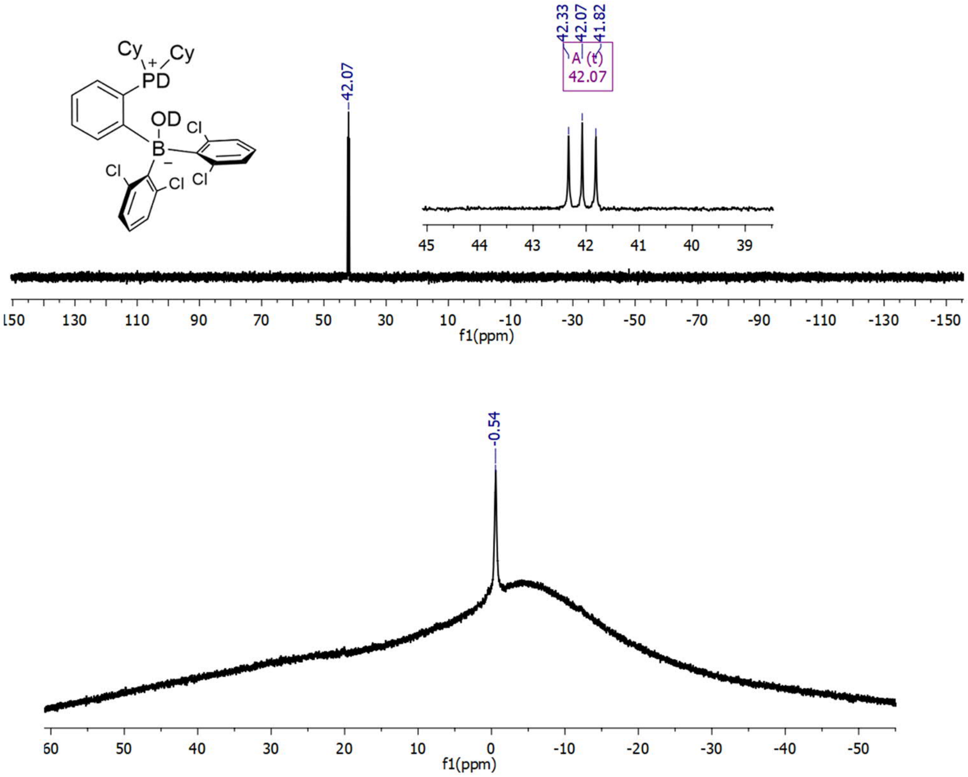

Figure S21. ^{31}P NMR (121 MHz, CD_2Cl_2+CD_3CN, 25°C) (top) and ^{11}B NMR (160 MHz, CD_2Cl_2+CD_3CN, 25°C) (bottom) spectra of compound **1-D₂O**.

Synthesis of 1-O

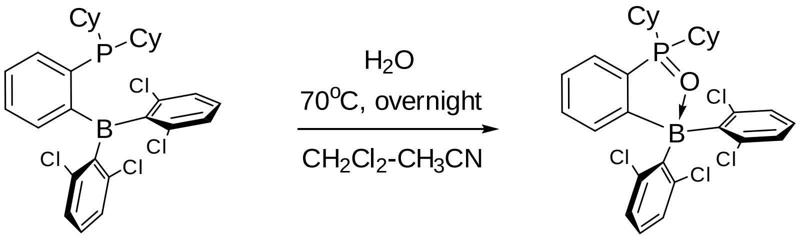

1 **1-O**

In a 25 ml Schlenk tube a solution of **1** (560 mg, 0.43 mmol, 1eq.) and H_2O (16 mg, 0.88 mmol, 2 eq.) in 7 ml of the mixture 1:1 CH_3CN:CH_2Cl_2 was heated at 70°C overnight. The solvent was evaporated to dryness to give a spectroscopically pure product. Subsequent recrystallized of the residue from 5 ml of 1:9 CH_2Cl_2:CH_3CN mixture gave **1-O** as colorless crystals (366 mg, 64 %)

^{1}H NMR (500 MHz, CD_2Cl_2, 25°C): 8.07 (d, J_{HH} = 7.7 Hz, 1H), 7.47 (t, J_{HH} = 7.4 Hz, 1H), 7.40 (tm, J_{HH} = 7.5 Hz, 1H), 7.30 (m, 1H), 7.09 (d, J_{HH} = 7.9 Hz, 4H), 6.93 (t, J_{HH} = 7.8 Hz, 2H), 2.20 (m, 2H), 1.99 (brm, 2H), 1.89 (brm, 2H), 1.74 (brm, 4H), 1.65 (brm, 2H), 1.36 (m, 4H), 1.24-0.98 (m, 6H).

^{13}C NMR (126 MHz, CD_2Cl_2, 25°C): δ 165.27 (br, C-B, C_6H_4), 149.72 (br, C-B, Cl_2Ph), 139.88 (*o*-CCl), 134.98 (d, J_{CP} = 14.2 Hz, C_6H_4), 131.79 (d, J_{CP} = 3.2 Hz, C_6H_4), 129.22 (*m*-C, Cl_2Ph), 127.89 (d, J_{CP} = 15.2 Hz, C_6H_4), 127.83 (brs, C_6H_4), 127.23 (*p*-CCl), 126.82 (d, J_{CP} = 11.5 Hz, C_6H_4), 37.29 (d, J_{CP} = 58.6 Hz, Cy, 2C), 26.85 (m, 27.12-26.78, Cy, 6C), 26.70 (m, J_{CP} = 13.2 Hz, Cy, 4C).

^{31}P NMR (202 MHz, CD_2Cl_2, 25°C): δ 83.67

^{11}B NMR (160 MHz, C_6D_6, 25°C): δ 7.46

Elemental microanalysis: calc. for $C_{30}H_{32}BCl_4OP$, C 60.85% H 5.45%

Elem. found: C 60.90 % H 5.08 %

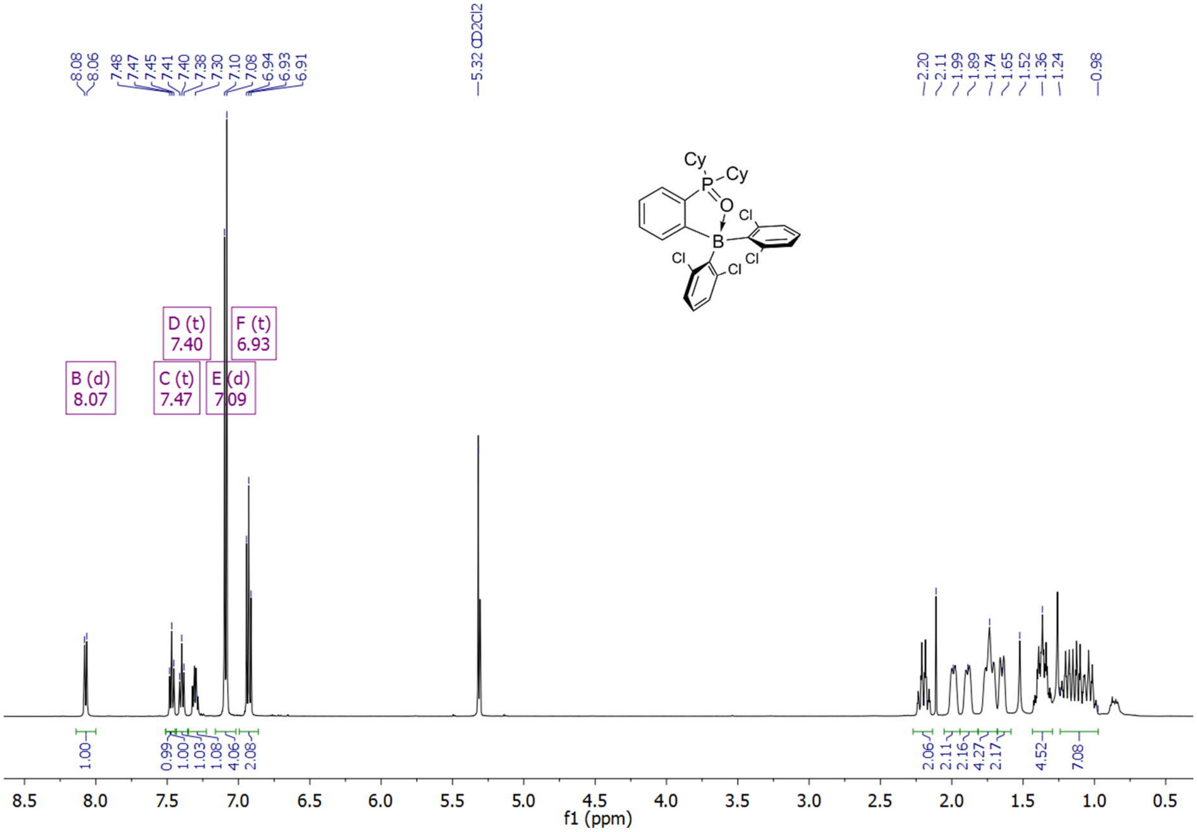

Figure S22. ^{1}H NMR (500 MHz, CD_2Cl_2, 25°C) spectrum of compound **1-O**.

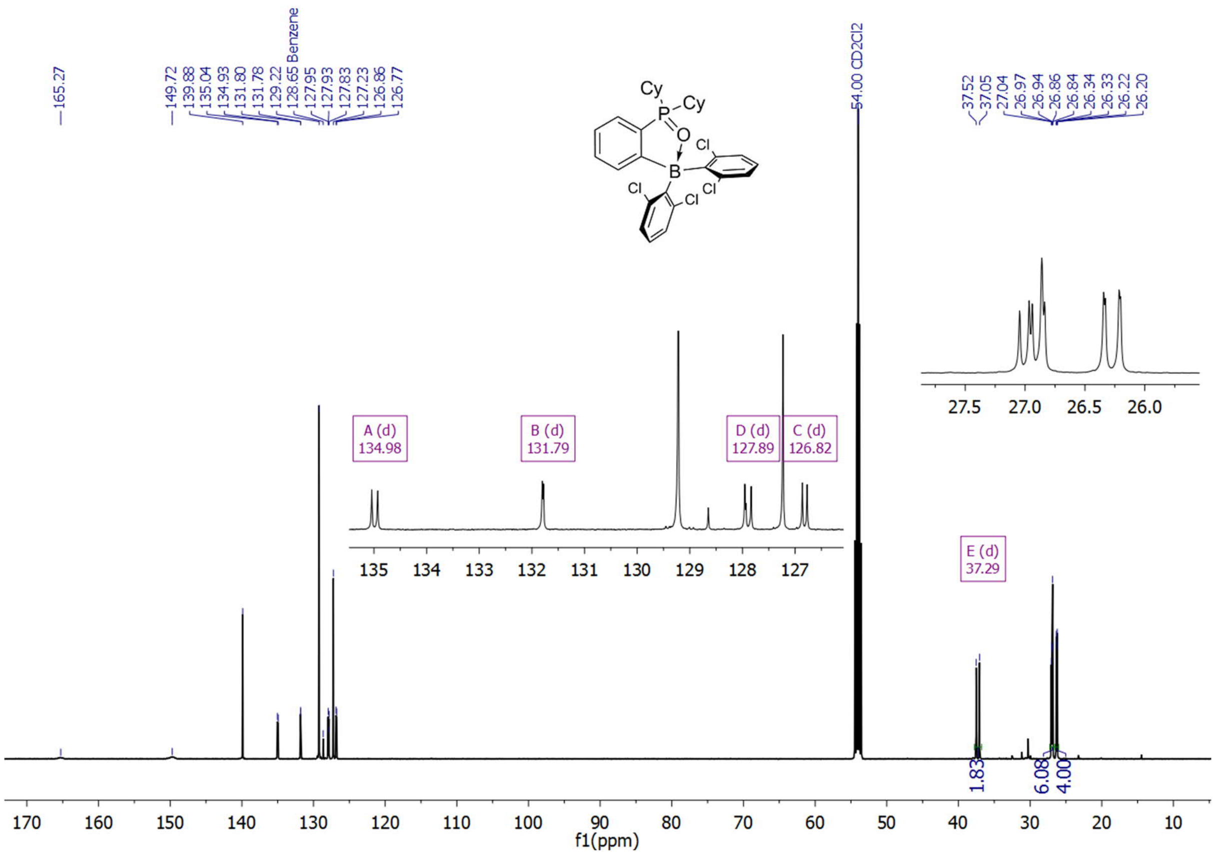

Figure S23. ^{13}C NMR (126 MHz, CD_2Cl_2, 25°C) spectrum of compound **1-O**.

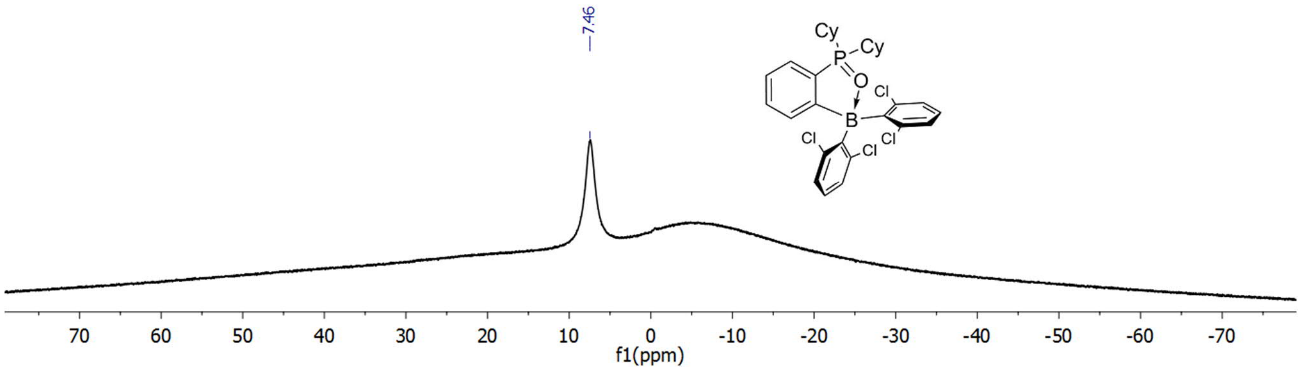

Figure S24. ^{11}B NMR (160 MHz, CD_2Cl_2, 25°C) spectrum of compound **1-O**.

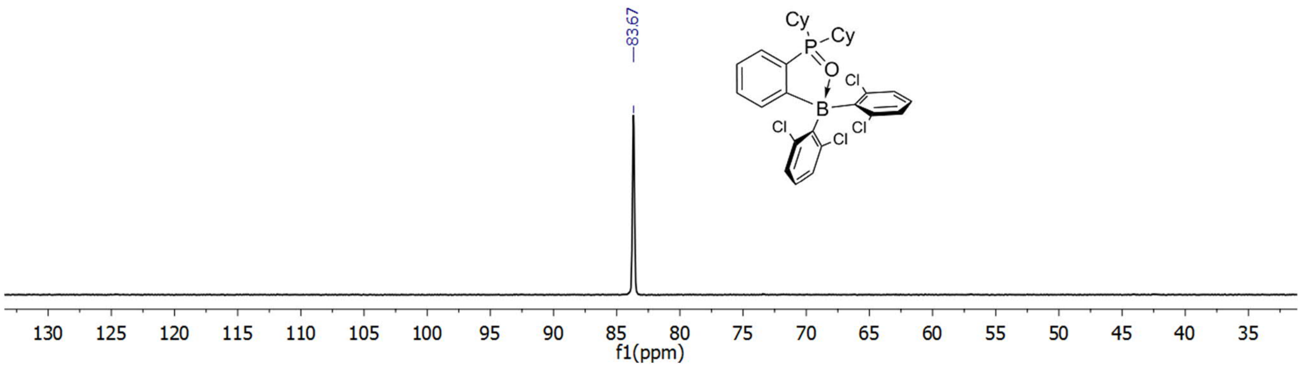

Figure S25. ^{31}P NMR (121 MHz, CD_2Cl_2, 25°C) spectrum of compound **1-O**.

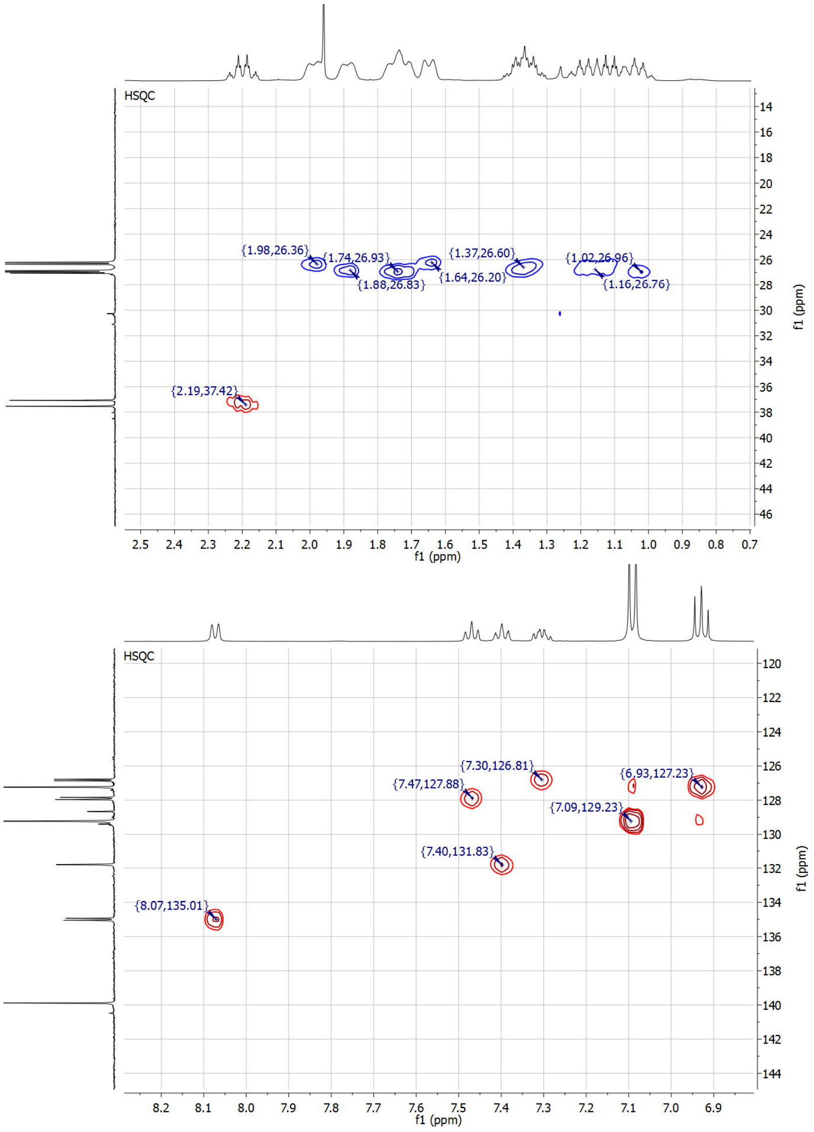

Figure S26. gHSQC NMR (500/126 MHz, CD_2Cl_2, 25°C)(top) spectrum of compound **1-O** (aliphatic and aromatic regions)(bottom).

Reaction of **1** with H_2/D_2

$$\mathbf{1} + H_2/D_2\ (5\text{bars}) \xrightarrow[\text{12 hours}]{\text{RT}} \mathbf{1\text{-}D_2} + HD$$

A heavy wall J. Young NMR tube, containing a solution of **1** (5 mg, 8.7 μmol) in 0.3ml of C_6D_6 was pressurized with 5 bars of D_2.^{1}H and ^{11}B NMR of this sample showed complete conversion of **1** into zwitterionic adduct **1-D₂**. Then the sample was pressurized with 5 bars of H_2, and ^{1}H and ^{11}B NMR were recorded again to ensure the presence of H_2. After 12 hours at room temperature NMR analysis revealed the formation of HD, while the sample still contains mostly **1-D₂**. The ratio of H_2 to HD peaks 1:6 indicated 75% conversion of the H_2/D_2 mixture into HD.

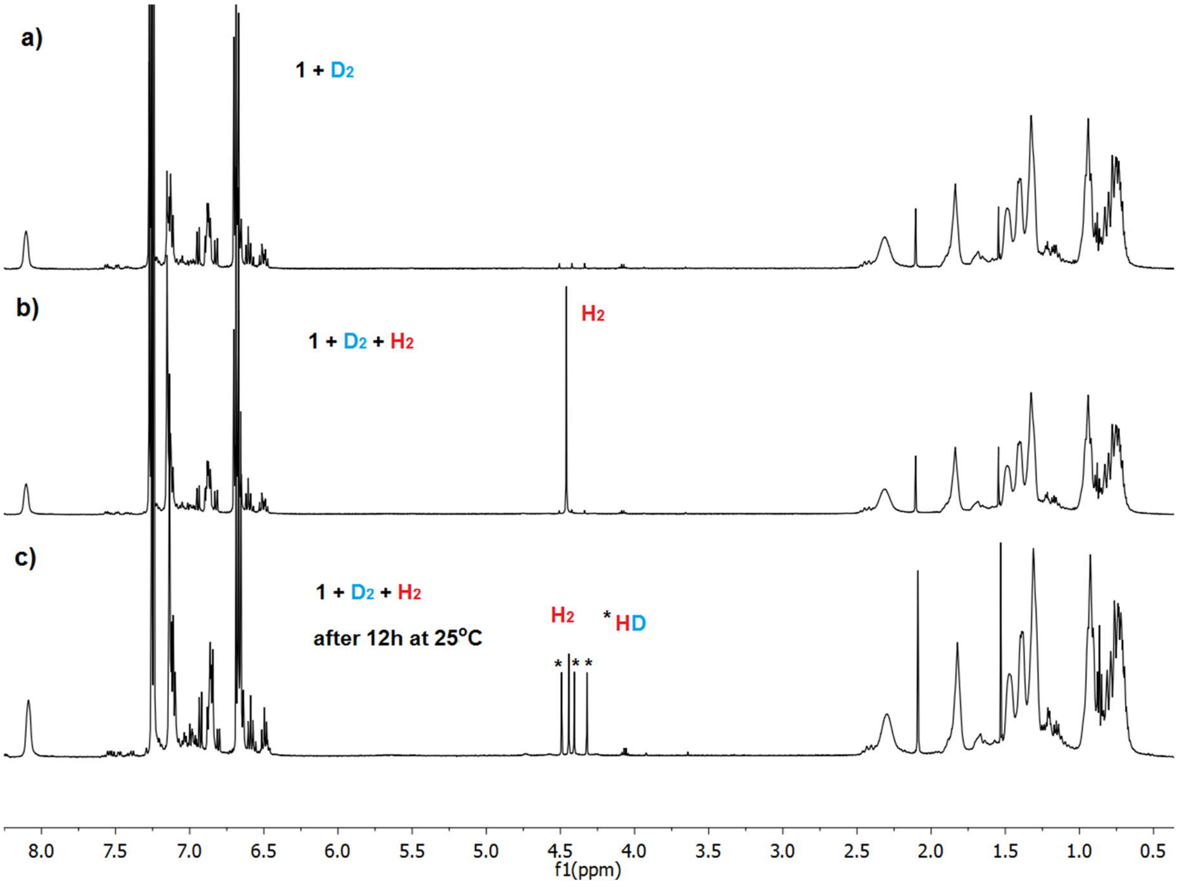

Figure S27. Stacked NMR spectra of (a) **1** under D_2, **1** under the mixture D_2/H_2 mixture(b) and 1 under D_2/H_2 mixture after 12 hours at 25°C.

NMR Monitoring of PCy_3/ H_2O /$B(2,6\text{-}Cl_2C_6H_3)_3$ in the solution.

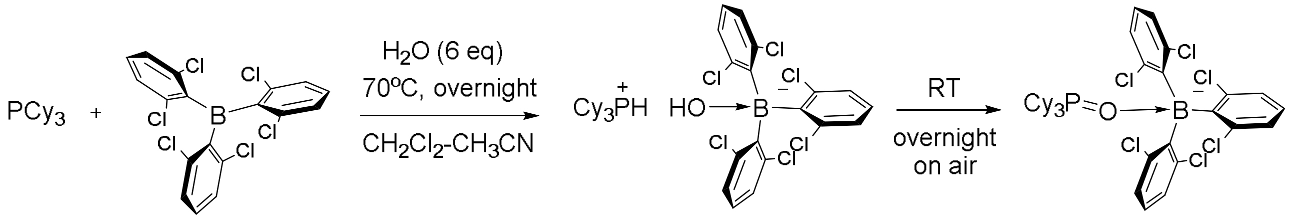

In a glovebox, a gas-tight NMR tube was charged with $B(2,6\text{-}Cl_2C_6H_3)_3$ (10 mg, 21 mmol), PCy_3(6 mg, 21 mmol) and 0.6 mL of a deoxygenated mixture of 1:1 CH_3CN:CH_2Cl_2 containing 6 equivalents of water (2.3 mg, 128 mmol). ^1H, ^{11}B, and ^{31}P were recorded at room temperature and suggested the formation of anionic $[HPCy_3]^+[B(2,6\text{-}Cl_2C_6H_3)_3OH]^-$ species (Fig. S28-30). ^1H NMR spectra of the solution featured a wide doublet at 5.90 ppm (d, J_{PH} = 464.2 Hz) suggesting a direct P-H bond. Boron signal appeared at 43.17 ppm as a singlet which is far downfield of the range expected for tetracoordinate anionic boron. We hypothesized that this may be an exchange peak between $[B(2,6\text{-}Cl_2C_6H_3)_3OH]^-$ and free $B(2,6\text{-}Cl_2C_6H_3)_3$ presented in the mixture.

^{1}H NMR (500 MHz, CD_2Cl_2+CD_3CN, 25°C) δ 7.21 (s, 9H), 5.90 (dq, J = 464.2, 4.1 Hz, 1H, P-H), 2.45 (m, 3H), 1.91 (brm, 6H), 1.79 (brm, 6H), 1.67 (brm, 3H), 1.51 (m, 6H), 1.33 (m, 6H), 1.25 (m, 3H).

^{31}P NMR (202 MHz, CD_2Cl_2, 25°C): δ 28.36 (d, J_{PH} = 463.7 Hz)

^{11}B NMR (160 MHz, C_6D_6, 25°C): δ 43.17

The resulting colorless solution was heated at 70°C inside the NMR spectrometer for 9 hours and periodically monitored by ^1H NMR (Fig. S31). No visible changes, except the appearance of a trace peak at 4.53 ppm followed by its gradual disappearance, were detected. Proton coupled and decoupled ^{31}P NMR recorded after heating also did not show notable changes (Fig. S33a,b). The same sample was kept open on air overnight to obtain the oxidized form of the complex, namely $Cy_3PO\rightarrow B(2,6\text{-}Cl_2C_6H_3)_3$, for reference (Fig. S32, S33c,d). ^1H, ^{31}P NMR spectra confirmed nearly complete oxidation, and their comparison with ^1H, ^{31}P spectra of PCy_3-H_2O-$B(2,6\text{-}Cl_2C_6H_3)_3$ after heating confirmed the absence of an oxidized form of the complex in the latter.

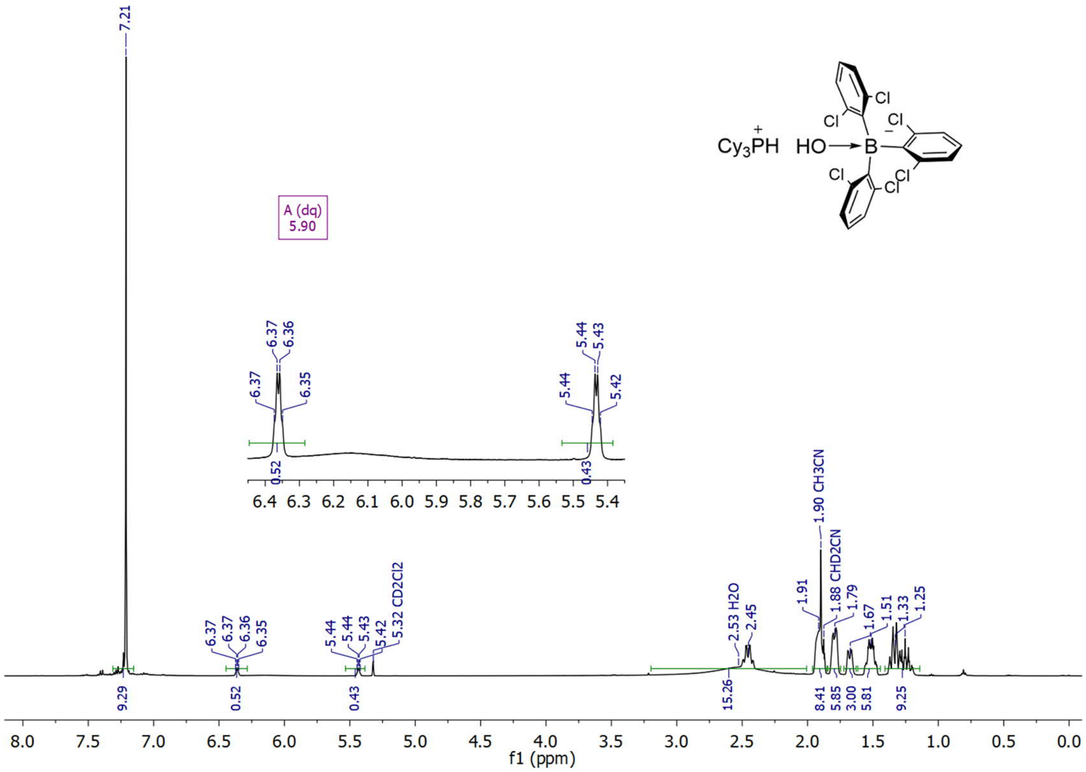

Figure S28. ^{1}H NMR (500 MHz, CD_2Cl_2, 25°C) spectrum of PCy_3-H_2O-B(2,6-$Cl_2C_6H_3$)$_3$.

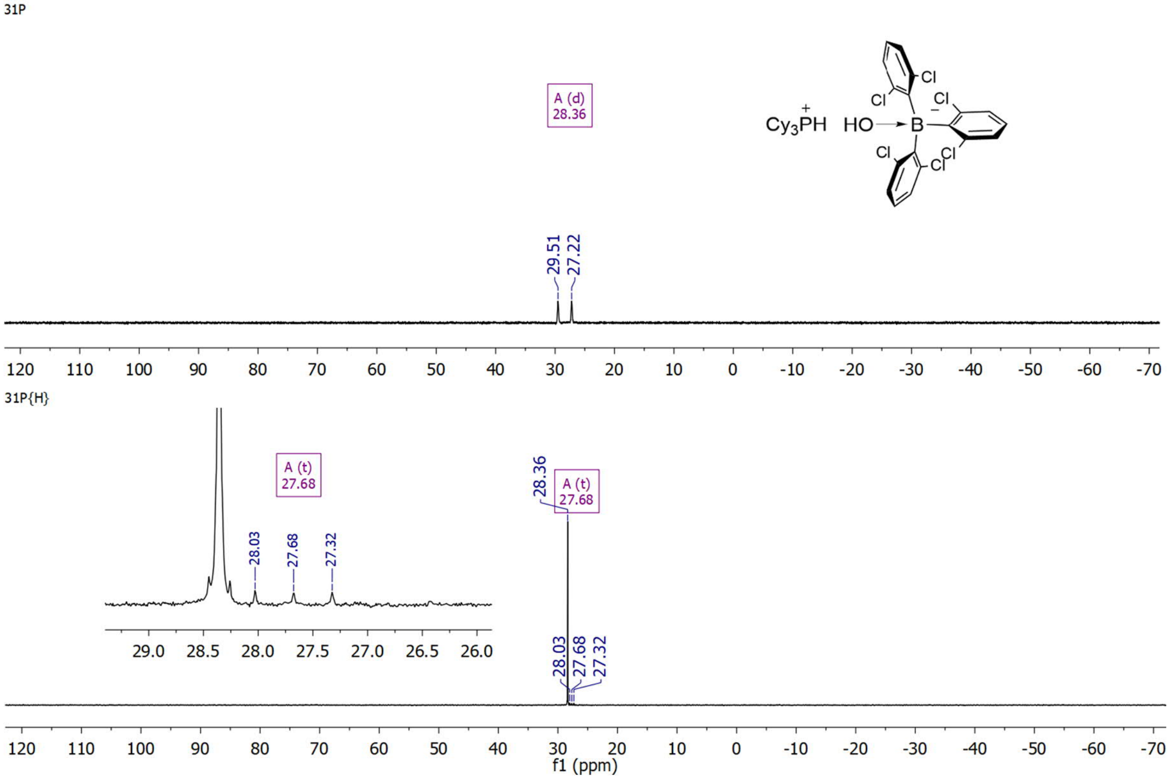

Figure S29. Proton-coupled (top) and proton-decoupled (bottom) ^{31}P NMR (121 MHz, CD_2Cl_2+CD_3CN, 25°C) spectra of PCy_3-H_2O-B(2,6-$Cl_2C_6H_3$)$_3$.

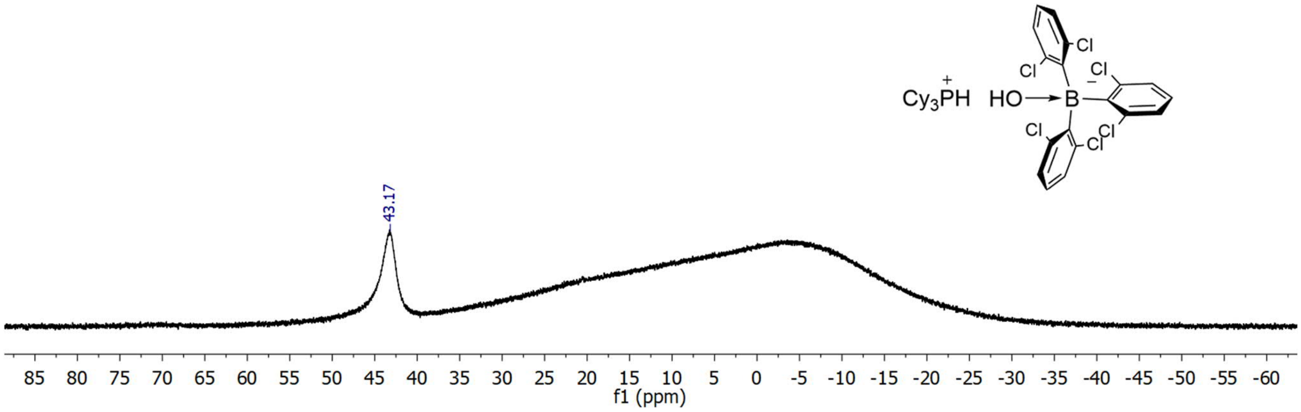

Figure S30. ^{11}B NMR (160 MHz, CD_2Cl_2+CD_3CN, 25°C) spectrum of PCy_3-H_2O-B(2,6-$Cl_2C_6H_3$)$_3$.

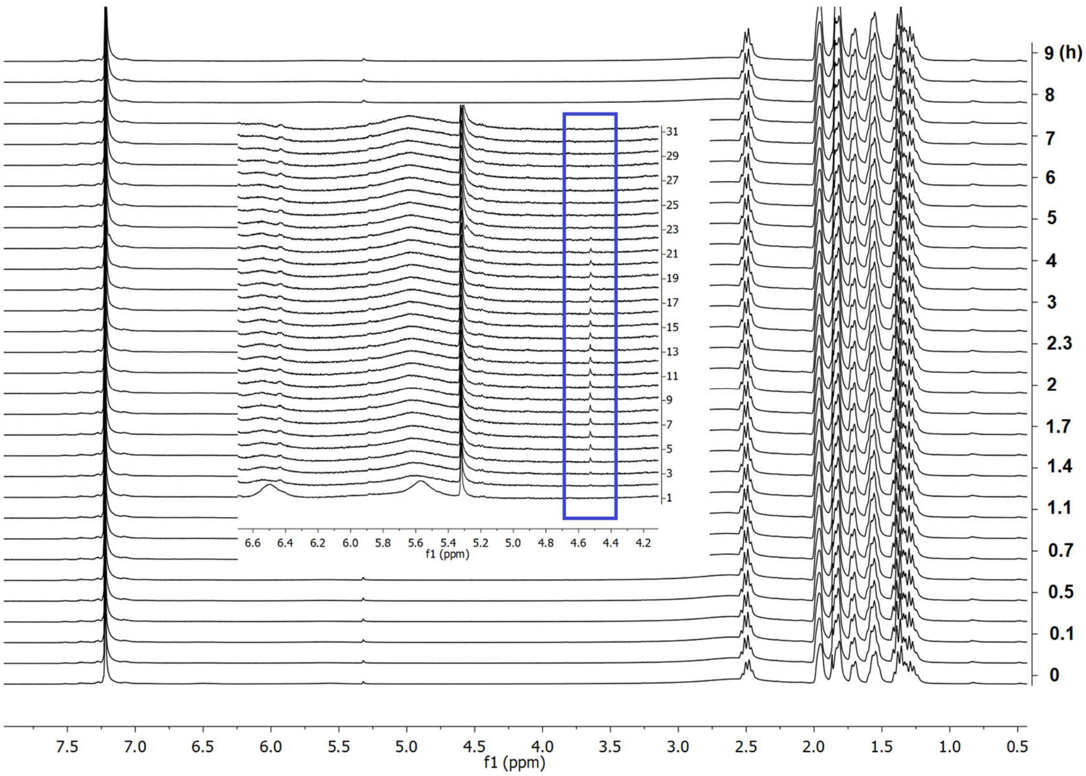

Figure S31. *In situ* ^{1}H-NMR spectra of PCy_3/H_2O/B(2,6-$Cl_2C_6H_3$)$_3$ 1:6:1 in 1:1 CH_3CN:CH_2Cl_2 monitored at 70°C during 9 hours.

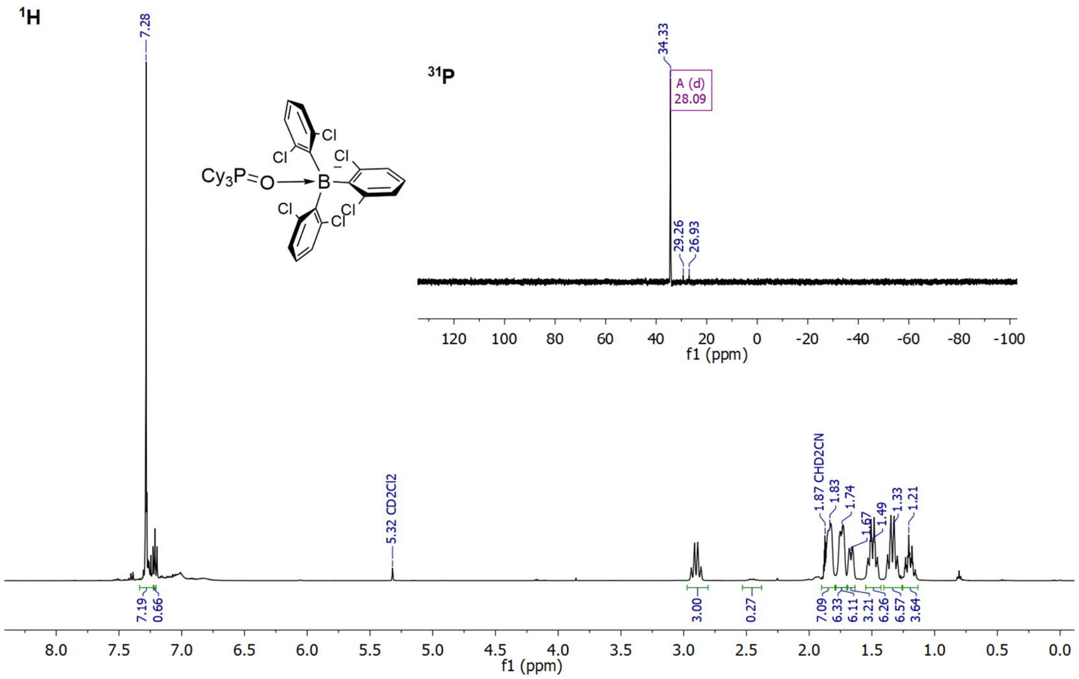

Figure S32. ^{1}H (500 MHz, $CH_3CN+CH_2Cl_2$, 25°C) and ^{31}P NMR (121 MHz, $CH_3CN+CH_2Cl_2$, 25°C) spectra of $Cy_3PO{\rightarrow}B(2,6\text{-}Cl_2C_6H_3)_3$.

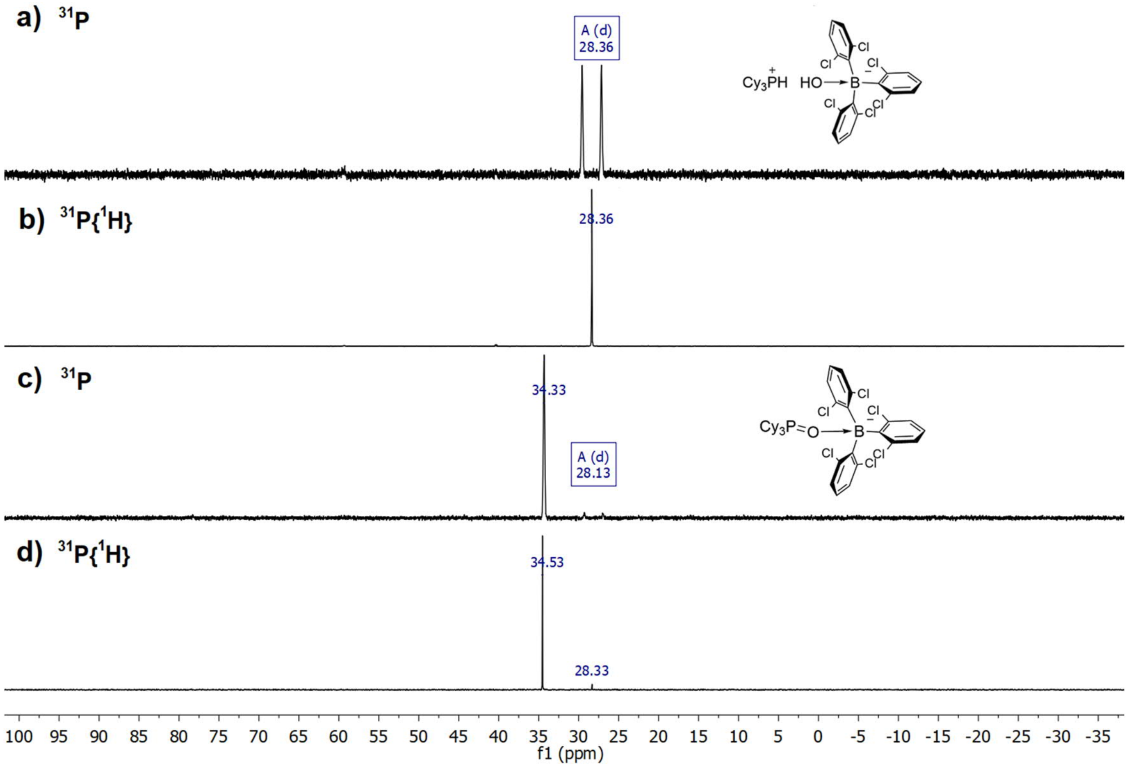

Figure S33. ^{31}P NMR (121 MHz, 1:1 $CH_3CN:CH_2Cl_2$, 25°C) spectra of $PCy_3/H_2O/B(2,6\text{-}Cl_2C_6H_3)_3$ after heating at 70°C during 9.5 hours (**(a)** proton coupled, **(b)** proton decoupled), and of $Cy_3PO{\rightarrow}B(2,6\text{-}Cl_2C_6H_3)_3$ in 1:1 $CH_3CN:CH_2Cl_2$ (**(c)** proton coupled, **(d)** proton decoupled).

Hydrogen activation by **1** in presence of water.

In a glovebox, 62 mg of H_2O was dissolved in 20 ml of dry and deoxygenated mixture 1:1 CH_3CN:CH_2Cl_2 to prepare moist solvent (0.12 M). The water content of moist solvent was verified by Karl Fischer titrations. In a glovebox, in a vial, **1** (14mg, 0.024 mmol) was dissolved in 1.2 ml of moist solvent (2.6 mg H_2O, 0.144mmol). ^{1}H NMR of this initial solution was then recorded and showed complete conversion of **1** to **1-H$_2$O** and the presence of an excess of water (Fig. S34). Equal portions of this solution (0.3 ml) were transferred into two heavy wall J. Young NMR tubes. These samples were treated as follows: **Sample 1** was heated on an oil bath at 80°C (blank); **Sample 2** was pressurized with 10 bars of H_2 and placed on an oil bath at 80°C. Both samples were taken from oil bath after 1 hour and analyzed by ^{1}H, ^{31}P, and ^{11}B NMR. Then they were placed back on an oil bath and heated at 80°C for additional 2 hours, after which ^{1}H, ^{31}P and ^{11}B NMR were recorded again. Transformations of **1** in **Sample 1** and **Sample 2** are reflected in Fig. 35. The species and their ratios are identified based on NMR data represented in Fig. 35-37)

NMR of initial solution

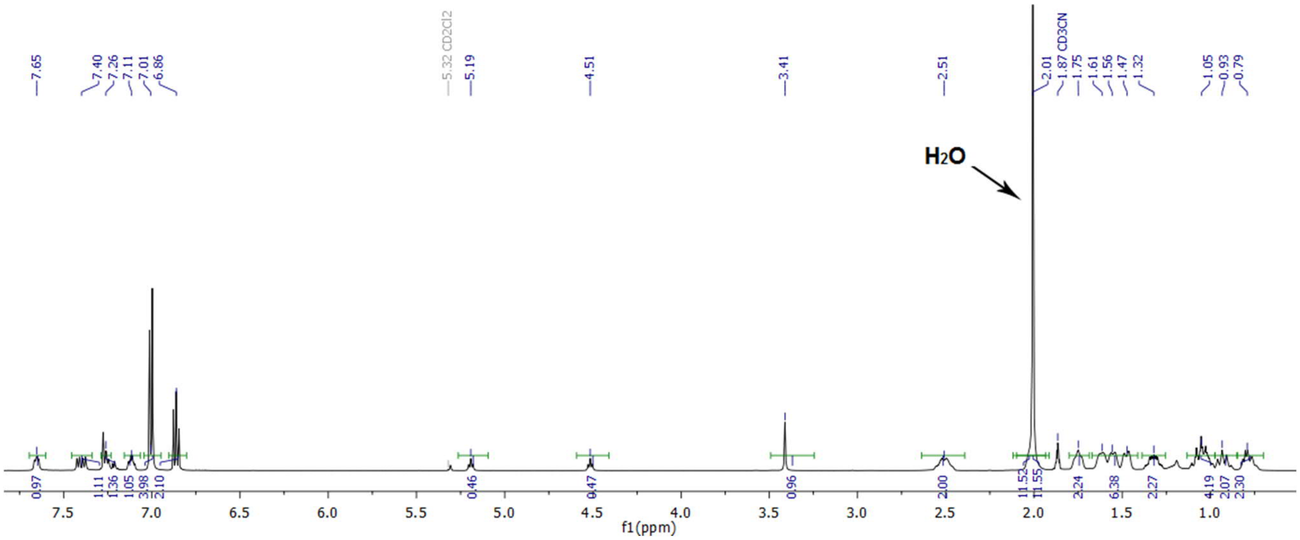

Figure S34. ^{1}H NMR of the initial solution of **1-H$_2$O** in presence of 5 eq. of H_2O prepared by dissolution of **1** in moist solvent.

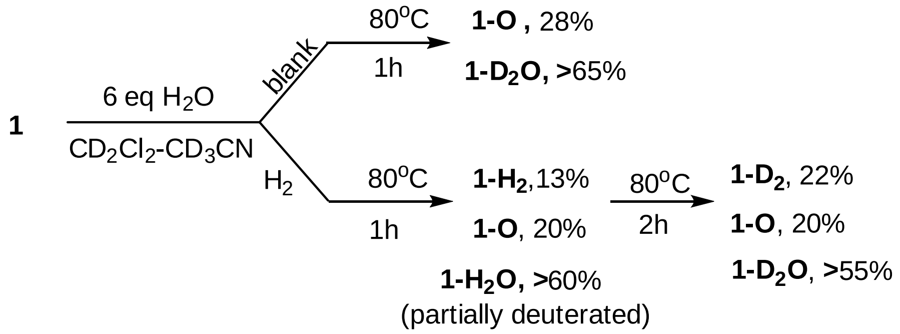

Figure S35. Transformation of **1** in moist solvent (6 eq. of H_2O in 1:1 CD_2Cl_2:CD_3CN) with and without H_2 (10 bars) pressure. The species and their ratios were identified by ^1H, ^{11}B, and ^{31}P NMR spectroscopy.

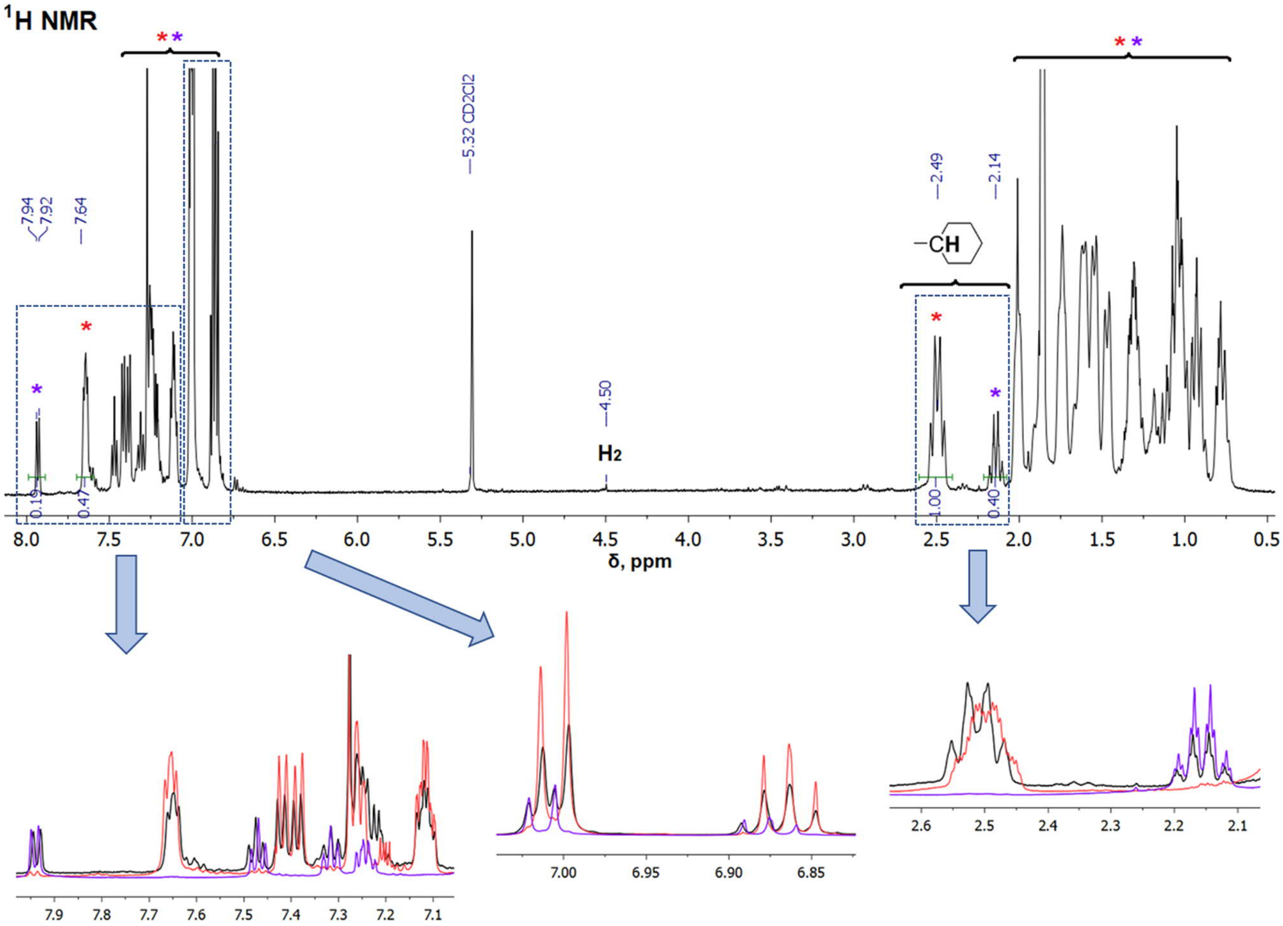

Figure S36. ^1H NMR spectra of **Sample 1** (blank) after heating for 1 hour at 80°C. Signals corresponding to **1-D₂O** (*) and **1-O** (*) are marked with asterisks. c) Selected zooms of the spectrum (black) are superimposed with reference spectra of **1-H₂O** (red), and **1-O** (violet).

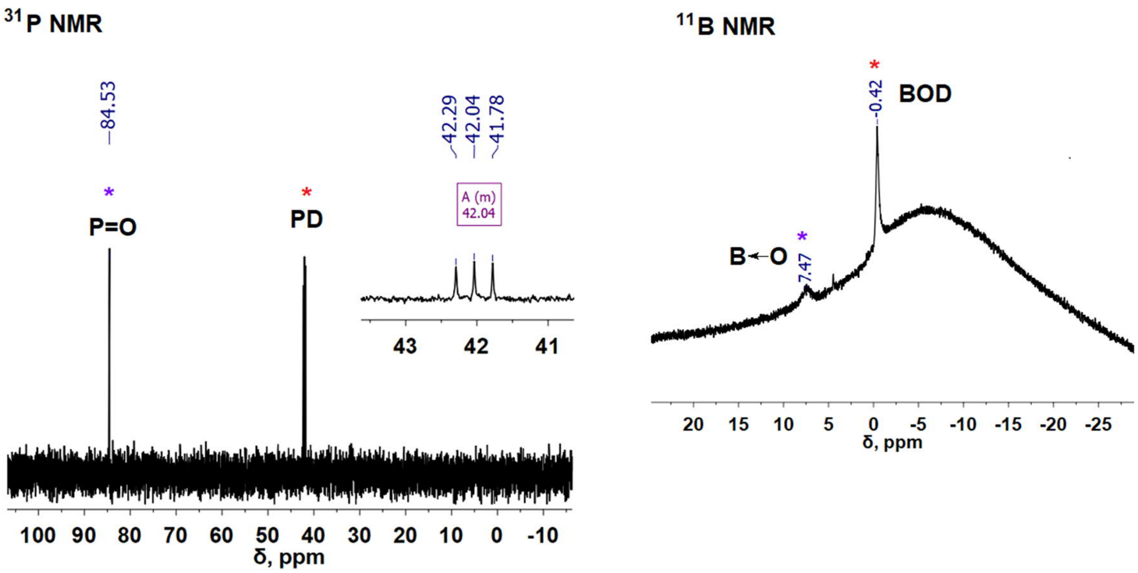

Figure S37. ^{31}P and ^{11}B NMR spectra of **Sample 1** (blank) after heating for 1 hour at 80°C. Signals corresponding to **1-D$_2$O** (*) and **1-O** (*) are marked with asterisks.

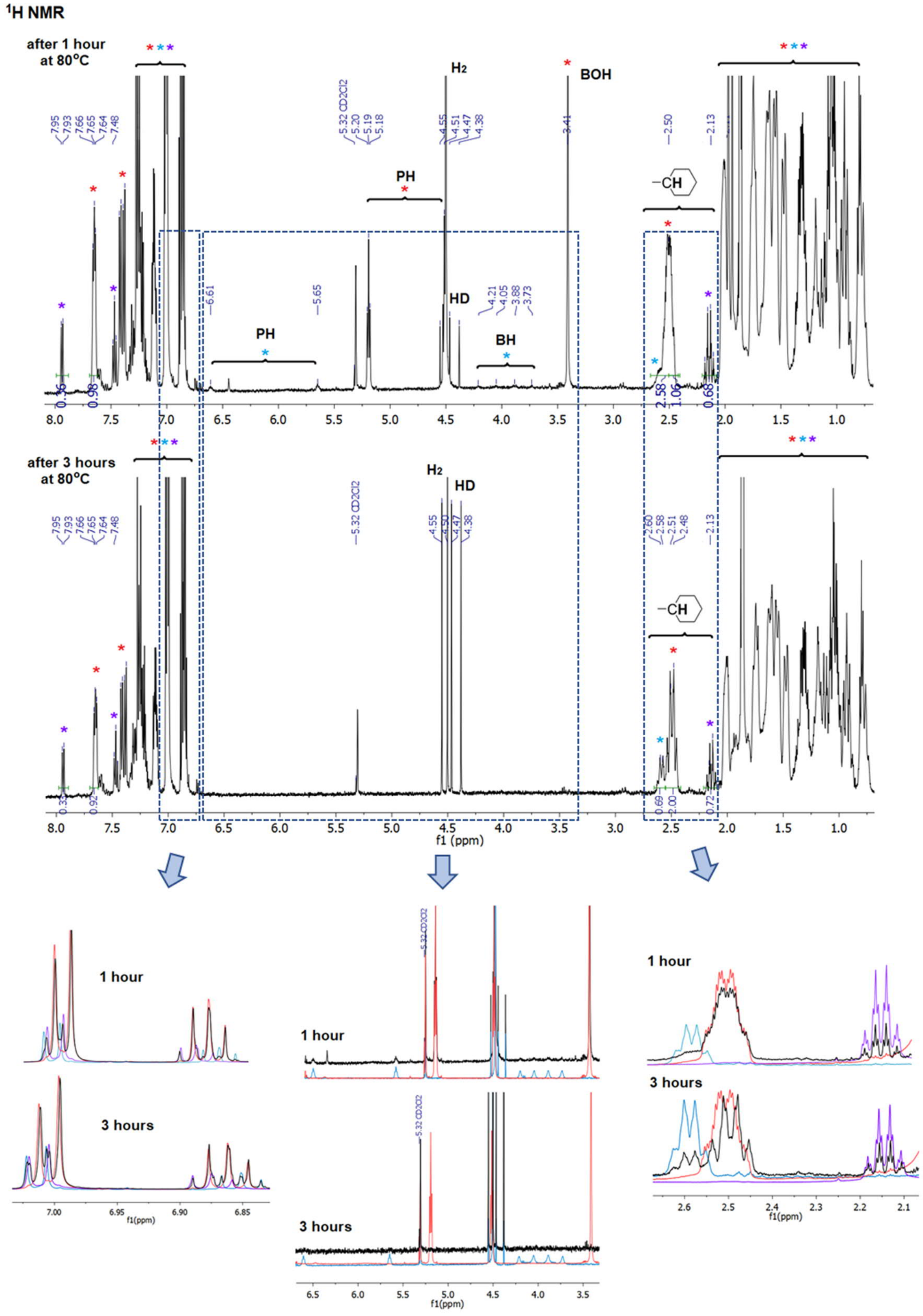

Figure S38. ^{1}H NMR spectra of **Sample 2** after 1 hour of heating at 80°C and after additional 2 hours (3 hours overall) of heating at 80°C. Signals belonging to **1-H$_2$O** (*), **1-H$_2$** (*), and **1-O** (*) are marked with asterisks.

Selected zooms of the spectra (black) are superimposed with reference spectra of **1-H_2O** (red), **1-O** (violet), and **1-H_2** (blue).

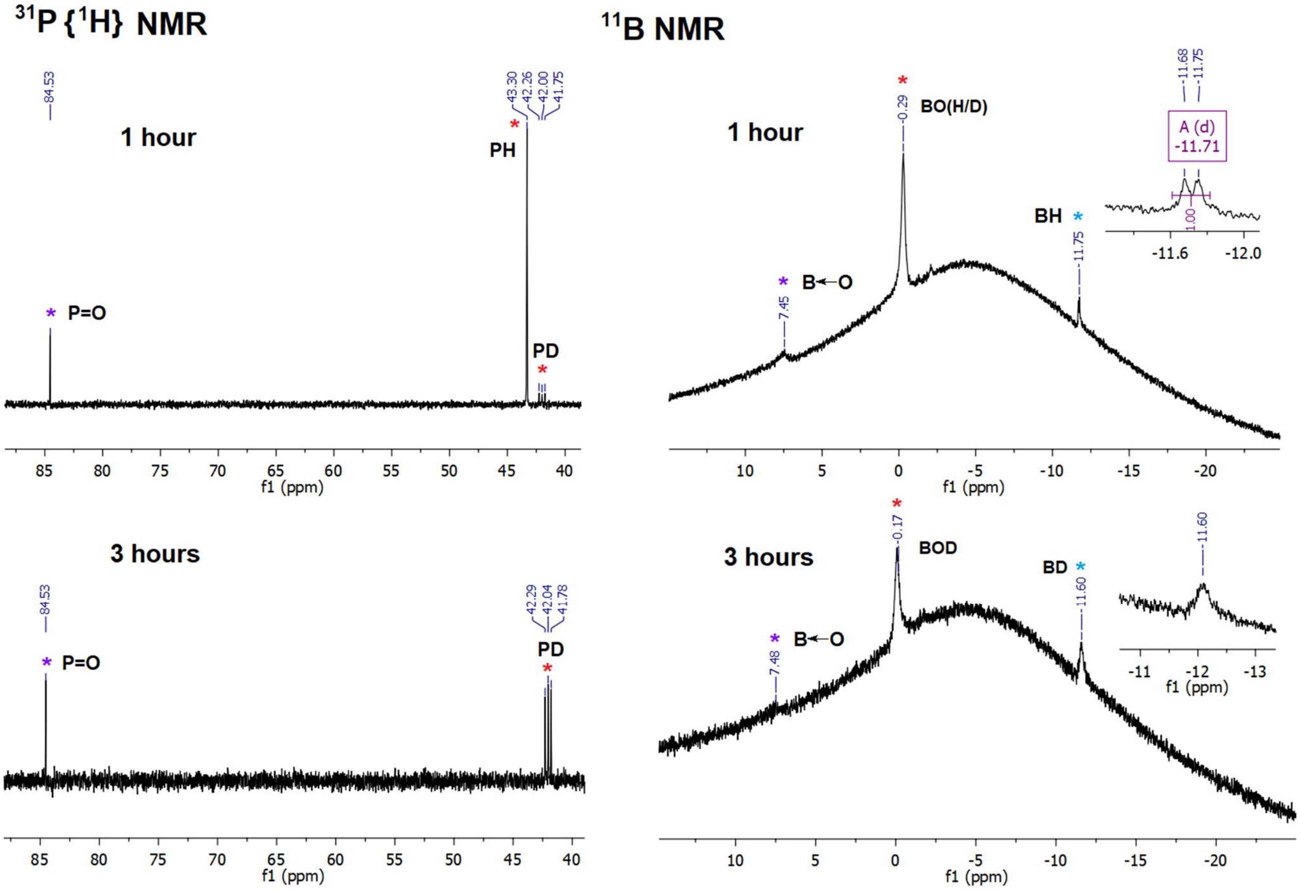

Figure S39. ^{31}P and ^{11}B NMR spectra of **Sample 1** (blank) after 1 hour of heating at 80°C and after additional 2 hours (3 hours overall) of heating at 80°C. Signals corresponding to **1-H_2O** (*), **1-H_2** (*), and **1-O** (*) are marked with asterisks.

Two-chamber experiment

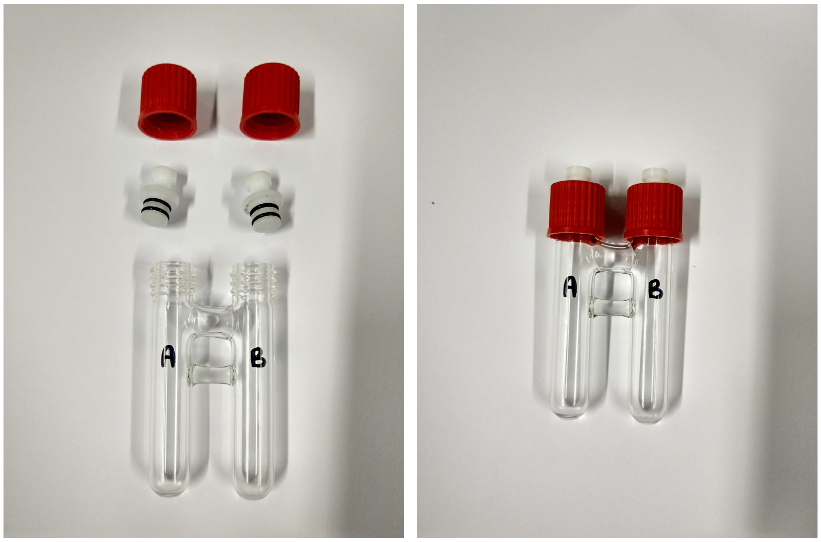

Figure S40. The two-chamber reactor used in the experiment.

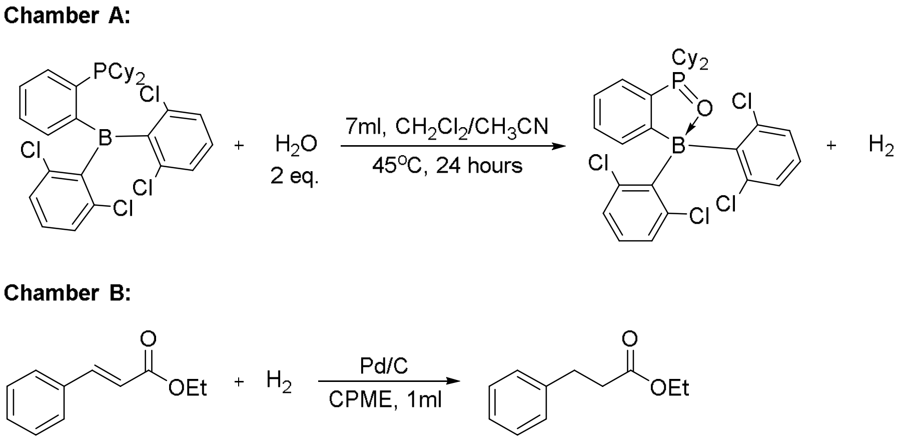

Chamber A: In an argon-filled glovebox to chamber A of a two-chamber system was added **1** (250 mg, 0.43 mmol), H_2O (16 mg, 0.87 mmol) was added as the last thing before immediately closing the last chamber in the two-chamber system with a screw-cap fitted with an H-cap. **Chamber B:** In an argon-filled glovebox to chamber B of a two-chamber system was added ethyl cinnamate (76 mg, 0.43 mmol), Pd/C (10 wt%), and CPME (1.0 mL). The chamber was sealed with a screw-cap fitted with a gas-tight H-cap. The two-chamber system was removed from the glovebox and stirred at room temperature for 18 h. The mixture from chamber B was filtered through a Celite pad and washed with CH_2Cl_2 to obtain the desired product after concentration in vacuo (94% conversion according to crude ^1H NMR, Fig. S41).

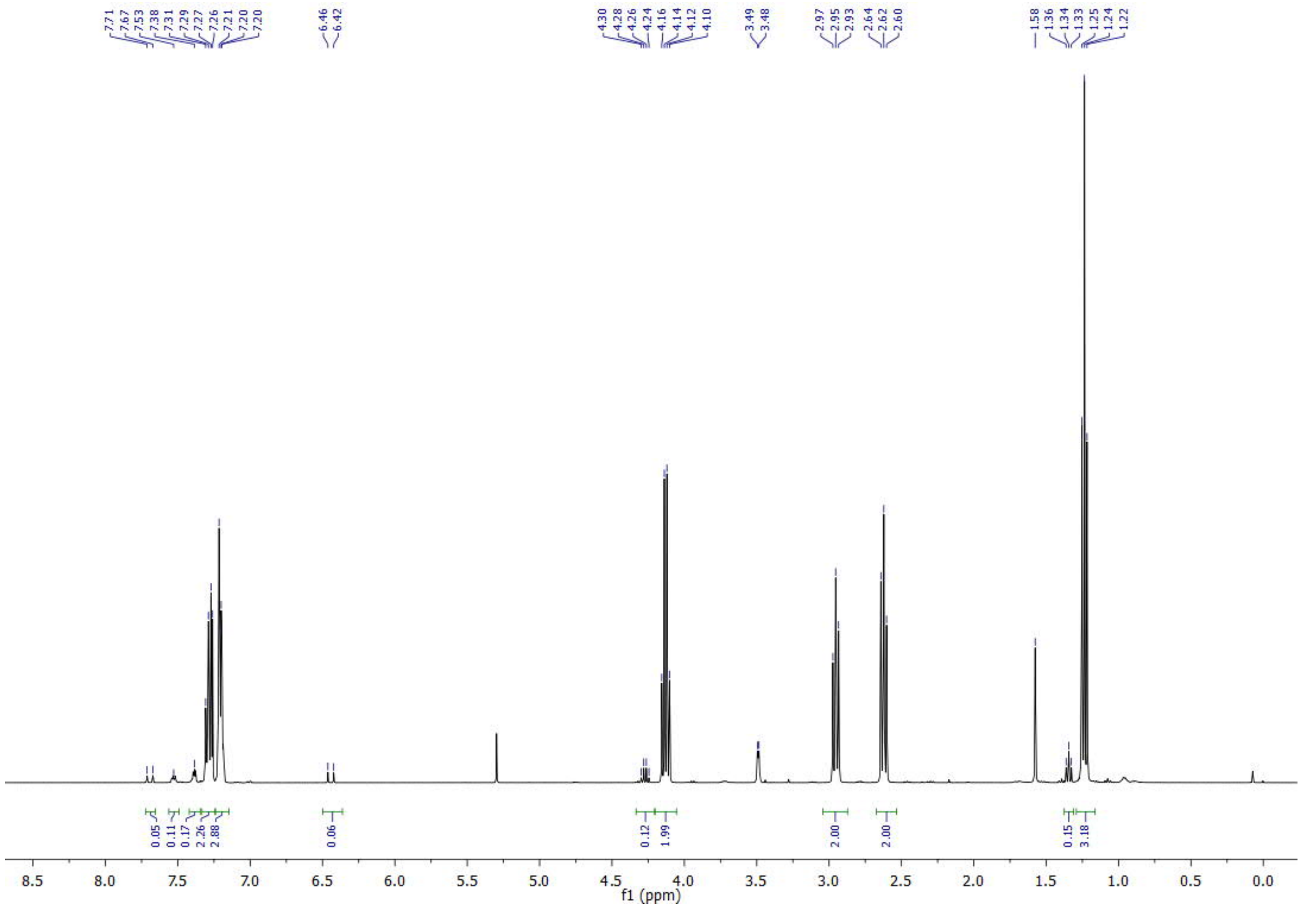

Figure S41. ^{1}H NMR spectra of the crude reaction mixture from the Chamber B after reduction (ethyl 3-phenylpropanoate-major compound, ethyl cinnamate – minor compound)

Crystal Structure Determinations of **1**, **1**-H_2O and **1**-O

The single-crystal X-ray diffraction studies were carried out on a Bruker D8 Venture diffractometer with Photon100 or PhotonII detector at 123(2) K using Cu-Kα radiation (λ = 1.54178 Å). Direct Methods (SHELXS-97)[3] or dual space methods (SHELXT for **5a**)[4] were used for structure solution and refinement was carried out using SHELXL-2014 (full-matrix least-squares on F^2).[5] Hydrogen atoms were localized by difference electron density determination and refined using a riding model (H(O) free). Semi-empirical absorption corrections were applied. For **1** an extinction correction was applied. In 1-O disordered acetonitrile solvent molecules were squeezed out (see cif-file for details. Option "SQUEEZE" of the program package PLATON).[6, 7]

1: colourless crystals, $C_{30}H_{32}BCl_4P$, M_r = 576.13, crystal size 0.22 × 0.16 × 0.04 mm, triclinic, space group *P-1* (No. 2), a = 9.2988(3) Å, b = 9.3639(3) Å, c = 17.9906(6) Å, α = 76.019(1)°, β = 78.176(1)°, γ = 69.073(1)°, V = 1407.61(8) Å^3, Z = 2, ρ = 1.359 Mg/m^{-3}, μ(Cu-K$_\alpha$) = 4.49 mm^{-1}, F(000) = 600, T = 123 K, $2\theta_{max}$ = 144.4°, 24243 reflections, of which 5485 were independent (R_{int} = 0.029), 326 parameters, R_1 = 0.047 (for 5258 I > 2σ(I)), wR_2 = 0.127 (all data), S = 1.21, largest diff. peak / hole = 0.53 / -0.37 e Å^{-3}.

1-H₂O: colourless crystals, $C_{30}H_{34}BCl_4OP$, M_r = 594.15, crystal size 0.10 × 0.04 × 0.02 mm, triclinic, space group *P-1* (No. 2), *a* = 9.6912(6) Å, *b* = 10.5427(7) Å, *c* = 15.0819(10) Å, *α* = 104.372(4)°, *β* = 105.604(4)°, *γ* = 91.201(4)°, *V* = 1431.33(17) $Å^3$, *Z* = 2, *ρ* = 1.379 Mg/m^{-3}, *μ*(Cu-$K_α$) = 4.46 mm^{-1}, *F*(000) = 620, *T* = 123 K, $2\theta_{max}$ = 145.4°, 16044 reflections, of which 5544 were independent (R_{int} = 0.061), 338 parameters, 1 restraint, R_1 = 0.093 (for 4011 I > 2σ(I)), wR_2 = 0.251 (all data), *S* = 1.05, largest diff. peak / hole = 1.28 (close to Cl-atoms) / -0.50 e $Å^{-3}$.

1-O: colourless crystals, $C_{30}H_{32}BCl_4OP \cdot C_2H_3N$, M_r = 633.19, crystal size 0.14 × 0.08 × 0.03 mm, orthoorhombic, space group *Pbca* (No. 61), *a* = 18.4148(9) Å, *b* = 16.2659(8) Å, *c* = 20.7771(10) Å, *V* = 6223.4(5) $Å^3$, *Z* = 48, *ρ* = 1.352 Mg/m^{-3}, *μ*(Cu-$K_α$) = 4.15 mm^{-1}, *F*(000) = 2640, *T* = 123 K, $2\theta_{max}$ = 144.4°, 78725 reflections, of which 6130 were independent (R_{int} = 0.035), 334 parameters, R_1 = 0.029 (for 5786 I > 2σ(I)), wR_2 = 0.075 (all data), *S* = 1.04, largest diff. peak / hole = 0.38 / -0.25 e $Å^{-3}$, in 8 voids disordered acetonitrile solvent molecules (one per void) are squeezed out.

CCDC 2109878 (**1**), 2109879 (**1-H₂O**), and 2109880 (**1-O**) contain the supplementary crystallographic data for this paper. These data can be obtained free of charge from The Cambridge Crystallographic Data Centre via www.ccdc.cam.ac.uk/data_request/cif.

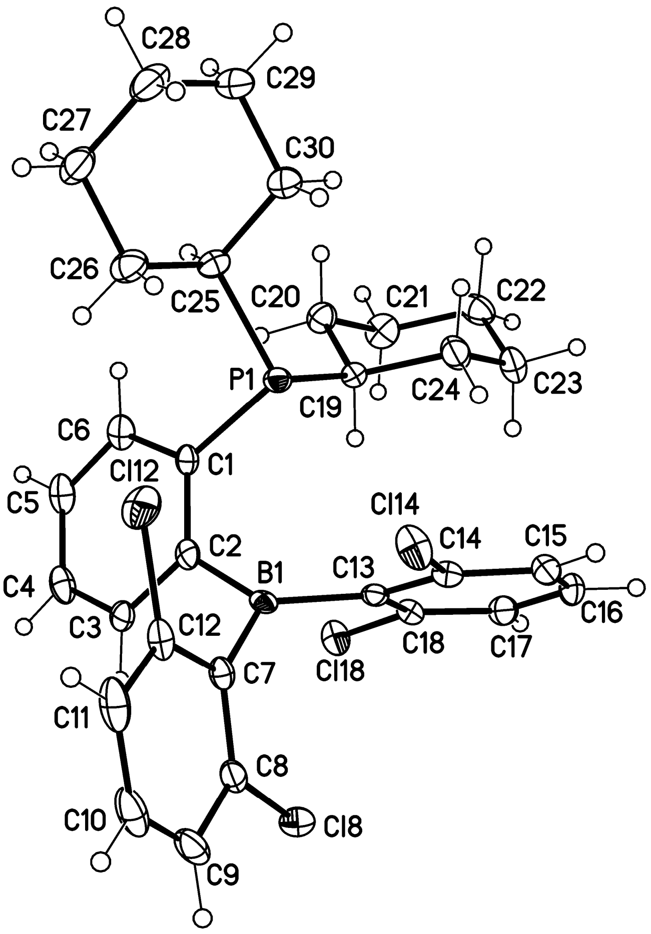

Figure S42. Molecular structure of **1** (displacement parameters are drawn at 50 % probability level).

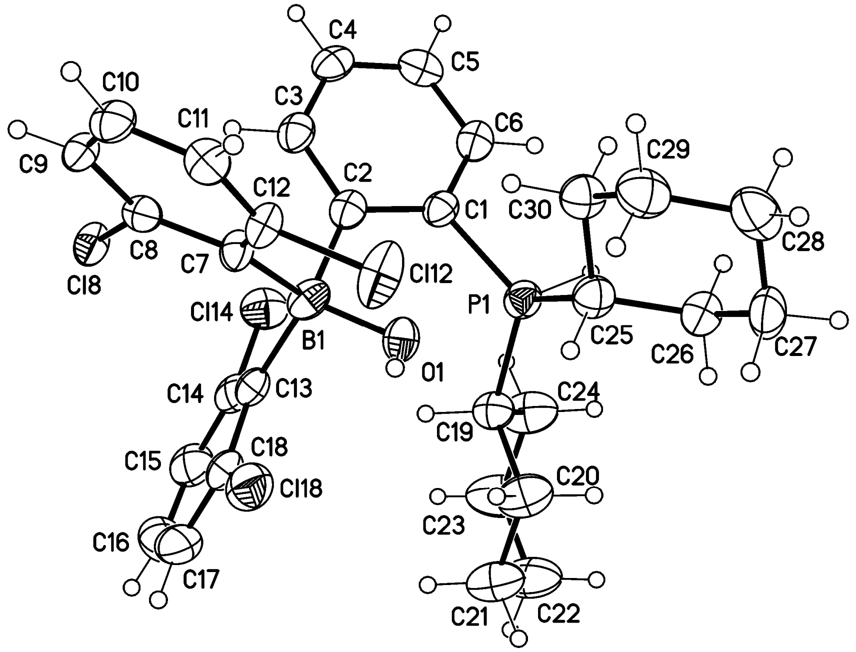

Figure S43. Molecular structure of 1-H_2O (displacement parameters are drawn at 50 % probability level).

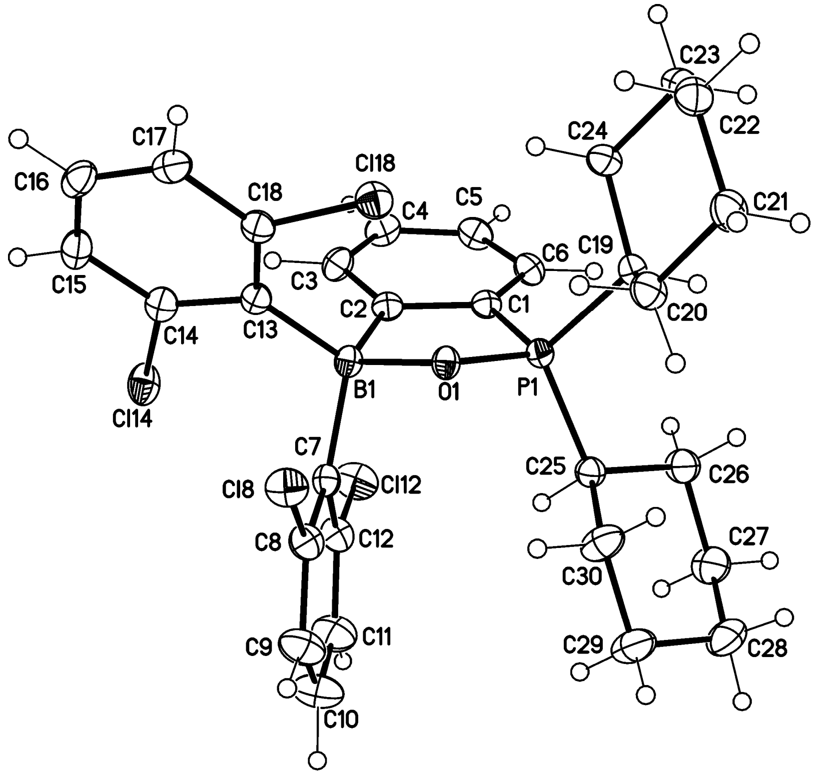

Figure S44. Molecular structure of 1-O (displacement parameters are drawn at 50 % probability level).

Kinetic studies

Sample preparation and procedure

For kinetic studies, standard solutions containing various precise concentrations of H_2O were prepared from dry and deoxygenated mixture 1:1 CH_2Cl_2:CH_3CN. The H_2O content in each standard solution was verified by Karl Fischer titrations. The utilization of non-deuterated solvents was aimed to prevent any isotopic exchange side effects. In a glovebox, **1** (10.5 mg, 18 μmol) and 0.6 ml of standard solution with the known concentration of H_2O were placed into a J. Young valve NMR tube equipped with d_6-DMSO capillary, and the sample was taken to NMR spectrometer immediately. The measurements were carried out at 25°C or 65°C (in the latter case spectrometer was preheated). The kinetic curves were built based on arrayed ^{31}P NMR spectra, which were recorded using inverse gated proton decoupling pulse sequence and long relaxation delay d1 = 60 s in order to ensure quantitative measurements. The concentration of **1-H₂O** at each time point was determined as follows: the intensity of the ^{31}P NMR signal corresponding to **1-H₂O** was normalized to the total ^{31}P NMR signals intensity and then was referred to its initial concentration (**[1-H₂O]₀** = **[1]₀** = 30mmol/L). See the details below.

25 °C experiments

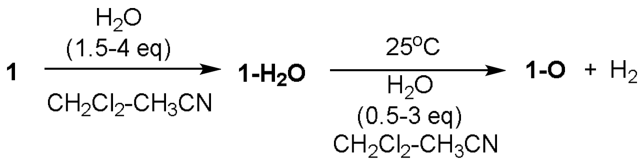

Water that was not a part of **1-H₂O** was assumed not to be consumed in the experiments, hence the kinetic aquation $v = k[\mathbf{1\text{-}H_2O}]^a[H_2O]^b$ can be simplified to $v = k_{eff}[\mathbf{1\text{-}H_2O}]^a$ with the best fit found for a = 1 (Figure S45). The respective k_{eff} values are reported in Table S1. Ultimately, from the dependence of $k_{eff} = k[H_2O]^b$ from initial concentrations of water, was found b = -0.5.

Table S1. Kinetic experiments performed at 25 °C: initial concentrations and extracted k_{eff} values for the first order in [**1-H₂O**] kinetic fits.

Experiment	$[\mathbf{1\text{-}H_2O}]_0$ mM	$[H_2O]$ mM	$[H_2O]/[\mathbf{1\text{-}H_2O}]$	k_{eff}, h^{-1}
1	30	90	3	0.079
2	30	60	2	0.0994
3	30	45	1.5	0.1246
4	30	30	1	0.1476
5	30	15	0.5	0.1887

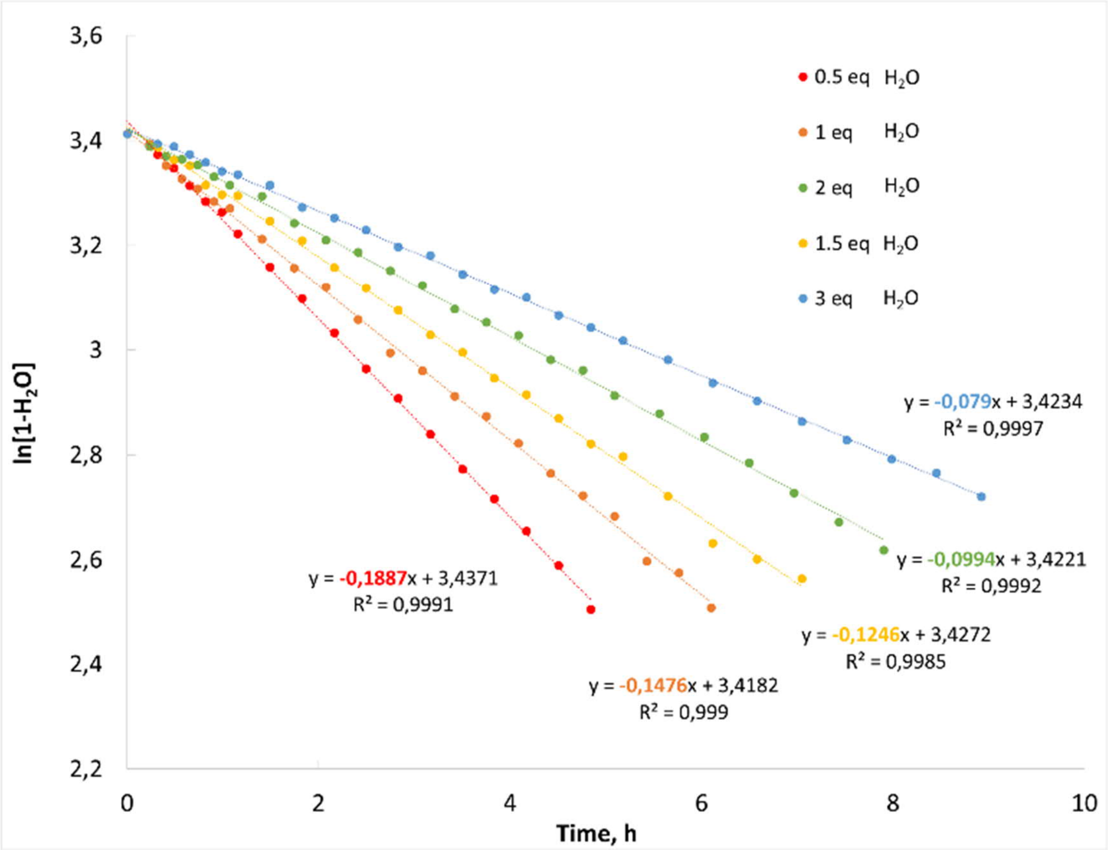

Figure S45. Linear correlation between ln[**1-H$_2$O**] and time, showing first order in **1-H$_2$O** at 25°C.

65 °C experiments

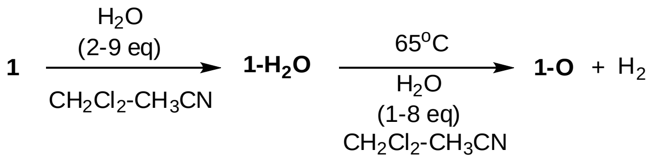

Similar as for 25 °C experiments treatment of data obtained at 65 °C allowed to found second order in **1-H$_2$O** (Figure S46) and the respective k_{eff} (Table S2). Further treatment of k_{eff}/[H$_2$O]$_0$ dependences pointed to order -1 in water. More thorough analysis was done below in the Kinetic modeling section.

Table S2. Kinetic experiments performed at 65 °C: initial concentrations and extracted k_{eff} values for the first order in [**1-H$_2$O**] kinetic fits.

**Experiment**	**[1-H$_2$O]$_0$ mM**	**[H$_2$O] mM**	**[H$_2$O]/ [1-H$_2$O]**	**k_{eff}, mM^{-1}h^{-1}**
1	30	240	8	0,0012
2	30	150	5	0,0045
3	30	60	2	0,0098
4	30	30	1	0,0119

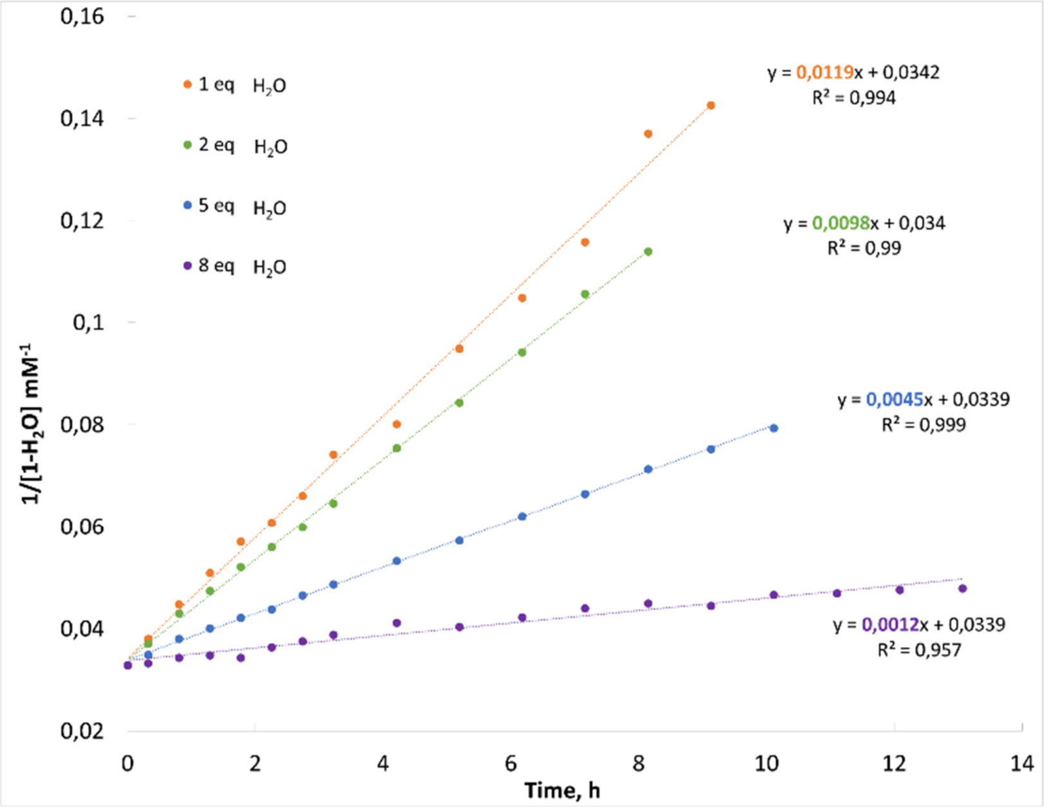

Figure S46. Linear correlation between 1/[**1-H$_2$O**] and time, showing second order in **1-H$_2$O** at 65°C.

Kinetic modeling

Kinetic modelling was carried out using COPASI program.[8] Kinetic data collected at 65 °C were fit to the following model:

$$\mathbf{1-H_2O} \overset{k_1\ k_{-1}}{\rightarrow\longleftarrow} \mathbf{1} + \mathrm{H_2O} \quad (1)$$

$$\mathbf{1-H_2O} + \mathbf{1} \overset{k_2}{\rightarrow} \mathbf{1-O} + \mathrm{H_2} + \mathbf{1} \quad (2)$$

that reflects catalytic properties of **1** in conversion of **1-H$_2$O** adduct to **1-O** and H_2.

Fitting observed concentrations to the model was possible with RMSE 2.4 mM. Despite the challenges of obtaining higher quality data and simplicity of the model, the obtained model parameters agreed well with observations and calculations (Fig. S47). Particularly, parameter correlation matrix (see below the report) revealed that the values of k_1 and k_{-1} are fully positively correlated and their optimal values are significantly higher than the rate constant k_2. This supports the hypothesis that dissociation of adduct **1-H$_2$O** is a rapid equilibrium process in comparison to the rate determining second step. Extracted equilibrium constant K = k_1/k_{-1} = 2.86 mM corresponds to □G = 3.9 kcal/mol , a very close value to the predicted dissociation constant of **1-H$_2$O** (3.3 kcal/mol at 25 °C). Extracted k_2 value (0.183 l/(mmol*h) or 0.051 l/(mol*s)) corresponds to the activation free energy of 21.9 kcal/mol.

Importantly, ^{31}P NMR kinetic measurements for the experiment with the 1:1 initial **1**:H_2O ratio allowed to trace dissociation of **1-H$_2$O**: the signal of free **1** reached 1.73 mM concentration and

gradually decreased to trace levels. At the same time, 6.7 mM peak concentration of **1** was predicted by the obtained kinetic model. Besides this inconsistency, the conversion of **1-H$_2$O** into **1-O** did not reach completion and the kinetic curve flattened at ~5 mM of **1-H$_2$O**. We attributed such behaviour to low equilibrium concentrations of water present in the reaction mixture. Water might be crucial for endo-exo isomerization step, a process that was out of scope of our mechanistic studies. Presumably, the same isomerization stage could become a rate limiting step at room temperature and alter the kinetic orders.

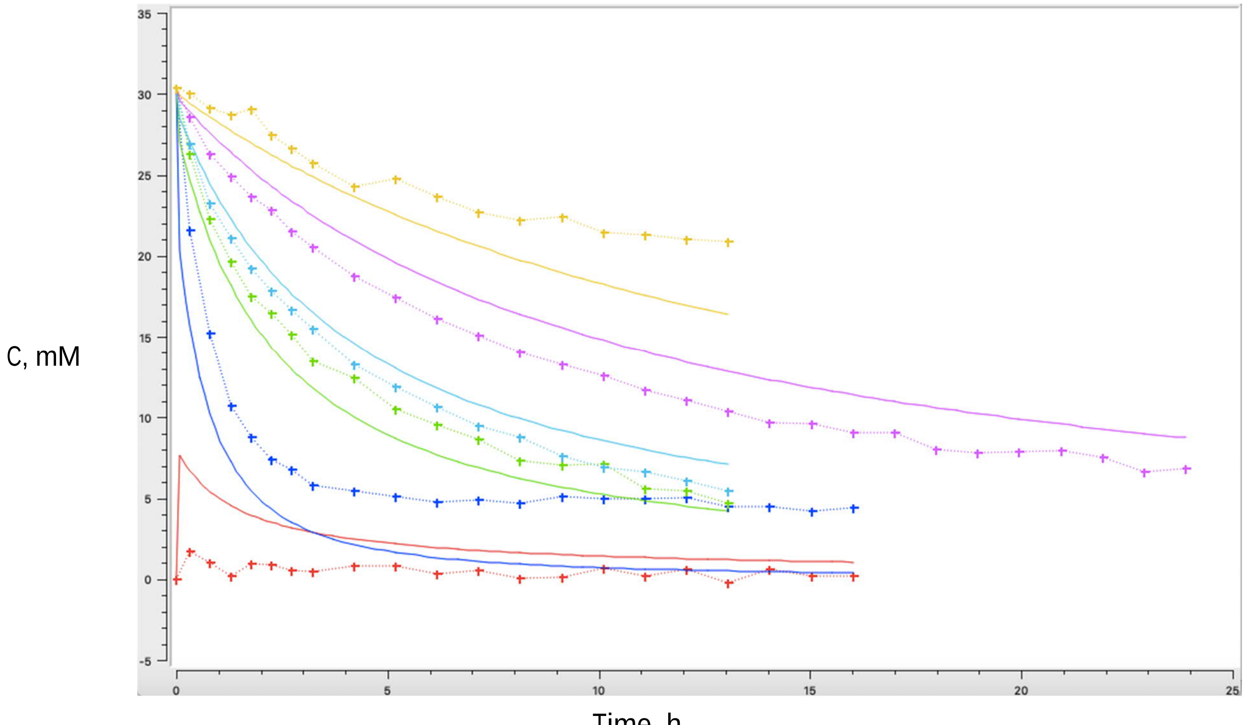

Figure S47. Simulated (solid) and experimental (dashed) kinetic curves for PB-H$_2$O concentrations with 8 (yellow), 5 (magenta), 2 (cyan), 1 (green) and 0 (blue) equivalents of water. Red traces correspond to PB curve in the experiment with 0 equivalents of water.

Kinetic modeling report:

```
Objective Function Value:    719.958
Standard Deviation:   2.42926
Function Evaluations: 3779
CPU Time [s]:  5.593
Evaluations/Second [1/s]:    675.666

       Parameter       Value   Gradient       Standard Deviation
       (Diss).k1:      181091  5.33333e-05    1.23477e+07
       (Diss).k2:      63210.7 -6.07014e-05   4.31226e+06
       (Water reduction).k1: 0.183103         69.12630.0265886

File Name:     /Users/K/Documents/water reduction/0_eq_mod.txt
Experiment:    Experiment_4
Mean:   -0.913525
Objective Value:      423.678
Root Mean Square:     3.17609
```

Row	Time	[PB](Data)	[PB](Fit)	[PB](Weighted Error)	[PB-H2O](Data)	[PB-H2O](Fit)	[PB-H2O](Weighted Error)	Objective Value	Root Mean Square
1.	0	0	0	0	30.373	30.373	0	0	0
2.	0.3175	1.73253	6.70163	4.9691	21.5929	15.6766	-5.91623	59.6937	5.46323
3.	0.801667	1.04029	5.40591	4.36562	15.2209	10.2007	-5.02015	44.2606	4.70429
4.	1.28583	0.222037	4.55883	4.33679	10.7209	7.25434	-3.4666	30.825	3.92588
5.	1.77	0.92972	3.96063	3.03091	8.78515	5.47547	-3.30968	20.1404	3.17336
6.	2.25417	0.878534	3.51496	2.63642	7.41503	4.31253	-3.1025	16.5762	2.87891
7.	2.73833	0.52283	3.16956	2.64673	6.74799	3.50662	-3.24137	17.5116	2.95902
8.	3.2225	0.494255	2.89365	2.39939	5.76193	2.9227	-2.83923	13.8183	2.62853

9.	4.20667	0.84525	2.47399	1.62874	5.42194	2.13642	-3.28552	13.4475	2.59302
10.	5.19083	0.815816	2.17456	1.35874	5.07873	1.65057	-3.42816	13.5985	2.60753
11.	6.175	0.295913	1.94945	1.65353	4.72534	1.32652	-3.39882	14.2862	2.67265
12.	7.15917	0.536335	1.77357	1.23723	4.90625	1.09796	-3.80829	16.0338	2.83142
13.	8.14333	0.0855635	1.63203	1.54647	4.71902	0.929715	-3.78931	16.7504	2.894
14.	9.1275	0.120142	1.51545	1.39531	5.13183	0.801631	-4.3302	20.6975	3.21695
15.	10.1117	0.678386	1.41758	0.739197	4.97714	0.701438	-4.27571	18.8281	3.06823
16.	11.0958	0.156191	1.33414	1.17795	4.98022	0.621288	-4.35893	20.3878	3.19279
17.	12.08	0.5991	1.26204	0.662943	5.03515	0.555957	-4.47919	20.5026	3.20177
18.	13.0642	-0.197733	1.19906	1.39679	4.50327	0.501851	-4.00142	17.9624	2.99687
19.	14.0483	0.602197	1.1435	0.541306	4.45535	0.456422	-3.99893	16.2844	2.85346
20.	15.0325	0.192779	1.09408	0.901304	4.18656	0.417823	-3.76874	15.0157	2.74005
21.	16.0167	0.184879	1.0498	0.864919	4.42316	0.384683	-4.03848	17.0574	2.92039
Objective Value				110.738			312.94		
Root Mean Square				2.29635			3.8603		
Weight				1			1		

File Name: /Users/K/Documents/water reduction/1_eq.txt
Experiment: Experiment_2
Mean: -1.45161
Objective Value: 44.3874
Root Mean Square: 1.57034

Row	Time	[PB-H2O](Data)	[PB-H2O](Fit)	[PB-H2O](Weighted Error)	Objective Value	Root Mean Square
1.	0	30.373	30.373	0	0	0
2.	0.3175	26.2548	24.6852	-1.56963	2.46375	1.56963
3.	0.801667	22.292	20.9423	-1.34963	1.82151	1.34963
4.	1.28583	19.6021	18.1679	-1.43427	2.05714	1.43427
5.	1.77	17.5055	16.0322	-1.47338	2.17084	1.47338
6.	2.25417	16.4386	14.3392	-2.09944	4.40765	2.09944
7.	2.73833	15.1356	12.9652	-2.17038	4.71056	2.17038
8.	3.2225	13.4713	11.8285	-1.6428	2.69879	1.6428
9.	4.20667	12.4679	10.0339	-2.43403	5.92452	2.43403
10.	5.19083	10.5359	8.70791	-1.82797	3.34149	1.82797
11.	6.175	9.53896	7.68895	-1.85001	3.42253	1.85001
12.	7.15917	8.63346	6.88188	-1.75157	3.068	1.75157
13.	8.14333	7.29432	6.22709	-1.06723	1.13897	1.06723
14.	9.1275	7.00989	5.68536	-1.32453	1.75439	1.32453
15.	10.1117	7.13281	5.22982	-1.90299	3.62138	1.90299
16.	11.0958	5.62222	4.8415	-0.780713	0.609513	0.780713
17.	12.08	5.48136	4.50658	-0.974784	0.950204	0.974784
18.	13.0642	4.69034	4.21479	-0.475555	0.226153	0.475555
Objective Value				44.3874		
Root Mean Square				1.57034		
Weight				1		

File Name: /Users/K/Documents/water reduction/2_eq.txt
Experiment: Experiment_1
Mean: 1.14826
Objective Value: 27.0206
Root Mean Square: 1.22521

Row	Time	[PB-H2O](Data)	[PB-H2O](Fit)	[PB-H2O](Weighted Error)	Objective Value	Root Mean Square
1.	0	30.373	30.373	0	0	0
2.	0.3175	26.9129	27.0182	0.10537	0.0111029	0.10537
3.	0.801667	23.2362	24.4289	1.19267	1.42247	1.19267
4.	1.28583	21.0782	22.2893	1.21116	1.46691	1.21116
5.	1.77	19.1876	20.4922	1.30457	1.70191	1.30457
6.	2.25417	17.8327	18.9617	1.12899	1.27462	1.12899
7.	2.73833	16.6903	17.6427	0.952344	0.906958	0.952344
8.	3.2225	15.4596	16.4944	1.03472	1.07065	1.03472
9.	4.20667	13.256	14.5652	1.30923	1.71408	1.30923
10.	5.19083	11.8567	13.0385	1.18178	1.39661	1.18178
11.	6.175	10.6153	11.8004	1.18514	1.40456	1.18514
12.	7.15917	9.46653	10.7764	1.30984	1.71567	1.30984
13.	8.14333	8.77444	9.91533	1.14089	1.30162	1.14089
14.	9.1275	7.60616	9.18134	1.57518	2.48119	1.57518
15.	10.1117	6.93682	8.54824	1.61143	2.59669	1.61143
16.	11.0958	6.61341	7.99661	1.3832	1.91325	1.3832
17.	12.08	6.07764	7.5117	1.43405	2.05651	1.43405
18.	13.0642	5.47404	7.0821	1.60806	2.58584	1.60806
Objective Value				27.0206		
Root Mean Square				1.22521		
Weight				1		

File Name: /Users/K/Documents/water reduction/5_eq.txt
Experiment: Experiment_3

Mean: 1.96465
Objective Value: 122.41
Root Mean Square: 2.05452

Row	Time	[PB-H2O](Data)	[PB-H2O](Fit)	[PB-H2O](Weighted Error)	Objective Value	Root Mean Square
1.	0	30.373	30.373	0	0	0
2.	0.3175	28.5778	28.8887	0.310858	0.096633	0.310858
3.	0.801667	26.2534	27.5849	1.33146	1.77279	1.33146
4.	1.28583	24.8921	26.3935	1.50148	2.25445	1.50148
5.	1.77	23.6836	25.3007	1.61712	2.61509	1.61712
6.	2.25417	22.8072	24.2946	1.4874	2.21235	1.4874
7.	2.73833	21.483	23.3654	1.88238	3.54334	1.88238
8.	3.2225	20.5279	22.5046	1.97666	3.90718	1.97666
9.	4.20667	18.7456	20.9364	2.19082	4.79968	2.19082
10.	5.19083	17.442	19.5724	2.13034	4.53835	2.13034
11.	6.175	16.1113	18.375	2.26372	5.12445	2.26372
12.	7.15917	15.0361	17.3155	2.2794	5.19566	2.2794
13.	8.14333	14.0145	16.3715	2.35702	5.55553	2.35702
14.	9.1275	13.2813	15.525	2.24367	5.03407	2.24367
15.	10.1117	12.5933	14.7616	2.16833	4.70167	2.16833
16.	11.0958	11.6589	14.0698	2.41089	5.8124	2.41089
17.	12.08	11.061	13.4398	2.37884	5.65888	2.37884
18.	13.0642	10.3418	12.8638	2.52201	6.36051	2.52201
19.	14.0483	9.67477	12.3352	2.66039	7.07766	2.66039
20.	15.0325	9.59378	11.8482	2.2544	5.0823	2.2544
21.	16.0167	9.03491	11.3982	2.36326	5.58499	2.36326
22.	17.0008	9.04766	10.9811	1.93342	3.7381	1.93342
23.	17.985	8.01527	10.5934	2.57814	6.6468	2.57814
24.	18.9692	7.81457	10.2322	2.41759	5.84473	2.41759
25.	19.9533	7.85843	9.89473	2.0363	4.14652	2.0363
26.	20.9375	7.97032	9.57883	1.60851	2.58731	1.60851
27.	21.9217	7.52963	9.28246	1.75284	3.07244	1.75284
28.	22.9058	6.59527	9.00388	2.40861	5.80141	2.40861
29.	23.89	6.83241	8.74152	1.9091	3.64467	1.9091
Objective Value					122.41	
Root Mean Square					2.05452	
Weight				1		

File Name: /Users/K/Documents/water reduction/8_eq.txt
Experiment: Experiment
Mean: -1.98678
Objective Value: 102.462
Root Mean Square: 2.38586

Row	Time	[PB-H2O](Data)	[PB-H2O](Fit)	[PB-H2O](Weighted Error)	Objective Value	Root Mean Square
1.	0	30.373	30.373	0	0	0
2.	0.3175	30.0047	29.422	-0.582799	0.339655	0.582799
3.	0.801667	29.0981	28.5551	-0.542959	0.294804	0.542959
4.	1.28583	28.6928	27.7379	-0.954905	0.911843	0.954905
5.	1.77	29.0488	26.9661	-2.0827	4.33763	2.0827
6.	2.25417	27.4298	26.2361	-1.19375	1.42505	1.19375
7.	2.73833	26.6209	25.5445	-1.0764	1.15864	1.0764
8.	3.2225	25.7288	24.8884	-0.840436	0.706333	0.840436
9.	4.20667	24.2561	23.6535	-0.602596	0.363122	0.602596
10.	5.19083	24.7531	22.5353	-2.2178	4.91862	2.2178
11.	6.175	23.6454	21.518	-2.1275	4.52624	2.1275
12.	7.15917	22.6978	20.5885	-2.10929	4.44912	2.10929
13.	8.14333	22.2137	19.7359	-2.47777	6.13936	2.47777
14.	9.1275	22.4366	18.9512	-3.4854	12.148	3.4854
15.	10.1117	21.4083	18.2264	-3.18192	10.1246	3.18192
16.	11.0958	21.275	17.555	-3.72	13.8384	3.72
17.	12.08	20.9969	16.9313	-4.06555	16.5287	4.06555
18.	13.0642	20.8506	16.3504	-4.50019	20.2517	4.50019
Objective Value					102.462	
Root Mean Square					2.38586	
Weight				1		

Fisher Information Matrix:
Matrix(3x3)

1.72516e-07	-4.9423e-07	0.171468
-4.9423e-07	1.41589e-06	-0.491242
0.171468	-0.491242	179272

FIM Eigenvalues:
Matrix(3x1)

3.44981e-14
7.83011e-08
179272

FIM Eigenvectors corresponding to Eigenvalues:
Matrix(3x3)

-0.944083	-0.329707	-4.78592e-10
0.329707	-0.944083	-2.90233e-06
9.56468e-07	-2.7402e-06	1

Fisher Information Matrix (scaled):
Matrix(3x3)

5657.5	-5657.41	5685.61
-5657.41	5657.32	-5685.67
5685.61	-5685.67	6010.42

```
FIM Eigenvalues (scaled):
  Matrix(3x1)
       0.000634329
       195.788
       17129.4

FIM Eigenvectors (scaled) corresponding to Eigenvalues:
  Matrix(3x3)
       0.706923      0.707291      0.000355621
       -0.414665     0.414041      0.810323
       0.572987      -0.572983     0.585984

Correlation Matrix:
  Matrix(3x3)
       1      0.999998      0.235202
       0.999998      1      0.237222
       0.235202      0.237222      1
```

DFT calculations

Methods

All calculations were carried out using Gaussian 09 program[9] and visualized with CYLview 2.0.[10] The geometries were optimized at ωB97XD[11]/6-311G**[12] level of theory in vacuo followed by the vibrational analysis. Equilibrium geometries and transition states were verified by vibrational analysis. Transition states were additionally verified by either prior PES scans that provided initial geometries for the TS search or by IRC calculations. Thermal correction accounted for 298.15 K temperature and 24.465 atm pressure (corresponding to the 1M solution concentrations) at the same level of theory. SMD implicit solvation model was used to account for solvent correction.[13] Ultimately, electronic energies were refined at the ωB97XD/6-311++G(3df,3pd) level of theory and together with the solvation and thermal corrections were reported as solution phase Gibbs free energies. For **1**, **4**, and their water and H_2 adducts conformational search was performed with OPLS force filed in Macromodel.[14] Produced conformers were optimized with DFT, and the lowest in final energy structure was reported. In general, good consistency were observed with the geometries obtained from XRD results.

Hydrogen and water addition to 1

Addition of water was found to be destabilized in acetonitrile in comparison to DCM and water, whereas stability of the H_2 adducts were growing with the increase of solvent polarity (Figure S48). For the sake of computational cost, computational studies of the water reduction mechanism were carried out with the model deschlorophsophinoborane **4**.

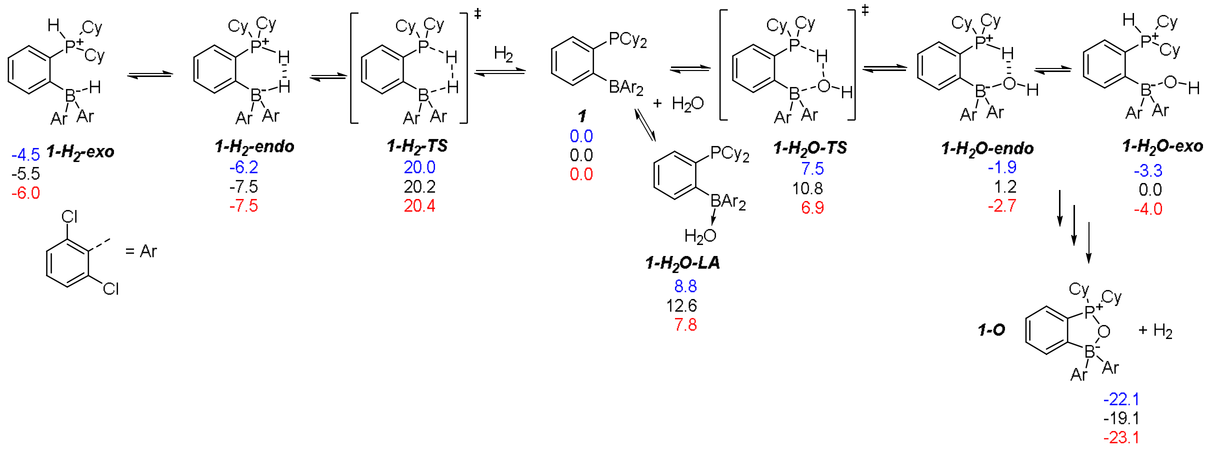

Figure S48. Gibbs free energies (in kcal/mol) for derivatives of **1**, H_2, and H_2O in dichloromethane (blue), acetonitrile (black) and water (red) solutions.

Optimized geometries of derivatives of **1**:

1	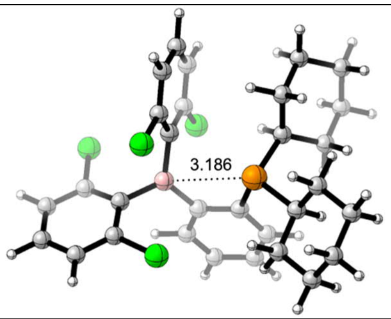
Endo-1-H_2	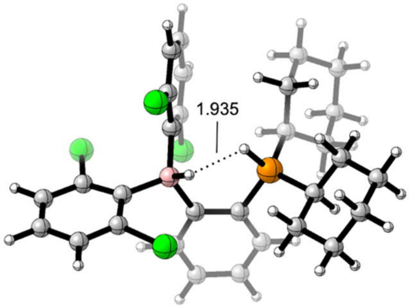
Endo-1-H_2' (conformer)	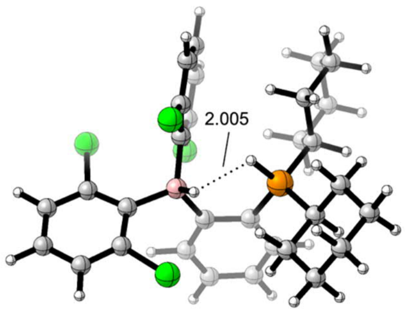
Exo-1-H_2	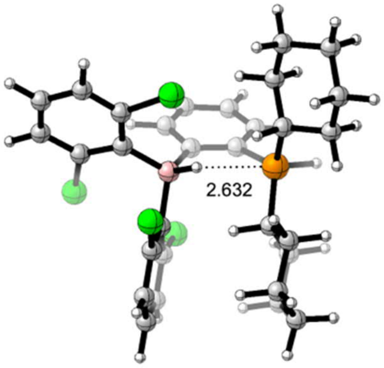
1-H_2-TS	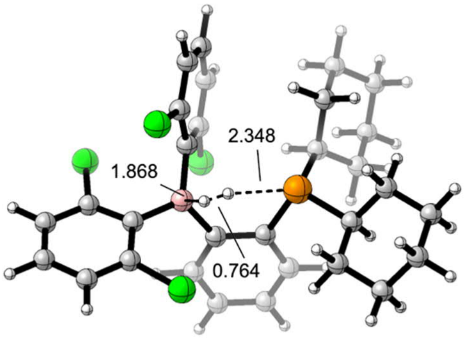

1-H_2-TS' (conformer)	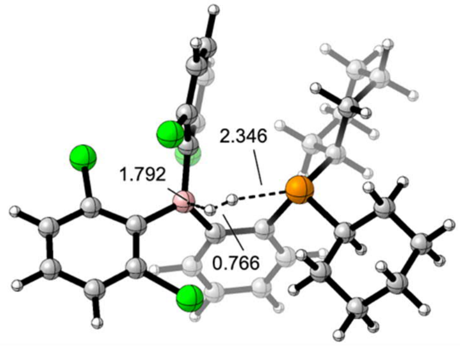
1-H_2O-LA	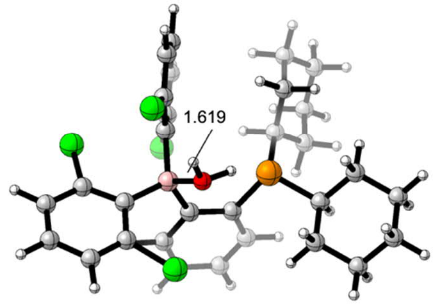
1-H_2O-endo	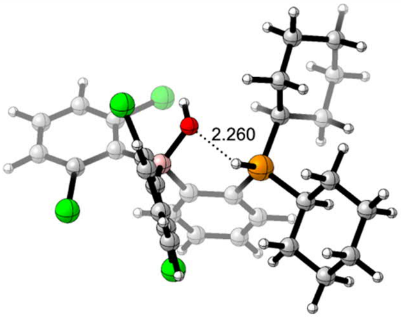
1-H_2O-exo	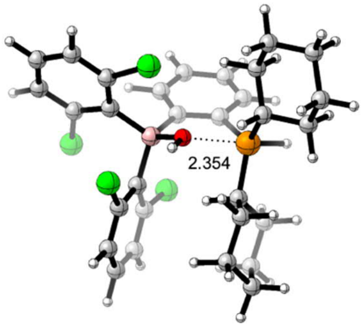
1-H_2O-TS	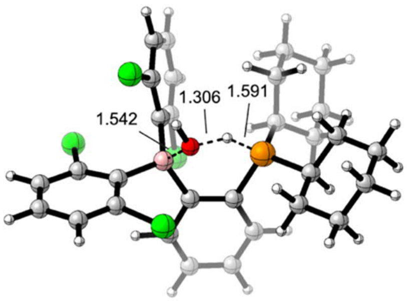

1-O	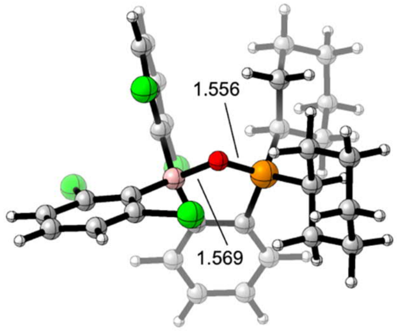

Computationally modelled mechanism of water reduction by 4 as model phosphinoborane and triphenylborane (5) as a catalyst

Initially, the bimolecular (with respect to phosphinoborane) mechanism of water reduction was identified for triphenylborane (**5**) as the catalyst (Figure S49). Hydride abstraction by **5** can be performed starting from either **4-H$_2$O-exo** or **4-H$_2$O-endo** water adducts through transition states **exo-4-H$_2$O-5-TS** or **endo-4-H$_2$O-5-TS**, respectively. The latter transition state is less energetically favored by 15.4 kcal/mol, presumably, due to the stronger steric repulsion in this geometry. Two generated ions, triphenylborohydride **[5-H]$^-$** and protonated phosphine oxide **[4-OH]$^+$**, then react via **[5-H][4-OH]-TS** to produce H_2, intramolecular Lewis adduct **4-O**, and recycle **5**. The key transition states were lowered in more polar media, such as acetonitrile and water. **[5-H][4-OH]-TS** seems to lie lower than the respective starting materials, however, the separated ions **[5-H]$^-$** and **[4-OH]$^+$** might not be adequate description of the species, as shown below for **[4-H]$^-$** and **[4-OH]$^+$** for which ionic pair was found to be less energetic.

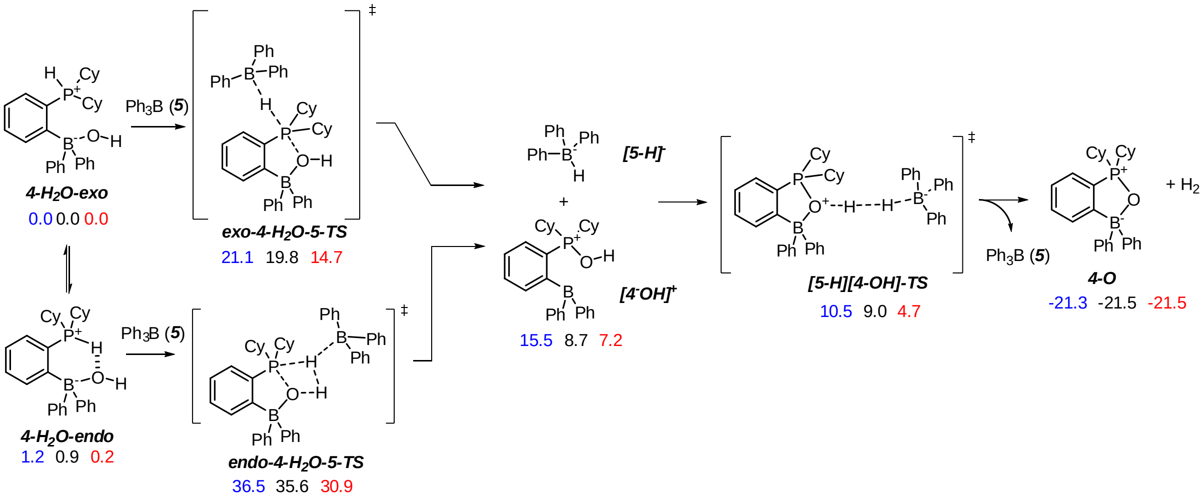

Figure S49. Bimolecular mechanism of water reduction by model phospinoborane **4** catalyzed by triphenylborane **5**. Gibbs free energies (in kcal/mol) for solutions in dichloromethane (blue), acetonitrile (black) and water (red) are reported relatively to **4-H$_2$O-exo** and **5**.

Computational modeling of water reduction mechanism with 4 as model phosphinoborane

Bimolecular water reduction by phosphinoboranes **4** alone (Figure S50) is similar to the mechanism modelled for **4** catalyzed by **5**. Only one key transition state **4-H$_2$O-4-TS** that is similar to **exo-4-H$_2$O-5-TS**, the lowest of two previously found hydrogen umpolung TSs, was considered.

Energies are reported relatively to two molecules of **4-H$_2$O-exo** as the ground state. Accordingly, in the steps where **4** is involved as the catalyst, the energy of the **4-H$_2$O-exo** dissociation into **4** and water is taken into account. This dissociation energy, however, cannot completely account for the energy differences between **4-H$_2$O-4-TS** and **exo-4-H$_2$O-5-TS**, and it is assumed that the higher steric hindrance of **4** in comparison to **5** may play a role. **4-H$_2$O-4-TS** is the highest point on the energy profile, however, H_2 recombination transition state **[4-H][4-OH]-TS** lies only 6-7 kcal/mol lower**.** In some solvents, e.g. DCM, the energy of the corresponding separated ions **[4-H]**$^-$ and **[4-OH]**$^+$ is higher than the energy of the TS. For this reason, the respective ionic pair **[4-H][4-OH]-IP** was optimized and found to be lower in energy than the respective TS, in all the solvent studied.

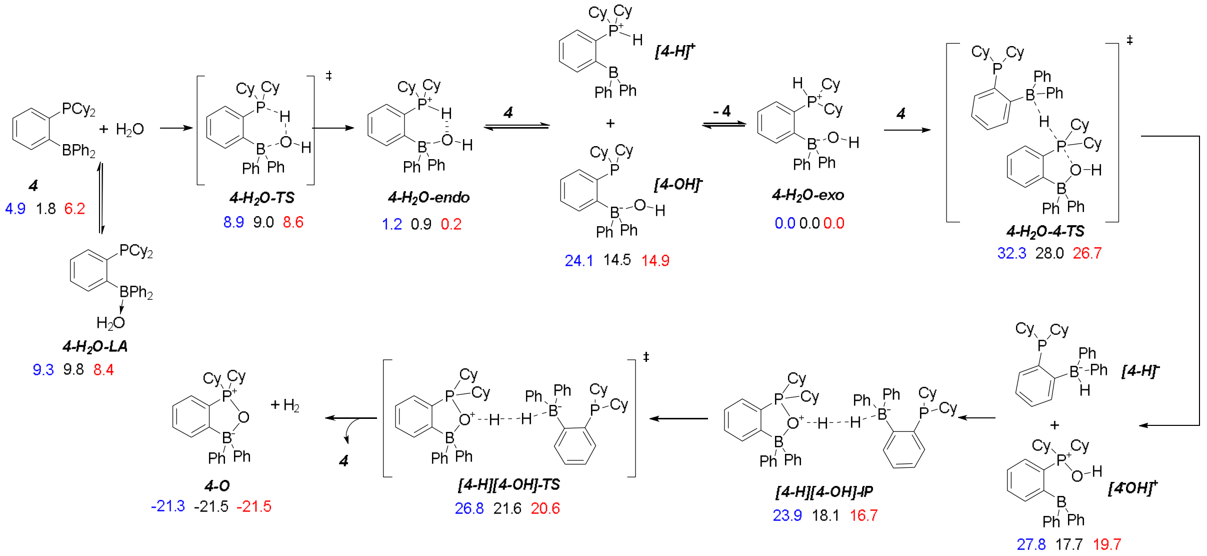

Figure S50. Bimolecular mechanism of water reduction by model phospinoborane **4** catalyzed by **4**. Gibbs free energies (in kcal/mol) for solutions in dichloromethane (blue), acetonitrile (black) and water (red) are reported relatively to **4-H$_2$O-exo**.

The part of the mechanism that was out of scope of the current studies was isomerization between exo and endo water adducts of **1** and **4**. **1-H$_2$O-exo** was isolated in our studies while computational modelling showed that **1-H$_2$O-exo** and **4-H$_2$O-exo** are more stable than the respective endo adducts. In principle, exo-adducts can form directly via intermolecular water splitting between two molecules of phosphinoborane and water, followed by proton transfer. Similarly, exo-adducts can be produced by proton transfer from the inner molecular cavity to the outer sphere of **1-H$_2$O-endo** or **4-H$_2$O-endo**. Because there are various molecules that can be potential proton transfer agents, this process was not modelled per se. Yet, two ions, **[4-H]**$^+$ and **[4-OH]**$^-$, that may originate from the proton transfer from **4-H$_2$O-endo** to **4** were optimized (Figure S50). Their total energy is significantly lower than of **4-H$_2$O-4-TS** that indicates some possibility for a rapid endo-exo isomerization process.

The corresponding endo and exo H_2 adducts of **4** were also studied computationally (Figure S51). Hydrogen addition to **4** in DCM is by 3.9 kcal/mol less favorable than addition of water, whereas the reaction itself is slightly exergonic (-1 kcal/mol). In acetonitrile, hydrogen addition becomes more preferred product.

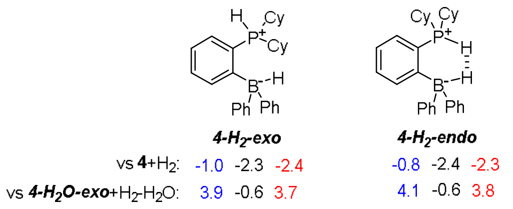

Figure S51. Gibbs free energies (in kcal/mol) for hydrogen addition to **4** in dichloromethane (blue), acetonitrile (black) and water (red) solutions reported relatively to **4-H₂O-exo** or **4**

Computational study of monomolecular water reduction mechanisms

Two alternative monomolecular (with respect to phosphinoborane) mechanisms were studied computationally (Figure S52). Direct hydrogen release from the **4-H$_2$O-endo** could procced via 4-centered transition state **4c-TS**. This transition state lies 10 kcal/mol higher in energy than the bimolecular **4-H$_2$O-4-TS**. For this reason and due to different kinetic pattern, it was concluded the water reduction by **1** undergoes via bimolecular mechanism and transition state similar to **4-H$_2$O-4-TS**.

Another studied monomolecular mechanism features formation of upside-down FLP water adduct **4-H$_2$O-upsd** followed by intramolecular recombination of protic and hydridic hydrogen atoms into the product **4-O** and H_2. Although umpolung water splitting by **4** is hindered by prohibiting energetic barrier lying above 90 kcal/mol, it is remarkable that **4-H$_2$O-upsd** appears to be only slightly endergonic (4.4 kcal/mol) in comparison to the water adduct **4-H$_2$O-exo**. Moreover, **4-H$_2$O-upsd** is hindered by a rather high 10.3 kcal/mol kinetic barrier (**4-H2O-upsd-TS2**) against hydrogen release that indicates a hypothetic possibility of detecting such compound if prepared via alternative methods at cryogenic temperatures.

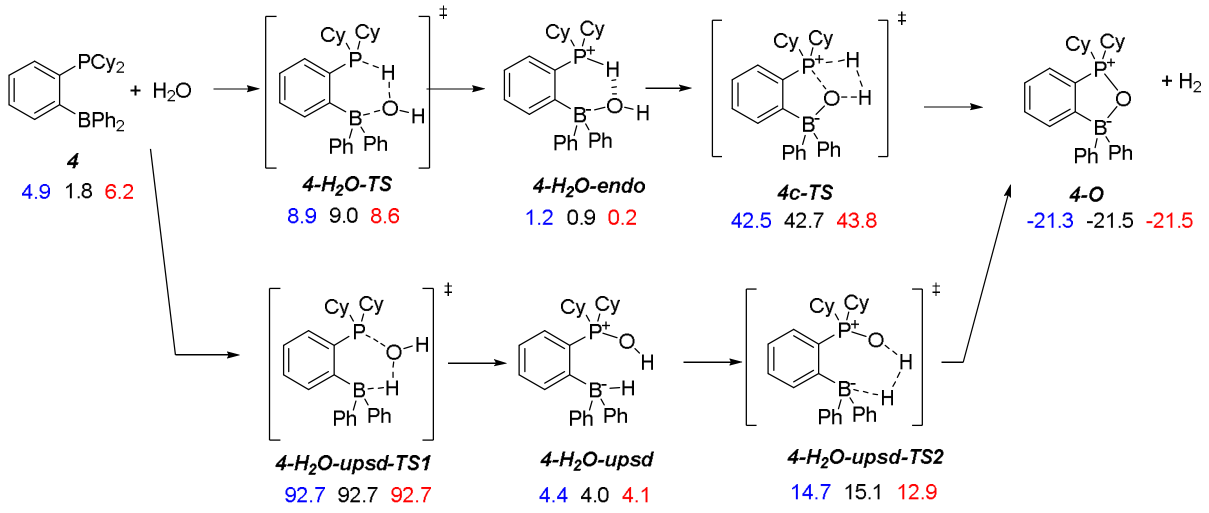

Figure S52. Monomolecular mechanisms of water reduction by model phospinoborane **4** via 4-centered transition state (top) and umpolung FLP adduct **4-H$_2$O-upsd** (bottom). Gibbs free energies (in kcal/mol) for solutions in dichloromethane (blue), acetonitrile (black) and water (red) are reported relatively to **4-H$_2$O-exo**.

Optimized geometries of derivatives of **4** and **5**

4	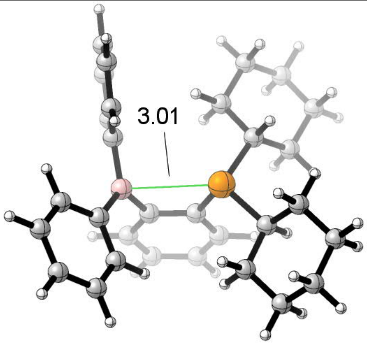
5	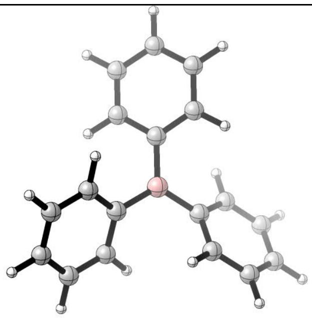
$[5\text{-H}]^-$	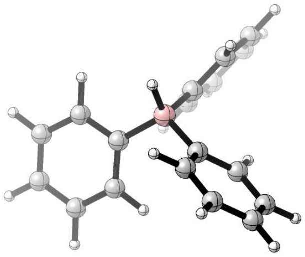
4-O	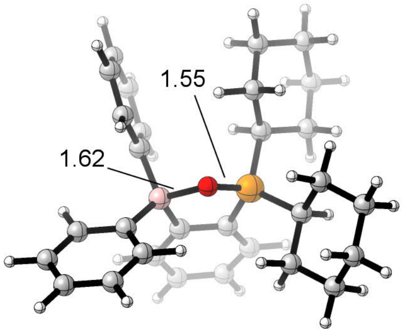

4-H_2O-endo'	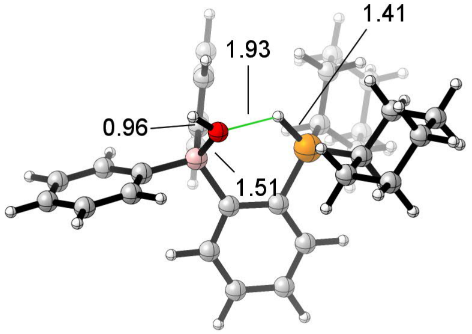
4-H_2O-endo	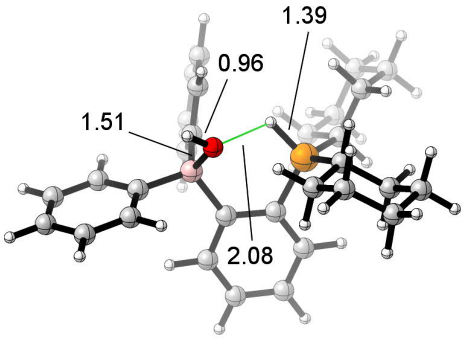
4-H_2O-exo	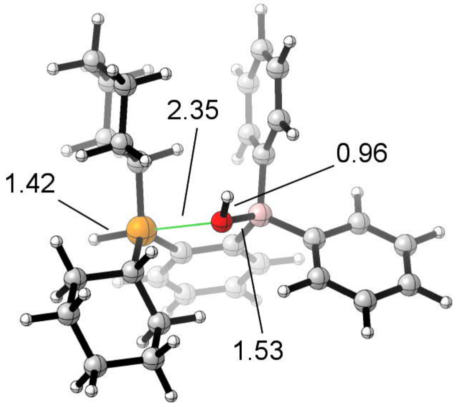
4-H_2O-exo'	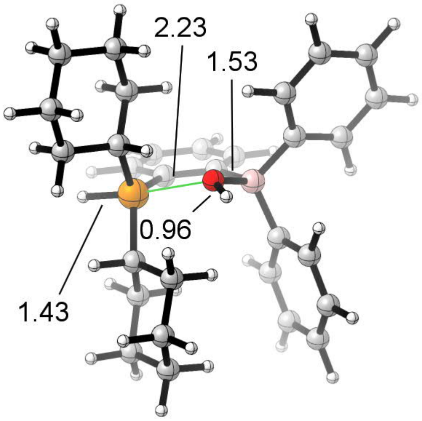

4-H_2O-LA	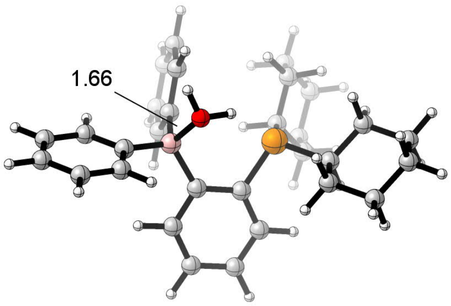
4-H_2O-TS	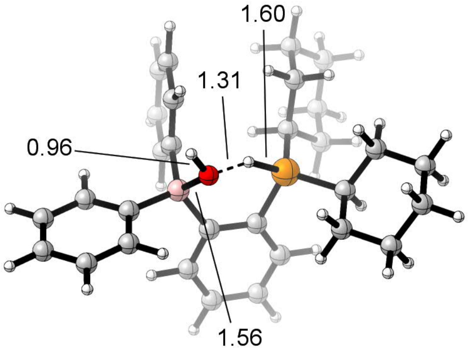
$[4\text{-H}]^+$	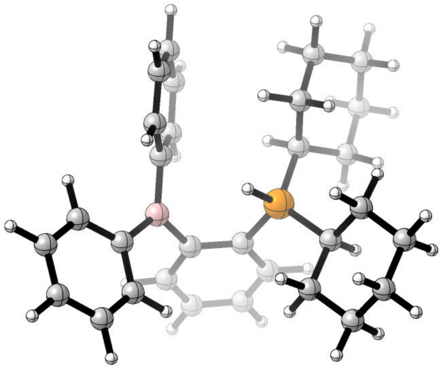
$[4\text{-OH}]^-$	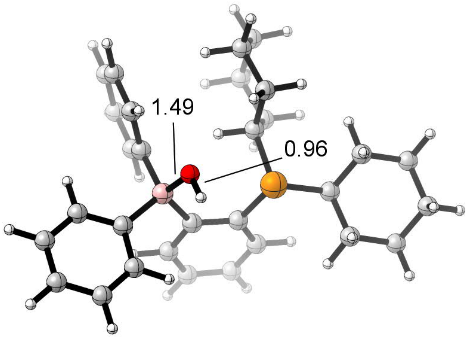

[4-OH]+	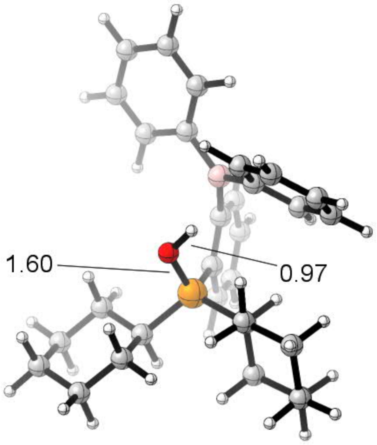
[4-H]-	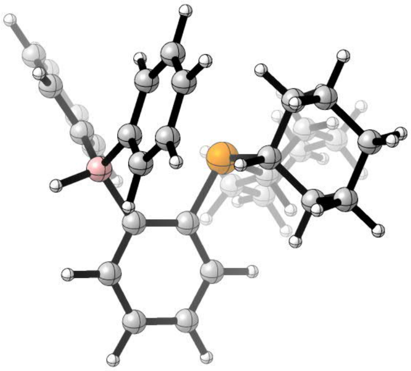
endo-4-H_2O-5-TS	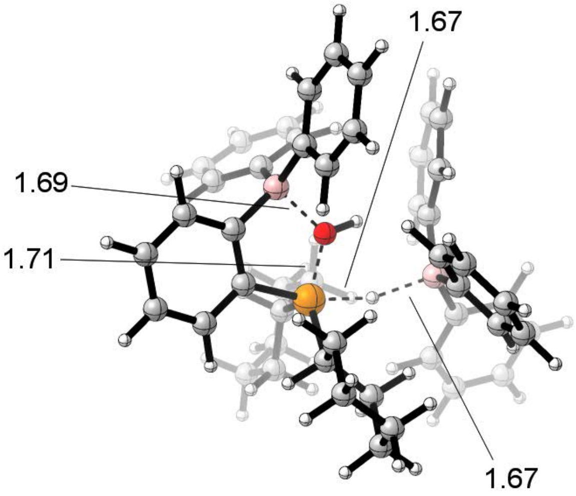

exo-4-H_2O-5-TS	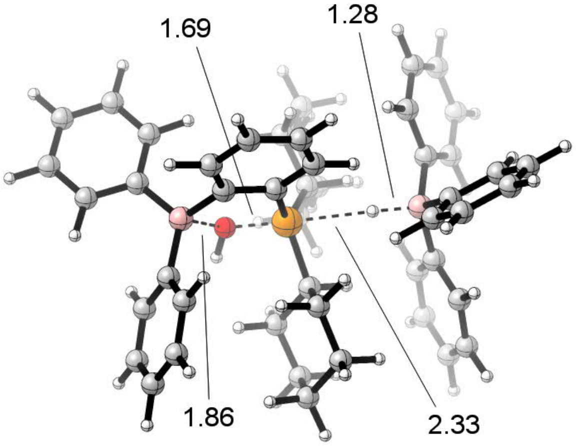
4-H_2O-4-TS	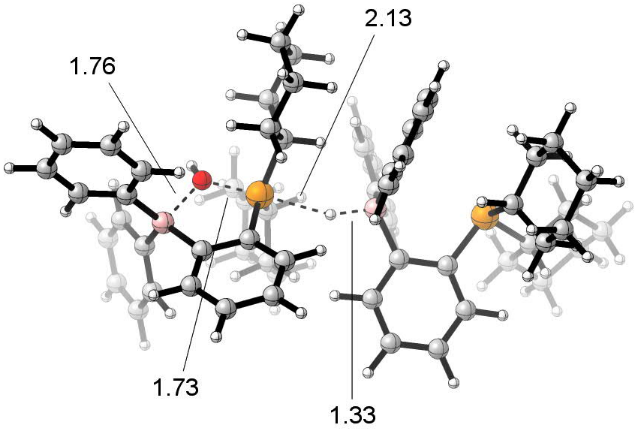
[5-H][4-OH]-TS	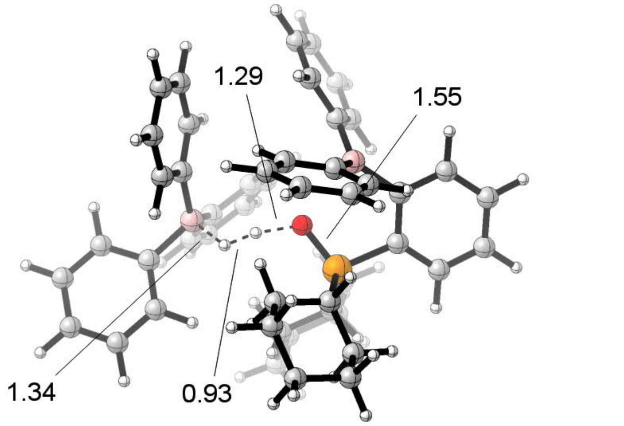

[4-H][4-OH]-TS	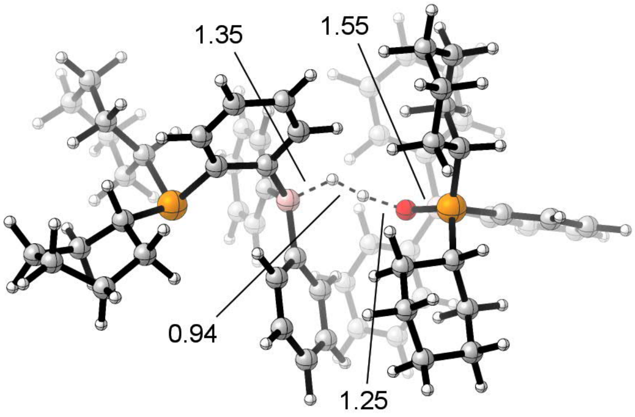
[4-H][4-OH]-IP	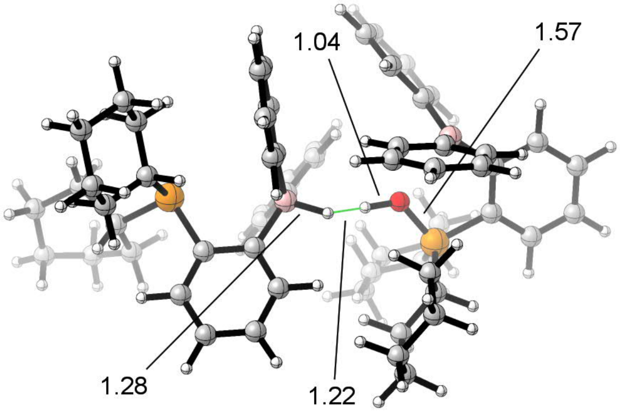
4c-TS	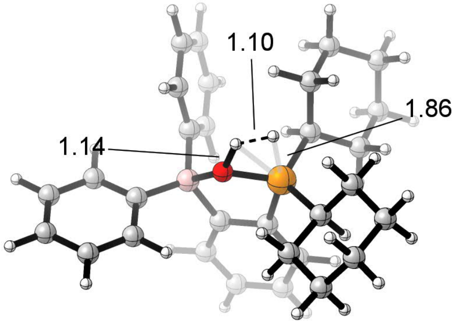
4-H_2O-upsd-TS1	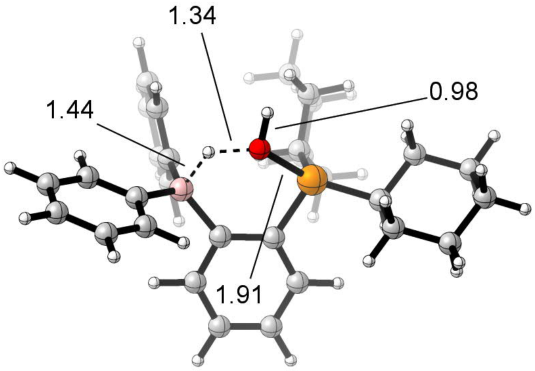

<table>
<tr><td>4-H_2O-upsd</td><td>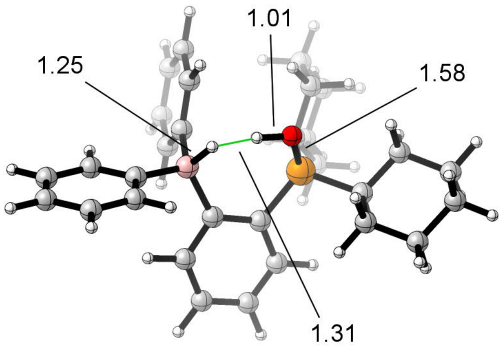
</td></tr>
<tr><td>4-H_2O-upsd-TS2</td><td>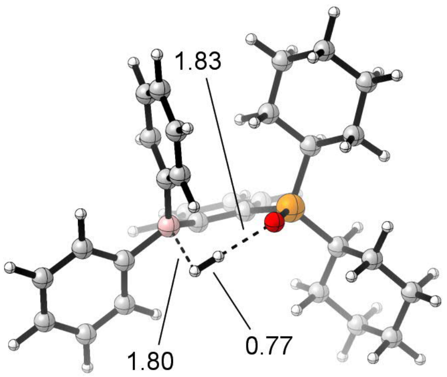
</td></tr>
<tr><td>endo-4-H_2</td><td>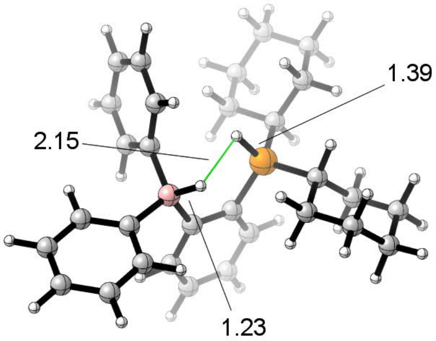
</td></tr>
<tr><td>exo-4-H_2</td><td>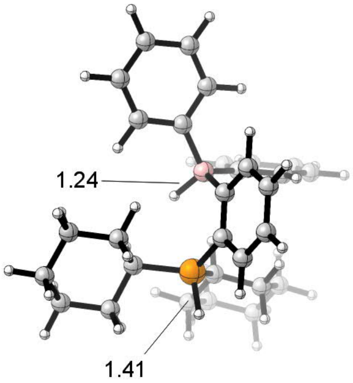
</td></tr>
</table>

Tables of energies

Table S3. Energies for geometries identified in the present study.

	ωB97XD/6-311G**, hartree					ωB97XD/6-311++G(3df,3pd)						
	Electronic energy	Gibbs free energy	Solution phase energy				Solution phase Gibbs free energy, kcal/mol			Relative Gibbs free energy, kcal/mol[a]		
			in DCM	in acetonitrile	in water	Electronic energy, hartree	in DCM	in acetonitrile	in water	in DCM	in acetonitrile	in water
H_2	-1.176086901	-1.174457	-1.175825727	-1.175648695	-1.173494535	-1.176595491	-737.1270206	-737.0159332	-737.0159332	0.0	0.0	0.0
H_2O	-76.42335327	-76.416893	-76.43068148	-76.43130479	-76.43661331	-76.43831253	-47965.58574	-47965.97687	-47965.97687	0.0	0.0	0.0
4	-1531.0483231	-1530.5121530	-1531.0816670	-1531.0777723	-1531.0533721	-1531.1449093	-960477.9072	-960475.4632	-960460.1521	4.8	5.1	6.1
4-O	-1606.330009	-1605.79029	-1606.370879	-1606.368455	-1606.345712	-1606.447021	-1007732.478	-1007730.957	-1007716.686	-21.3	-21.4	-21.5
4-H_2O-endo'	-1607.495456	-1606.935748	-1607.537453	-1607.535196	-1607.51482	-1607.602941	-1008445.981	-1008444.566	-1008431.779	2.4	2.0	0.5
4-H_2O-upsd	-1607.486157	-1606.925685	-1607.526552	-1607.524321	-1607.501248	-1607.602136	-1008443.992	-1008442.592	-1008428.114	4.4	4.0	4.1
4-H_2O-upsd-TS2	-1607.466816	-1606.911976	-1607.502947	-1607.499462	-1607.4801	-1607.584269	-1008433.639	-1008431.452	-1008419.302	14.7	15.1	12.9
4-H_2O-endo	-1607.4971294	-1606.9372250	-1607.5396585	-1607.5373297	-1607.5156542	-1607.6044546	-1008447.142	-1008445.681	-1008432.08	1.2	0.9	0.2
4c-TS	-1607.423685	-1606.869451	-1607.461048	-1607.457864	-1607.433358	-1607.538169	-1008405.865	-1008403.867	-1008388.489	42.5	42.7	43.8
4-H_2O-upsd-TS1	-1607.351571	-1606.796793	-1607.389672	-1607.386756	-1607.36398	-1607.457996	-1008355.678	-1008353.848	-1008339.557	92.7	92.7	92.7
4-H_2O-exo	-1607.503088	-1606.942105	-1607.543043	-1607.54022	-1607.517393	-1607.610019	-1008448.342	-1008446.571	-1008432.247	0.0	0.0	0.0
4-H_2O-exo'	-1607.5006317	-1606.9387220	-1607.5400312	-1607.5372617	-1607.5141305	-1607.6079525	-1008446.115	-1008444.377	-1008429.862	2.2	2.2	2.4
[4-H]+	-1531.453022	-1530.905874	-1531.540052	-1531.541329	-1531.517421	-1531.553406	-960761.0382	-960761.8395	-960746.8374			
[4-H]+ + [4-OH]-										24.1	17.9	14.9
[4-OH]-	-1606.968685	-1606.420004	-1607.050865	-1607.053235	-1607.036238	-1607.073643	-1008145.982	-1008147.469	-1008136.803			
[4-OH]+	-1606.70622	-1606.153378	-1606.791348	-1606.792144	-1606.771142	-1606.821278	-1007986.861	-1007987.361	-1007974.182			
[4-H]-	-1531.701058	-1531.15673	-1531.787034	-1531.79083	-1531.768255	-1531.799213	-960916.3895	-960918.7714	-960904.606			
[4-H]- + [4-OH]+										27.8	21.0	19.7
endo-4-H_2O-5-TS	-2327.216853	-2326.392642	-2327.267652	-2327.263379	-2327.232033	-2327.382662	-1459947.304	-1459944.623	-1459924.953	36.5	35.6	30.9
exo-4-H_2O-5-TS	-2327.232221	-2326.409195	-2327.290814	-2327.287161	-2327.256467	-2327.398348	-1459962.782	-1459960.489	-1459941.229	21.1	19.8	14.7
5	-719.7388542	-719.499976	-719.7614726	-719.7585581	-719.7425688	-719.7947507	-451535.5031	-451533.6742	-451523.6409	0.0	0.0	0.0
[5-H]-	-720.3896419	-720.147815	-720.4714329	-720.4756858	-720.4601902	-720.4492958	-451981.5106	-451984.1793	-451974.4559			
[5-H]- + [4-OH]+										15.5	8.7	7.2
[5-H][4-OH]-TS	-2327.242042	-2326.425018	-2327.29781	-2327.294466	-2327.262482	-2327.411969	-1459973.323	-1459971.224	-1459951.154	10.5	9.0	4.7
4-H_2O-4-TS	-3138.540811	-3137.417343	-3138.606	-3138.601239	-3138.563067	-3138.745544	-1968898.759	-1968895.771	-1968871.818	32.3	31.4	26.7
4-H_2O-TS	-1607.48811	-1606.931123	-1607.526882	-1607.523824	-1607.501643	-1607.593082	-1008439.479	-1008437.561	-1008423.642	8.9	9.0	8.6
4-H_2O-LA	-1607.496253	-1606.934021	-1607.533189	-1607.52954	-1607.509046	-1607.599468	-1008439.043	-1008436.753	-1008423.893	9.3	9.8	8.4
[4-H][4-OH]-TS	-3138.539623	-3137.422144	-3138.604616	-3138.601247	-3138.562513	-3138.748563	-1968904.288	-1968902.174	-1968877.869	26.8	25.0	20.6
[4-H][4-OH]-IP	-3138.541888	-3137.421832	-3138.611715	-3138.609423	-3138.571327	-3138.750925	-1968907.186	-1968905.748	-1968881.843	23.9	21.4	16.7
4-H_2-endo	-1532.2236108	-1531.6692340	-1532.2706370	-1532.2693441	-1532.2445826	-1532.3253900	-961215.8197	-961215.0084	-961199.4705	4.1	2.6	3.8
4-H_2-exo	-1532.2297298	-1531.6735420	-1532.2728713	-1532.2711779	-1532.2467016	-1532.3313858	-961216.0081	-961214.9455	-961199.5866	3.9	2.7	3.7
1	-3369.464554	-3368.973592	-3369.501281	-3369.497608	-3369.471547	-3369.589022	-2114132.079	-2114129.775	-2114113.421	0.0	0.0	0.0
1-H_2-endo	-3370.653181	-3370.142745	-3370.702106	-3370.700562	-3370.674245	-3370.780911	-2114875.423	-2114874.455	-2114857.941	-6.2	-7.7	-7.5
1-H_2-endo'	-3370.651895	-3370.140635	-3370.699941	-3370.698618	-3370.672788	-3370.779529	-2114873.487	-2114872.658	-2114856.449	-4.3	-5.9	-6.0
1-H_2-exo	-3370.657601	-3370.14594	-3370.700812	-3370.698802	-3370.673356	-3370.785106	-2114873.702	-2114872.441	-2114856.473	-4.5	-5.7	-6.0
1-H_2O-endo	-3445.9137395	-3445.3979720	-3445.9586075	-3445.9559851	-3445.9307904	-3446.0478191	-2162099.517	-2162097.871	-2162082.062	-1.9	-2.1	-2.7
1-H_2O-exo	-3445.924091	-3445.405904	-3445.963946	-3445.961062	-3445.936071	-3446.057517	-2162100.939	-2162099.129	-2162083.447	-3.3	-3.4	-4.0
1-H_2-TS	-3370.623594	-3370.116675	-3370.659686	-3370.655809	-3370.629231	-3370.748395	-2114849.173	-2114846.741	-2114830.063	20.0	20.0	20.4
1-H_2-TS'	-3370.620126	-3370.114326	-3370.656251	-3370.652389	-3370.625881	-3370.745055	-2114847.8	-2114845.377	-2114828.743	21.4	21.4	21.7

1-H_2O-TS	-3445.902908	-3445.389203	-3445.943699	-3445.940674	-3445.915471	-3446.034988	-2162090.201	-2162088.303	-2162072.488	7.5	7.4	6.9
1-H_2O-LA	-3445.9099	-3445.391366	-3445.947563	-3445.943909	-3445.920011	-3446.040782	-2162088.844	-2162086.552	-2162071.555	8.8	9.2	7.8

[a] Derivatives of 1 are reported relative to 1. Derivatives of 4 are reported relative to 4-H_2O-exo.

XYZ coordinates

2
H_2
H -0.208229 0.347334 0.000000
H -0.952676 0.347334 0.000000

3
H_2O
O 0.612750 0.314563 0.000000
H 1.568930 0.359482 0.000000
H 0.335914 1.230890 0.000000

70
1-H_2-endo'
P 2.907832 2.481500 2.487098
B 0.258119 2.220832 4.242595
C 1.267164 2.386431 1.781845
C 1.190124 2.367457 0.381630
H 2.087392 2.455519 -0.226075
C -0.037206 2.224893 -0.234819
H -0.109691 2.209505 -1.315789
C -1.175280 2.091824 0.559441
H -2.145594 1.970510 0.089776
C -1.080297 2.112664 1.939511
H -1.983511 2.010172 2.531551
C 0.139953 2.268333 2.613848
C 0.810174 3.623324 4.900500
C 1.644285 3.595966 6.032491
C 2.231975 4.712815 6.613274
H 2.852513 4.597477 7.492543
C 2.005438 5.961253 6.058811
C 1.179447 6.069529 4.952265
H 0.955802 7.035667 4.517603
C 0.605563 4.924776 4.413157
C -1.053496 1.530413 4.936040
C -2.042982 2.134859 5.724494
C -3.104056 1.456822 6.315240
H -3.818007 2.005773 6.915430
C -3.234722 0.093975 6.125157
C -2.305125 -0.570849 5.343103
H -2.380737 -1.636303 5.168912
C -1.261592 0.147392 4.780272
C 3.904494 1.054472 1.940143
H 4.014526 1.156614 0.851965
C 3.143385 -0.245871 2.249415
H 2.927253 -0.290418 3.323776
H 2.176128 -0.244506 1.742177
C 3.965765 -1.467253 1.838086
H 3.419734 -2.375212 2.105887
H 4.079077 -1.476542 0.746512
C 5.347717 -1.461459 2.491560

H 5.233140 -1.555550 3.578384
H 5.928956 -2.325727 2.159111
C 6.100916 -0.169106 2.175276
H 6.302423 -0.118946 1.097843
H 7.071337 -0.156860 2.678764
C 5.296191 1.064755 2.592196
H 5.844704 1.973893 2.327270
H 5.179381 1.065595 3.683577
C 3.815909 4.046784 2.157860
H 4.613366 3.786048 1.449187
C 2.934147 5.135681 1.529106
H 2.529575 4.797264 0.572635
H 2.074896 5.324253 2.177144
C 3.725764 6.430738 1.341903
H 4.524380 6.269168 0.605898
H 3.065241 7.196548 0.926985
C 4.339048 6.909866 2.657497
H 3.534467 7.150940 3.362283
H 4.912181 7.827137 2.497091
C 5.229011 5.829479 3.270959
H 5.630385 6.163238 4.231651
H 6.088853 5.646126 2.613579
C 4.453878 4.528409 3.475256
H 3.659086 4.695391 4.209868
H 5.110590 3.755774 3.887733
H 2.819530 2.358762 3.874518
H 1.144556 1.421356 4.455570
Cl -0.157368 -0.821710 3.795366
Cl -2.036424 3.869428 6.037055
Cl 1.986077 2.069878 6.843646
Cl -0.517762 5.225234 3.090861
H -4.056093 -0.446961 6.579783
H 2.453252 6.845439 6.496766

70
1-H_2-endo
P 3.067594 2.351168 2.576801
B 0.417034 2.467890 4.308592
C 1.432930 2.293302 1.854952
C 1.348423 2.171896 0.461374
H 2.244237 2.086966 -0.147398
C 0.108520 2.141464 -0.148185
H 0.029930 2.046268 -1.224666
C -1.033411 2.222970 0.645844
H -2.013135 2.191575 0.181160
C -0.930965 2.342259 2.021336
H -1.837161 2.404655 2.614214
C 0.302608 2.396115 2.685618
C 1.109844 3.832013 4.911232
C 1.786085 3.770805 6.144435
C 2.480842 4.825400 6.721970
H 2.963684 4.688829 7.681012
C 2.535583 6.041960 6.062374
C 1.880900 6.182902 4.851059
H 1.880308 7.129833 4.326116
C 1.193820 5.100112 4.314978
C -0.963817 1.977241 5.027383
C -1.951479 2.778737 5.613140
C -3.080415 2.291040 6.261273
H -3.787915 2.984046 6.697883
C -3.285559 0.925071 6.330638

C -2.371274 0.070036 5.736974
H -2.515081 -1.002426 5.757845
C -1.258186 0.605240 5.106968
C 4.082894 0.972867 1.943133
H 4.192539 1.130075 0.862669
C 3.322069 -0.345370 2.185063
H 3.115214 -0.449387 3.257666
H 2.351666 -0.319266 1.684824
C 4.141783 -1.543090 1.704355
H 3.595064 -2.464102 1.921416
H 4.252342 -1.490656 0.613833
C 5.524931 -1.575291 2.353895
H 5.412973 -1.728726 3.434254
H 6.103893 -2.420882 1.973152
C 6.277702 -0.268543 2.106126
H 6.476736 -0.161145 1.032390
H 7.249496 -0.284017 2.606770
C 5.477445 0.943770 2.589169
H 6.027241 1.861219 2.364617
H 5.368258 0.894436 3.680004
C 3.835879 3.994439 2.350719
H 2.969578 4.664804 2.373574
C 4.754528 4.375901 3.521428
H 4.202264 4.305323 4.462640
H 5.600984 3.682129 3.583402
C 5.279208 5.799523 3.330407
H 4.434505 6.494897 3.398547
H 5.960955 6.050595 4.147366
C 5.973796 5.967408 1.978332
H 6.869140 5.332812 1.948578
H 6.315727 6.998531 1.855272
C 5.044891 5.582742 0.825675
H 5.562735 5.677927 -0.132582
H 4.193981 6.273696 0.797918
C 4.516980 4.154510 0.984170
H 5.352410 3.449461 0.894383
H 3.810987 3.927997 0.179382
H 2.962136 2.132688 3.953792
H 1.215453 1.603078 4.597146
Cl -0.194191 -0.586199 4.347667
Cl -1.847792 4.536268 5.534193
Cl 1.764522 2.286010 7.089069
Cl 0.347008 5.455983 2.807952
H -4.158812 0.527676 6.834156
H 3.070245 6.878218 6.496782

70
1-H_2-exo
P 4.276840 2.402434 3.278902
H 5.624664 2.028183 3.108520
B 1.124408 2.517180 2.671613
C 3.545572 2.035930 1.681555
C 2.162018 2.179797 1.459099
C 1.761412 2.066558 0.121534
H 0.714771 2.200405 -0.122115
C 2.644967 1.765195 -0.902844
H 2.276668 1.677786 -1.919394
C 3.995211 1.563609 -0.637642
H 4.686774 1.311781 -1.432835
C 4.449111 1.714394 0.659996
H 5.506605 1.593592 0.872993

C -0.132969 3.436618 2.146937
C -1.296517 2.925274 1.549958
Cl -1.473113 1.195027 1.254638
C -2.375792 3.692208 1.129892
H -3.235653 3.207693 0.685855
C -2.332334 5.065724 1.284821
H -3.165609 5.680609 0.966531
C -1.209319 5.645973 1.848353
H -1.141880 6.717995 1.979589
C -0.159064 4.834812 2.252731
Cl 1.201784 5.720295 2.952296
C 0.577392 1.258738 3.593925
C 0.678115 -0.122065 3.361154
Cl 1.467691 -0.758990 1.918937
C 0.184972 -1.104175 4.211679
H 0.292758 -2.146510 3.940191
C -0.438049 -0.737879 5.391396
H -0.832864 -1.492640 6.060858
C -0.545939 0.605636 5.705941
H -1.017699 0.928280 6.625179
C -0.039231 1.551229 4.825025
Cl -0.188189 3.219159 5.372361
C 3.677596 1.358676 4.648092
H 2.611574 1.262314 4.443526
C 3.817594 1.952024 6.053186
H 3.346832 2.938203 6.098449
H 4.876963 2.069062 6.313673
C 3.155107 1.010650 7.062978
H 3.258619 1.423477 8.070134
H 2.082093 0.958297 6.845149
C 3.760766 -0.392163 6.994292
H 3.254045 -1.053851 7.702072
H 4.813721 -0.346564 7.302513
C 3.669272 -0.971334 5.581806
H 2.616915 -1.120930 5.319083
H 4.153438 -1.950796 5.536984
C 4.304793 -0.040212 4.546283
H 5.386437 0.027839 4.724723
H 4.159330 -0.448449 3.541221
C 4.410726 4.200463 3.615504
H 3.469832 4.496119 4.088945
C 5.592580 4.495935 4.555660
H 5.467144 3.990234 5.514284
H 6.518050 4.111782 4.106489
C 5.740128 6.002368 4.783499
H 4.849321 6.375152 5.303264
H 6.592491 6.186917 5.443280
C 5.909078 6.750371 3.462406
H 5.986669 7.825722 3.643977
H 6.850001 6.440143 2.989889
C 4.743722 6.456189 2.519394
H 4.891178 6.954608 1.557728
H 3.820805 6.865390 2.944639
C 4.561108 4.956266 2.283178
H 5.433368 4.566753 1.740432
H 3.687414 4.778042 1.654337
H 1.770572 3.184268 3.461191

71
1-H_2O-endo
C 0.4518480 -10.2164790 11.1401060

C	-0.8770140	-10.5045190	11.4632040
C	-1.9413960	-9.8340950	10.8819720
C	-1.7147850	-8.8360230	9.9394300
C	-0.4126810	-8.5197800	9.5914230
C	0.6519100	-9.1994610	10.1940520
B	1.7455450	-10.9164510	11.8679130
H	-1.0770910	-11.2834250	12.1915360
H	-2.9577420	-10.0881000	11.1632180
H	-2.5435600	-8.3085780	9.4819030
H	-0.2369340	-7.7466700	8.8499940
P	2.3187230	-8.6990180	9.7278140
C	0.4969480	-12.5044820	15.9240990
C	0.6192110	-11.1453390	15.6962310
C	0.9772910	-10.7071880	14.4306060
C	1.2645040	-11.5330070	13.3320190
C	1.0525090	-12.8903760	13.6236880
C	0.6976220	-13.3814980	14.8746500
H	0.2253960	-12.8742770	16.9054270
H	0.4319020	-10.4264880	16.4830470
Cl	0.9886470	-8.9317840	14.2786500
Cl	1.1649600	-14.1280310	12.3773290
H	0.5746400	-14.4480040	15.0105060
C	4.4093480	-13.3060280	9.1243620
C	3.0927370	-13.1010170	8.7534750
C	2.2244290	-12.4668530	9.6359150
C	2.5943650	-11.9689700	10.8932380
C	3.9225620	-12.2852780	11.2340350
C	4.8214150	-12.9200610	10.3893320
H	5.0994300	-13.7972190	8.4486490
H	2.7228240	-13.4469450	7.7966320
Cl	0.5494720	-12.4241030	9.0825980
Cl	4.5392960	-11.9600380	12.8504380
H	5.8288130	-13.1188460	10.7317560
C	1.8589020	-9.3052550	5.6405760
C	1.7980740	-9.5437040	7.1493710
C	2.3371090	-8.3304790	7.9289950
C	3.7627190	-7.9808420	7.4654870
C	3.8140090	-7.7513400	5.9530580
C	3.2741610	-8.9555510	5.1830360
H	1.1782010	-8.4857960	5.3764520
H	1.4964850	-10.1952180	5.1194170
H	2.4015540	-10.4254560	7.4014070
H	0.7763710	-9.7738630	7.4556690
H	1.6877450	-7.4663320	7.7457750
H	4.1346140	-7.0926950	7.9817370
H	4.4371680	-8.8065190	7.7275510
H	3.2176810	-6.8640780	5.7052010
H	4.8427580	-7.5333500	5.6531390
H	3.9315160	-9.8171360	5.3541670
H	3.2892830	-8.7544930	4.1083100
C	2.7757440	-4.7504800	11.0131910
C	2.3837240	-5.9077260	10.0900530
C	2.8646830	-7.2369020	10.6878770
C	4.3742850	-7.2489810	10.9669230
C	4.7476150	-6.0756070	11.8750200
C	4.2802480	-4.7415020	11.2902020
H	2.2322270	-4.8496090	11.9605040
H	2.4630870	-3.8024430	10.5668880
H	2.8341970	-5.7451040	9.1043610
H	1.2977570	-5.9239450	9.9518420
H	2.3464300	-7.3836420	11.6394220

H	4.6446040	-8.1998950	11.4315560
H	4.9335060	-7.1690280	10.0276200
H	4.2838560	-6.2241690	12.8579400
H	5.8288720	-6.0647350	12.0360930
H	4.8181110	-4.5499670	10.3525380
H	4.5315100	-3.9221390	11.9692060
H	3.2557040	-9.7296990	9.8072830
O	2.7465920	-9.8047120	12.0082040
H	2.6621550	-9.3571760	12.8482840

71

1-H_2O-exo

P 4.152670 2.479290 3.291494
H 5.470484 2.023569 3.036779
O 1.958430 3.295839 3.540050
H 1.526548 3.670275 4.308206
B 1.027631 2.553689 2.640527
C 3.436307 2.166792 1.663547
C 2.052517 2.205471 1.416714
C 1.660272 2.027804 0.087936
H 0.608603 2.079051 -0.163094
C 2.570010 1.775698 -0.927825
H 2.215722 1.639332 -1.943879
C 3.928889 1.694641 -0.651038
H 4.644794 1.488178 -1.437945
C 4.361301 1.896265 0.648575
H 5.422949 1.849325 0.868366
C -0.242510 3.459817 2.060819
C -1.373797 2.872468 1.467291
Cl -1.501970 1.121284 1.270112
C -2.465408 3.570135 0.967797
H -3.294121 3.025345 0.534754
C -2.473534 4.950715 1.029827
H -3.316427 5.514405 0.648648
C -1.385347 5.602054 1.580400
H -1.354525 6.682189 1.637965
C -0.316521 4.862064 2.068213
Cl 0.974157 5.880610 2.729770
C 0.443701 1.282293 3.574444
C 0.674259 -0.094207 3.420170
Cl 1.588134 -0.731442 2.054953
C 0.222718 -1.073350 4.296556
H 0.441448 -2.113153 4.090130
C -0.505366 -0.710180 5.415450
H -0.869897 -1.464099 6.102544
C -0.760991 0.630084 5.643436
H -1.323102 0.953177 6.509976
C -0.280691 1.569609 4.742480
Cl -0.649819 3.245781 5.190253
C 3.651764 1.332077 4.628059
H 2.646776 1.032861 4.335896
C 3.543586 1.962192 6.019856
H 2.910714 2.851932 5.989446
H 4.533420 2.273979 6.375622
C 2.954026 0.936950 6.992330
H 2.886328 1.372900 7.993022
H 1.931128 0.701075 6.674878
C 3.788113 -0.345176 7.024641
H 3.325824 -1.076360 7.693706
H 4.779354 -0.120125 7.439677
C 3.952066 -0.943993 5.626330

H 2.974090 -1.272141 5.255005
H 4.593815 -1.828741 5.662665
C 4.530837 0.075983 4.642500
H 5.554997 0.339574 4.937620
H 4.581273 -0.360437 3.640009
C 4.550975 4.213248 3.742192
H 3.765670 4.545056 4.423297
C 5.912320 4.309697 4.444906
H 5.937257 3.680803 5.340074
H 6.697631 3.940154 3.773687
C 6.212848 5.764621 4.816363
H 5.476495 6.105541 5.554548
H 7.193430 5.826807 5.296703
C 6.160178 6.675158 3.588211
H 6.336553 7.713576 3.882352
H 6.971632 6.399060 2.902728
C 4.821998 6.549732 2.857940
H 4.820194 7.165704 1.954784
H 4.017907 6.930823 3.499565
C 4.513502 5.098638 2.488779
H 5.256941 4.739018 1.765021
H 3.534791 5.021932 2.013620

71
1-H_2O-LA
P 3.221783 2.159822 2.887702
B 0.360409 2.508262 4.441929
C 1.581627 2.142640 2.055157
C 1.502771 1.995420 0.666945
H 2.408051 1.869126 0.082703
C 0.281396 1.992533 0.013460
H 0.239472 1.877335 -1.063890
C -0.882757 2.128049 0.757678
H -1.849416 2.120079 0.266116
C -0.809077 2.267838 2.133568
H -1.732141 2.375737 2.692779
C 0.407820 2.288981 2.825785
C 0.988878 3.950472 4.983876
C 1.497560 4.096943 6.285393
C 2.189713 5.197561 6.763610
H 2.551004 5.208178 7.783391
C 2.386896 6.277716 5.922111
C 1.839356 6.246466 4.653022
H 1.924891 7.099365 3.992261
C 1.153130 5.119171 4.218463
C -1.073164 2.044399 5.091876
C -2.022142 2.897451 5.677511
C -3.194746 2.464527 6.284347
H -3.865509 3.191051 6.723490
C -3.491184 1.115050 6.309453
C -2.632751 0.222720 5.692521
H -2.859063 -0.834866 5.658682
C -1.472414 0.692174 5.092527
C 4.235269 0.910682 1.971225
H 4.306843 1.203956 0.916636
C 3.561003 -0.467998 2.049341
H 3.431981 -0.743737 3.104472
H 2.559681 -0.426026 1.614112
C 4.399473 -1.537048 1.346222
H 3.911336 -2.511130 1.442336
H 4.442683 -1.310932 0.273089

C 5.820633 -1.595464 1.907216
H 5.780660 -1.925779 2.953082
H 6.414362 -2.337049 1.364837
C 6.495175 -0.225074 1.841099
H 6.631440 0.061330 0.790358
H 7.493071 -0.268655 2.287405
C 5.658768 0.845307 2.546386
H 6.155104 1.816048 2.461619
H 5.601051 0.614801 3.618137
C 3.908868 3.840387 2.481222
H 3.016446 4.473476 2.496632
C 4.841059 4.342608 3.593995
H 4.320756 4.289989 4.555575
H 5.719994 3.690152 3.671170
C 5.298364 5.777374 3.324604
H 4.427151 6.440989 3.386833
H 5.996330 6.098716 4.103678
C 5.936019 5.916446 1.941973
H 6.857274 5.320184 1.906088
H 6.226538 6.955515 1.760984
C 4.982920 5.434428 0.847799
H 5.456328 5.516620 -0.135185
H 4.096624 6.080921 0.828609
C 4.539411 3.989483 1.092024
H 5.411810 3.331329 0.999451
H 3.824382 3.687359 0.321710
H 2.303999 1.600615 4.573482
O 1.409931 1.416604 5.015144
H 1.492269 1.467227 5.979051
H -4.398634 0.762385 6.784686
H 2.931507 7.148998 6.264476
Cl -1.842362 4.647770 5.643258
Cl -0.547466 -0.552419 4.255065
Cl 1.182609 2.862275 7.529781
Cl 0.393690 5.313273 2.647338

71
1-H_2O-TS
P 3.150367 2.238862 2.708107
B 0.454884 2.477597 4.402835
C 1.516312 2.217348 1.928557
C 1.397894 2.112742 0.539918
H 2.285267 2.025202 -0.080005
C 0.148729 2.102895 -0.056237
H 0.056427 2.019787 -1.133116
C -0.981986 2.188002 0.748632
H -1.968760 2.171200 0.298740
C -0.855859 2.291657 2.124324
H -1.755715 2.362202 2.725590
C 0.388077 2.322249 2.766145
C 1.004537 3.975749 4.925820
C 1.527700 4.128446 6.222591
C 2.191598 5.247782 6.699096
H 2.561263 5.263157 7.715989
C 2.350715 6.339253 5.863215
C 1.793933 6.298054 4.598745
H 1.849042 7.155911 3.940832
C 1.133660 5.152398 4.168073
C -0.956135 1.962898 5.082292
C -1.937634 2.771402 5.675024
C -3.074155 2.288810 6.312642

H -3.771328 2.986049 6.758358
C -3.301651 0.926341 6.358251
C -2.412844 0.072113 5.729740
H -2.586961 -0.995758 5.708565
C -1.290085 0.594695 5.102831
C 4.170794 0.922589 1.933300
H 4.297270 1.171294 0.871835
C 3.433073 -0.423157 2.043641
H 3.223220 -0.631841 3.100165
H 2.463953 -0.364501 1.543179
C 4.270365 -1.558478 1.453048
H 3.737997 -2.505731 1.574264
H 4.385562 -1.397351 0.373549
C 5.652283 -1.635841 2.102440
H 5.538150 -1.899443 3.161427
H 6.245565 -2.430574 1.641536
C 6.385777 -0.299003 1.994612
H 6.590494 -0.081300 0.938636
H 7.354776 -0.351818 2.499026
C 5.558852 0.843411 2.589094
H 6.096803 1.788321 2.473489
H 5.435916 0.679086 3.667339
C 3.898820 3.898119 2.422824
H 3.023465 4.555584 2.409836
C 4.784550 4.332784 3.600476
H 4.208858 4.281893 4.529827
H 5.633741 3.646612 3.707783
C 5.305776 5.754928 3.386883
H 4.456202 6.447450 3.418953
H 5.970150 6.032472 4.210242
C 6.024323 5.897531 2.044660
H 6.923659 5.267740 2.045318
H 6.362574 6.928089 1.904542
C 5.117595 5.478320 0.886609
H 5.649129 5.561736 -0.065687
H 4.259415 6.159291 0.832690
C 4.605797 4.047411 1.069791
H 5.453940 3.354803 1.008318
H 3.919253 3.790087 0.257595
H 2.613247 1.855092 4.155843
O 1.564052 1.503877 4.849330
H 1.647204 1.459428 5.806124
H -4.179685 0.533585 6.856887
H 2.871616 7.224635 6.206792
Cl -1.848536 4.529952 5.608446
Cl -0.323631 -0.608631 4.246883
Cl 1.273095 2.876117 7.456205
Cl 0.361992 5.338523 2.596189

70
1-H_2-TS'
P 2.965119 2.559586 2.917744
B -0.028223 2.383159 4.286999
C 1.362014 2.427640 2.001303
C 1.354305 2.390118 0.605197
H 2.286689 2.505553 0.061329
C 0.178755 2.217911 -0.109410
H 0.197839 2.207361 -1.193599
C -1.016605 2.048552 0.576011
H -1.943149 1.899623 0.032964
C -1.019699 2.063874 1.961195

H -1.961703 1.928738 2.482642
C 0.149102 2.270475 2.705509
C 0.557627 3.671259 5.050836
C 1.184729 3.613079 6.304696
C 1.842554 4.685896 6.887614
H 2.328113 4.559182 7.846103
C 1.850273 5.908642 6.239597
C 1.161846 6.055328 5.047071
H 1.100011 7.016683 4.553689
C 0.527288 4.956775 4.486860
C -1.223343 1.527262 4.945589
C -2.273818 2.114879 5.670678
C -3.315601 1.393608 6.237449
H -4.089697 1.917269 6.782438
C -3.351639 0.018500 6.093773
C -2.361948 -0.619240 5.366899
H -2.375190 -1.691880 5.226660
C -1.341403 0.132382 4.804278
C 3.902328 1.161015 2.106490
H 3.896251 1.320795 1.019507
C 3.206441 -0.174974 2.400925
H 3.140936 -0.311916 3.488578
H 2.180911 -0.152676 2.022909
C 3.963990 -1.356026 1.792212
H 3.457494 -2.291791 2.046982
H 3.941209 -1.272678 0.698048
C 5.417011 -1.389206 2.265490
H 5.438701 -1.571153 3.347632
H 5.953772 -2.218339 1.794859
C 6.118505 -0.064703 1.965405
H 6.184291 0.069241 0.877953
H 7.145432 -0.081864 2.342835
C 5.365018 1.123213 2.570483
H 5.876412 2.051443 2.299013
H 5.393378 1.053052 3.665901
C 3.844108 4.054837 2.201293
H 4.426952 3.703608 1.337522
C 2.929384 5.189482 1.727384
H 2.229037 4.839327 0.967408
H 2.325175 5.533425 2.571496
C 3.742599 6.368926 1.189064
H 4.295205 6.050242 0.295468
H 3.064846 7.167102 0.871373
C 4.730131 6.893179 2.231742
H 4.168432 7.307874 3.078934
H 5.327170 7.710806 1.816706
C 5.637304 5.771980 2.738496
H 6.302173 6.144082 3.523993
H 6.280280 5.427001 1.918170
C 4.817292 4.593561 3.266084
H 4.228385 4.915390 4.134807
H 5.481807 3.801865 3.623667
H 1.615005 1.559879 4.555016
H 1.091696 1.136210 4.920287
Cl -0.161616 -0.791096 3.886937
Cl -2.385724 3.852883 5.873116
Cl 1.138407 2.146189 7.270637
Cl -0.443856 5.291286 3.065662
H -4.156260 -0.554790 6.538073
H 2.360896 6.755783 6.681372

70

1-H_2-TS

P 3.170876 2.251358 2.949212
B 0.206375 2.705223 4.315816
C 1.554919 2.241445 2.046682
C 1.503226 2.029200 0.666987
H 2.419429 1.867594 0.111055
C 0.300028 2.011892 -0.023314
H 0.293736 1.857809 -1.096740
C -0.888633 2.182011 0.670797
H -1.838223 2.162677 0.147992
C -0.855494 2.358975 2.044248
H -1.793595 2.475093 2.576924
C 0.347399 2.406633 2.761779
C 1.010666 3.918045 4.996723
C 1.549786 3.862259 6.292963
C 2.331397 4.866058 6.846376
H 2.733006 4.741698 7.843234
C 2.564989 6.021952 6.122651
C 1.989406 6.173584 4.873133
H 2.113273 7.089295 4.310156
C 1.232780 5.140876 4.340680
C -1.104622 2.114969 5.034131
C -2.045022 2.930707 5.684285
C -3.180035 2.444502 6.316884
H -3.859163 3.135298 6.798635
C -3.428592 1.083558 6.315651
C -2.561213 0.229052 5.658047
H -2.746905 -0.836246 5.624411
C -1.442339 0.748986 5.024036
C 4.158740 0.969660 2.031306
H 4.226745 1.228271 0.967402
C 3.472833 -0.399855 2.157398
H 3.354234 -0.640444 3.222309
H 2.466640 -0.361685 1.731724
C 4.288752 -1.501428 1.478147
H 3.790031 -2.466376 1.609052
H 4.323820 -1.308705 0.398059
C 5.714905 -1.560377 2.026108
H 5.681476 -1.856793 3.082286
H 6.294035 -2.326102 1.501395
C 6.404118 -0.200728 1.910209
H 6.532054 0.050399 0.849268
H 7.406621 -0.242946 2.346614
C 5.588419 0.901530 2.590440
H 6.095730 1.862214 2.466048
H 5.541899 0.708758 3.669953
C 3.881787 3.904666 2.438912
H 3.006256 4.561446 2.462215
C 4.861001 4.421758 3.503940
H 4.384564 4.382682 4.487678
H 5.740067 3.767126 3.554123
C 5.314023 5.848532 3.189999
H 4.446964 6.517702 3.261883
H 6.037085 6.186205 3.938974
C 5.910521 5.951316 1.785728
H 6.829707 5.352596 1.740255
H 6.196961 6.984691 1.568245
C 4.926966 5.441068 0.731884
H 5.376455 5.490488 -0.264691
H 4.044953 6.093753 0.714742

C 4.477650 4.007494 1.031168
H 5.341607 3.338137 0.937782
H 3.743344 3.691082 0.285555
H 1.754302 1.560609 4.689470
H 1.162925 1.304025 5.099140
Cl -0.446473 -0.433365 4.190033
Cl -1.883534 4.678000 5.685755
Cl 1.219954 2.505630 7.357505
Cl 0.453237 5.504762 2.810652
H -4.306755 0.690145 6.813233
H 3.171233 6.815231 6.542889

69
1-O
P 1.688135 0.359036 2.126463
B 0.572191 2.423652 3.226474
C -0.064916 0.398996 1.799672
C -0.862507 -0.478808 1.069094
H -0.437332 -1.343863 0.570150
C -2.224382 -0.235823 1.010169
H -2.873850 -0.903836 0.456506
C -2.758871 0.860664 1.688336
H -3.829400 1.032692 1.660186
C -1.944850 1.733263 2.393699
H -2.386925 2.584609 2.897995
C -0.562498 1.539232 2.444224
C 1.311691 3.677378 2.426540
C 2.437821 4.281457 3.015588
C 3.214184 5.258906 2.408492
H 4.060139 5.674977 2.940032
C 2.894500 5.685967 1.131750
C 1.803043 5.130600 0.489615
H 1.523177 5.446108 -0.507109
C 1.049529 4.157988 1.135933
C 0.010694 2.853014 4.707102
C -0.753745 4.019545 4.874844
C -1.261595 4.465591 6.086999
H -1.828028 5.386946 6.122093
C -1.036975 3.720814 7.229669
C -0.332090 2.534920 7.131213
H -0.158168 1.917157 8.002455
C 0.156214 2.126436 5.897666
C 2.356740 -1.244180 2.670209
H 2.691963 -1.754008 1.756744
C 1.286358 -2.111437 3.356400
H 0.856922 -1.555179 4.195213
H 0.464282 -2.316771 2.667257
C 1.886130 -3.424556 3.861266
H 1.112726 -3.999487 4.377689
H 2.210061 -4.029181 3.003949
C 3.079231 -3.181566 4.784635
H 2.738853 -2.653854 5.683916
H 3.501981 -4.134124 5.116010
C 4.146823 -2.342827 4.083727
H 4.552785 -2.907808 3.234529
H 4.982957 -2.144869 4.759852
C 3.576307 -1.013814 3.587239
H 4.349785 -0.447478 3.063323
H 3.264071 -0.400125 4.437087
C 2.581330 0.990905 0.672742
H 1.969241 1.860510 0.406676

C 3.993883 1.503276 0.981298
H 3.971985 2.169969 1.846349
H 4.650163 0.659516 1.227221
C 4.548598 2.229181 -0.245509
H 3.936990 3.120438 -0.430734
H 5.563969 2.579392 -0.040489
C 4.537230 1.322536 -1.478543
H 5.235117 0.490428 -1.317770
H 4.898409 1.869506 -2.353932
C 3.141541 0.758244 -1.755891
H 3.170874 0.072487 -2.607300
H 2.464841 1.576793 -2.028911
C 2.568609 0.039739 -0.529812
H 3.176788 -0.847443 -0.312183
H 1.550358 -0.302134 -0.741785
O 1.756702 1.395836 3.285207
H -1.418945 4.054052 8.187006
H 3.488878 6.448501 0.642876
Cl -1.171319 5.040462 3.496671
Cl 0.988881 0.571331 5.953461
Cl 2.977547 3.818143 4.624879
Cl -0.307625 3.560809 0.179818

68
1
P 3.017258 2.202891 2.992685
B 0.124195 2.879058 4.143123
C 1.477483 2.275758 1.964856
C 1.462203 2.019906 0.594504
H 2.384350 1.783516 0.076200
C 0.277217 2.041780 -0.131507
H 0.295231 1.847518 -1.198373
C -0.925694 2.290866 0.513167
H -1.856136 2.293830 -0.043067
C -0.930799 2.510966 1.882305
H -1.876425 2.680783 2.387690
C 0.257967 2.532743 2.623154
C 1.024197 3.959539 4.876417
C 1.677992 3.692316 6.086534
C 2.437394 4.631856 6.765433
H 2.928602 4.358069 7.689842
C 2.557839 5.909466 6.241918
C 1.930190 6.232216 5.050042
H 2.008864 7.226001 4.629285
C 1.187779 5.263714 4.391377
C -1.069373 2.196207 4.935463
C -2.105898 2.925965 5.523346
C -3.166642 2.336331 6.195160
H -3.943747 2.955498 6.623676
C -3.209920 0.955850 6.301740
C -2.211867 0.180628 5.730793
H -2.231802 -0.898658 5.805679
C -1.175334 0.805038 5.055989
C 4.020164 0.903678 2.111222
H 4.143438 1.166606 1.053200
C 3.303810 -0.452880 2.197460
H 3.135642 -0.695273 3.254940
H 2.317379 -0.393101 1.729284
C 4.126847 -1.566997 1.547987
H 3.603159 -2.522378 1.648862
H 4.216514 -1.368672 0.471969

C 5.524230 -1.658550 2.161712
H 5.434811 -1.960019 3.213176
H 6.111981 -2.433026 1.659763
C 6.245128 -0.312565 2.087305
H 6.428142 -0.058704 1.035060
H 7.224962 -0.377718 2.570100
C 5.420912 0.803105 2.734696
H 5.953011 1.753328 2.637407
H 5.320693 0.607804 3.810124
C 3.803905 3.834819 2.509306
H 2.946932 4.518398 2.508123
C 4.761851 4.327355 3.604416
H 4.261545 4.288054 4.575461
H 5.629448 3.659270 3.671773
C 5.244367 5.749348 3.313455
H 4.384366 6.429898 3.361803
H 5.948247 6.073654 4.086423
C 5.887396 5.848862 1.929423
H 6.799334 5.237661 1.912195
H 6.195030 6.878900 1.725062
C 4.932809 5.355421 0.841147
H 5.418013 5.398581 -0.138955
H 4.063217 6.023176 0.794222
C 4.448033 3.929723 1.122840
H 5.302160 3.244252 1.058419
H 3.734454 3.626943 0.351616
H -4.027944 0.480174 6.829272
H 3.145727 6.655471 6.762852
Cl 1.603025 2.089111 6.776992
Cl -2.127918 4.673301 5.376063
Cl 0.068192 -0.222238 4.376018
Cl 0.400226 5.763074 2.906563

71
4c-TS
P 1.965096 0.500778 2.393278
B 0.368975 2.662441 3.227526
C 0.203421 0.501255 1.875007
C -0.429746 -0.464266 1.092364
H 0.100242 -1.343441 0.739009
C -1.769368 -0.309775 0.762501
H -2.267854 -1.058260 0.157037
C -2.471462 0.801056 1.221556
H -3.521227 0.913446 0.972213
C -1.838806 1.754871 2.008355
H -2.399057 2.606002 2.383301
C -0.491557 1.624232 2.344299
C 1.053584 3.842583 2.361785
C 2.167134 4.529087 2.864554
C 2.843343 5.481367 2.112621
H 3.707986 5.987892 2.528741
C 2.409665 5.788003 0.826683
C 1.293724 5.140882 0.312146
H 0.940540 5.379015 -0.685908
C 0.630689 4.181590 1.072925
C -0.281730 3.072278 4.630893
C -0.583580 4.397136 4.955535
C -1.192674 4.730961 6.162417
H -1.417305 5.768611 6.386560
C -1.507247 3.738788 7.080561
C -1.215466 2.411110 6.781251

H -1.459865 1.628930 7.492712
C -0.617588 2.089382 5.571201
C 2.262916 -1.269064 2.784790
H 1.779904 -1.801556 1.955320
C 1.518711 -1.622543 4.082627
H 1.951188 -1.040252 4.905456
H 0.465778 -1.337579 4.006466
C 1.644428 -3.116774 4.386394
H 1.148179 -3.339672 5.334884
H 1.114402 -3.685108 3.611700
C 3.106617 -3.562442 4.431276
H 3.605907 -3.073061 5.276734
H 3.167736 -4.639830 4.608829
C 3.838248 -3.195513 3.139327
H 3.410096 -3.764516 2.304169
H 4.892183 -3.479428 3.205323
C 3.729653 -1.696297 2.847519
H 4.243792 -1.452669 1.913316
H 4.231701 -1.130389 3.638251
C 2.793317 1.193622 0.903652
H 2.100763 2.001187 0.635914
C 4.170166 1.820718 1.124548
H 4.119237 2.550022 1.935594
H 4.886345 1.048885 1.429077
C 4.642359 2.504063 -0.160004
H 3.968283 3.341097 -0.380009
H 5.636410 2.932127 -0.003040
C 4.662145 1.530125 -1.338715
H 5.412822 0.751357 -1.150944
H 4.966253 2.046217 -2.253817
C 3.296339 0.872521 -1.537453
H 3.336468 0.143326 -2.351857
H 2.564878 1.635954 -1.828841
C 2.806250 0.187024 -0.258048
H 3.471842 -0.651277 -0.018862
H 1.807903 -0.224259 -0.426274
H 3.494742 0.842217 3.400152
O 1.617021 1.621071 3.567415
H 2.735103 1.538925 3.796027
H -1.977796 3.995272 8.023723
H 2.934072 6.530589 0.235093
H -0.402344 1.048325 5.345321
H -0.335399 5.185558 4.251335
H 2.514738 4.307120 3.870176
H -0.228005 3.672763 0.644376

70

4-H_2-endo

C	-1.3762120	-4.3690890	5.1982200
C	-1.2527640	-4.6746860	6.5622960
C	-1.4760510	-5.9469420	7.0690850
C	-1.8423250	-6.9948350	6.2287540
C	-1.9714420	-6.7478530	4.8736580
C	-1.7336950	-5.4584480	4.3796630
B	-1.2165690	-2.8380470	4.6236340
H	-0.9728100	-3.8755420	7.2404280
H	-1.3699940	-6.1261300	8.1340520
H	-2.0260980	-7.9866540	6.6246920
H	-2.2649120	-7.5558080	4.2094750
P	-1.8747640	-5.2146950	2.6133170
C	-5.2722790	-1.4577920	3.3431150

C	-5.1095850	-2.1453860	4.5412270
C	-3.8454170	-2.5699240	4.9366600
C	-2.6969190	-2.3169080	4.1727780
C	-2.8934490	-1.6023400	2.9827600
C	-4.1535000	-1.1844450	2.5633900
H	-6.2567060	-1.1301880	3.0261420
H	-5.9723700	-2.3564480	5.1655590
H	-3.7465640	-3.1231970	5.8673660
H	-2.0256710	-1.3662100	2.3701870
H	-4.2622010	-0.6353940	1.6330190
C	0.8561700	-0.3025110	7.6408080
C	1.5558930	-1.2353310	6.8812310
C	0.8877030	-2.0004660	5.9342840
C	-0.4907150	-1.8765140	5.7073300
C	-1.1649180	-0.9279550	6.4831730
C	-0.5084460	-0.1510540	7.4350270
H	1.3715540	0.3016570	8.3801630
H	2.6247180	-1.3603780	7.0264050
H	1.4503390	-2.7221160	5.3459880
H	-2.2318660	-0.7854750	6.3372330
H	-1.0661260	0.5774710	8.0156890
C	0.7885900	-7.9702940	0.9563860
C	-0.4217750	-7.4400430	1.7289290
C	-0.4417510	-5.9045250	1.7124610
C	0.8672240	-5.3186340	2.2700330
C	2.0701410	-5.8654190	1.4996670
C	2.0946370	-7.3942460	1.5048110
H	0.6868290	-7.7000650	-0.1022860
H	0.8019300	-9.0625890	1.0034790
H	-0.3625850	-7.7860850	2.7673120
H	-1.3453460	-7.8454700	1.3027290
H	-0.5681570	-5.5616570	0.6769780
H	0.8440790	-4.2272020	2.2304530
H	0.9526070	-5.5856340	3.3296630
H	2.0319310	-5.5056250	0.4636550
H	2.9898070	-5.4678010	1.9362830
H	2.2402650	-7.7486270	2.5327000
H	2.9423480	-7.7623120	0.9205550
C	-4.8932900	-6.1630910	-0.0811920
C	-3.5326190	-5.7001810	0.4462360
C	-3.4447580	-5.8810630	1.9701760
C	-4.6087800	-5.1765540	2.6899250
C	-5.9554440	-5.6501470	2.1459260
C	-6.0434400	-5.4525760	0.6329190
H	-4.9858820	-7.2462890	0.0682240
H	-4.9431120	-5.9880960	-1.1593620
H	-3.3976120	-4.6378330	0.2044140
H	-2.7318200	-6.2506170	-0.0572490
H	-3.4664030	-6.9516560	2.2094260
H	-4.5456180	-5.3468170	3.7669280
H	-4.5230520	-4.0936380	2.5436480
H	-6.0972210	-6.7119910	2.3852700
H	-6.7549870	-5.1011740	2.6498370
H	-6.0001450	-4.3794890	0.4095410
H	-7.0023380	-5.8174990	0.2549920
H	-1.9111500	-3.8498920	2.3277610
H	-0.4975100	-2.8780370	3.6224670

70

4-H_2-exo

C	1.3934700	-10.9832230	13.0816590

C	1.1561760	-12.3635230	13.1341560
C	2.1153490	-13.2977180	12.7708920
C	3.3709340	-12.8920520	12.3257220
C	3.6476890	-11.5391150	12.2421790
C	2.6663100	-10.6113820	12.6156470
B	0.2986690	-9.8800440	13.5727820
H	0.1806390	-12.7058590	13.4637490
H	1.8843760	-14.3562500	12.8303830
H	4.1198390	-13.6214340	12.0405840
H	4.6190220	-11.2095680	11.8862000
P	3.0618840	-8.8730000	12.4367590
C	-3.8192420	-11.0970140	12.3908600
C	-3.0791710	-10.2403380	11.5850870
C	-1.8011120	-9.8519130	11.9724330
C	-1.2123600	-10.2974830	13.1619340
C	-1.9915480	-11.1450390	13.9610530
C	-3.2694560	-11.5455800	13.5862120
H	-4.8175120	-11.4029210	12.0963030
H	-3.5016190	-9.8696970	10.6560540
H	-1.2472150	-9.1659530	11.3357160
H	-1.5904670	-11.4869830	14.9120750
H	-3.8426600	-12.2025830	14.2329810
C	0.6036570	-8.9885830	17.9380140
C	-0.0644900	-8.1317320	17.0669570
C	-0.1443600	-8.4380880	15.7147950
C	0.4379710	-9.5945490	15.1718900
C	1.1040130	-10.4329650	16.0724510
C	1.1855320	-10.1435270	17.4336750
H	0.6658370	-8.7578480	18.9961060
H	-0.5311330	-7.2283880	17.4475120
H	-0.6812690	-7.7663080	15.0493110
H	1.5734370	-11.3421520	15.7056670
H	1.7070620	-10.8231670	18.1005250
C	0.7618630	-8.2564890	9.0224100
C	1.5163550	-9.0054220	10.1207260
C	2.1990010	-8.0071660	11.0667580
C	3.1553170	-7.0820970	10.2960870
C	2.3947140	-6.3364420	9.1961040
C	1.6800240	-7.3056560	8.2530060
H	-0.0566290	-7.6868120	9.4800130
H	0.2997000	-8.9766790	8.3424130
H	2.2749350	-9.6577300	9.6693310
H	0.8367320	-9.6489650	10.6810810
H	1.4243830	-7.4072640	11.5550720
H	3.6392660	-6.3678730	10.9682490
H	3.9520480	-7.6838800	9.8405260
H	1.6578320	-5.6698760	9.6608380
H	3.0877950	-5.7005230	8.6383130
H	2.4292370	-7.8925900	7.7066590
H	1.1095290	-6.7489040	7.5046560
C	3.3955790	-5.7436450	15.1650740
C	3.4449340	-6.4586110	13.8109320
C	3.0897570	-7.9385940	13.9989540
C	4.0216150	-8.6304420	15.0041940
C	3.9679020	-7.8986620	16.3460650
C	4.3093820	-6.4166300	16.1899590
H	2.3635220	-5.7642310	15.5344030
H	3.6705810	-4.6931220	15.0351310
H	4.4516630	-6.3626650	13.3817140
H	2.7468860	-5.9798700	13.1175830
H	2.0687490	-8.0101800	14.3807620

H	3.7174360	-9.6713770	15.1380590
H	5.0513410	-8.6281620	14.6200320
H	2.9597670	-8.0070370	16.7603110
H	4.6538330	-8.3774980	17.0502610
H	5.3536230	-6.3129120	15.8664520
H	4.2261560	-5.9077300	17.1541050
H	4.4097730	-8.8882110	12.0212160
H	0.5630450	-8.8242750	12.9869250

139

4-H_2O-4-TS

P 1.705263 -0.823216 0.326077
B 0.825876 2.013577 0.340289
C -0.045070 -0.429052 0.098237
C -1.048606 -1.388442 -0.007854
H -0.800079 -2.435943 0.031795
C -2.362576 -0.987589 -0.176110
H -3.141751 -1.734761 -0.267446
C -2.668809 0.368778 -0.227845
H -3.697499 0.685578 -0.360238
C -1.666897 1.317473 -0.086402
H -1.920070 2.372832 -0.084296
C -0.332394 0.944025 0.084868
C 1.487726 2.694164 -0.950680
C 2.723009 3.353508 -0.865737
C 3.352801 3.881591 -1.985613
H 4.313319 4.375445 -1.886600
C 2.745079 3.781880 -3.232335
C 1.509153 3.157953 -3.341977
H 1.024608 3.080901 -4.309200
C 0.896284 2.619868 -2.215067
C 0.614561 2.919622 1.641923
C 0.881399 4.292250 1.664915
C 0.668072 5.058004 2.807709
H 0.881324 6.121431 2.792627
C 0.179728 4.463958 3.962767
C -0.112209 3.103480 3.962543
H -0.512251 2.633222 4.854448
C 0.094950 2.352613 2.814840
C 2.210601 -1.659046 1.868139
H 2.139924 -2.724082 1.654228
C 1.231018 -1.344588 3.007342
H 1.261484 -0.272458 3.229359
H 0.210600 -1.597768 2.709014
C 1.617625 -2.146158 4.251679
H 0.935030 -1.895163 5.068346
H 1.484355 -3.211904 4.040103
C 3.064303 -1.878553 4.666033
H 3.167350 -0.832094 4.981163
H 3.328941 -2.496559 5.528326
C 4.028642 -2.152758 3.512047
H 3.996492 -3.219750 3.260221
H 5.056672 -1.922326 3.805162
C 3.658927 -1.338362 2.268183
H 4.347175 -1.579850 1.454148
H 3.760887 -0.270190 2.496381
C 2.816815 -0.956557 -1.131645
H 3.197177 -1.976778 -1.067379
C 2.025029 -0.815329 -2.439477
H 1.228033 -1.558994 -2.465218
H 1.563768 0.175437 -2.484877

C 2.940826 -1.009835 -3.649711
H 3.280018 -2.050310 -3.672288
H 2.362913 -0.839022 -4.562236
C 4.154909 -0.083881 -3.603247
H 3.824546 0.960511 -3.675610
H 4.807296 -0.276252 -4.459592
C 4.928754 -0.274187 -2.299929
H 5.787549 0.401999 -2.259862
H 5.313359 -1.298956 -2.240465
C 4.023085 -0.003779 -1.095044
H 3.690092 1.036969 -1.162085
H 4.599060 -0.130969 -0.172073
O 2.013388 0.803652 0.826364
H 2.923073 1.094624 0.704577
H 0.014697 5.057612 4.855246
H 3.229749 4.193622 -4.110475
H -0.170747 1.300175 2.822024
H 1.251216 4.781187 0.770069
H 3.208228 3.460384 0.102467
H -0.060548 2.118743 -2.325497
B 1.428556 -4.121610 -0.394789
H 1.235760 -2.806374 -0.293246
C 2.960638 -4.228205 -0.927678
C 3.231652 -4.463915 -2.283206
C 4.082643 -3.984872 -0.118926
C 4.522735 -4.456668 -2.798625
H 2.404285 -4.660351 -2.957240
C 5.380213 -3.966642 -0.619279
H 3.948440 -3.797543 0.940474
C 5.608632 -4.202379 -1.969452
H 4.680768 -4.651277 -3.854757
H 6.214375 -3.776663 0.049085
H 6.617641 -4.196730 -2.367178
C 1.108850 -4.700735 1.099720
C 2.023689 -5.414595 1.885991
C -0.171278 -4.541220 1.653581
C 1.699742 -5.901885 3.148996
H 3.003458 -5.642012 1.483442
C -0.512330 -5.022242 2.911398
H -0.948459 -4.063352 1.066365
C 0.430443 -5.702062 3.674693
H 2.438861 -6.459659 3.715237
H -1.518992 -4.879516 3.290534
H 0.173106 -6.085215 4.656074
C 0.346617 -4.658418 -1.490261
C -0.449976 -3.767570 -2.219891
C 0.188548 -6.035935 -1.785117
C -1.379271 -4.172915 -3.169710
H -0.340280 -2.704321 -2.048864
C -0.749495 -6.436016 -2.741847
C -1.538604 -5.523512 -3.428923
H -1.966703 -3.433813 -3.705026
H -0.864295 -7.487048 -2.979047
H -2.256745 -5.869058 -4.164728
P 1.299057 -7.284657 -0.963084
C 0.117218 -8.044646 0.279412
C 1.482279 -8.559871 -2.320099
H -0.278281 -7.153689 0.784474
C 0.867629 -8.855183 1.348853
C -1.074165 -8.838867 -0.263035
H 0.503793 -8.899886 -2.680690

C 2.241608 -7.920614 -3.495023
C 2.235138 -9.798343 -1.811781
H 1.302734 -9.757036 0.903399
H 1.698705 -8.265597 1.742080
C -0.071590 -9.271680 2.482176
C -2.011066 -9.272056 0.869522
H -1.631736 -8.240075 -0.987309
H -0.713205 -9.731684 -0.791193
H 1.677724 -7.070245 -3.888162
H 3.192533 -7.518214 -3.121716
C 2.519042 -8.923858 -4.615556
C 2.519186 -10.796902 -2.937270
H 3.181347 -9.483655 -1.352748
H 1.656380 -10.296746 -1.030499
H 0.473853 -9.863602 3.224656
H -0.428937 -8.369175 2.993225
C -1.267680 -10.061197 1.947998
H -2.454095 -8.375669 1.322264
H -2.838608 -9.864903 0.466255
H 1.566426 -9.249398 -5.053822
H 3.084457 -8.438594 -5.417520
C 3.275339 -10.145388 -4.094134
H 3.083255 -11.648231 -2.543237
H 1.566901 -11.195767 -3.310875
H -0.911183 -11.007169 1.518924
H -1.948699 -10.322847 2.764187
H 4.267052 -9.830968 -3.744119
H 3.437823 -10.869008 -4.899139

71

4-H_2O-endo'

P	0.7578388	-0.3958485	-0.4577600
B	-1.9469132	-0.3360765	1.2491340
C	-0.8723522	-0.4547295	-1.2171140
C	-0.9361412	-0.4921855	-2.6152110
H	-0.0317632	-0.5095575	-3.2160780
C	-2.1664372	-0.5147485	-3.2496020
H	-2.2231972	-0.5420995	-4.3315320
C	-3.3207012	-0.5047275	-2.4748570
H	-4.2927312	-0.5229465	-2.9566990
C	-3.2405162	-0.4767765	-1.0890150
H	-4.1568702	-0.4761925	-0.5088960
C	-2.0197972	-0.4519665	-0.4008440
C	-1.3534212	1.1590245	1.5919860
C	-0.4036692	1.3505595	2.6005300
C	0.1166878	2.6067915	2.9007610
H	0.8524218	2.7156525	3.6920130
C	-0.3039642	3.7233345	2.1883710
C	-1.2538142	3.5677005	1.1827910
H	-1.5967872	4.4323035	0.6230100
C	-1.7642142	2.3063175	0.8966220
C	-3.4143052	-0.5733115	1.9190690
C	-4.2003362	0.4390355	2.4779130
C	-5.4522862	0.1791535	3.0288350
H	-6.0336612	0.9902825	3.4560420
C	-5.9567042	-1.1147945	3.0394520
C	-5.1964182	-2.1443265	2.4950270
H	-5.5787122	-3.1604465	2.4964340
C	-3.9495662	-1.8704095	1.9463420
C	1.7965488	-1.6784835	-1.2506120
H	1.8137088	-1.4498935	-2.3234840

C	1.1086248	-3.0425635	-1.0443680
H	0.9838628	-3.2212285	0.0303850
H	0.1036248	-3.0227615	-1.4713890
C	1.9294568	-4.1735155	-1.6638680
H	1.4341918	-5.1283345	-1.4690150
H	1.9548118	-4.0477905	-2.7537800
C	3.3583508	-4.1902175	-1.1228520
H	3.3353688	-4.4095015	-0.0481220
H	3.9361938	-4.9886735	-1.5962700
C	4.0404758	-2.8419125	-1.3484810
H	4.1460978	-2.6663535	-2.4266150
H	5.0508138	-2.8466875	-0.9303630
C	3.2423748	-1.6895645	-0.7325420
H	3.7363948	-0.7433425	-0.9643230
H	3.2383118	-1.7918945	0.3599850
C	1.4638348	1.2879465	-0.5418370
H	0.6057138	1.9069765	-0.2499280
C	2.5746018	1.5305315	0.4927960
H	2.2106268	1.2740385	1.4903150
H	3.4427738	0.8979375	0.2819390
C	3.0062058	2.9977665	0.4557530
H	2.1627088	3.6179635	0.7784450
H	3.8150278	3.1594145	1.1739460
C	3.4473688	3.4146665	-0.9480390
H	4.3466308	2.8507555	-1.2292560
H	3.7243298	4.4724685	-0.9581730
C	2.3464908	3.1518295	-1.9765550
H	2.6876128	3.4193735	-2.9805840
H	1.4822258	3.7882905	-1.7524240
C	1.8955738	1.6887995	-1.9582300
H	2.7237638	1.0486955	-2.2902610
H	1.0707508	1.5474805	-2.6624320
H	0.6083538	-0.7646165	0.8913440
O	-1.0016072	-1.4082155	1.7347000
H	-1.3002982	-1.7251185	2.5852280
H	-6.9313592	-1.3206275	3.4692790
H	0.0971268	4.7048885	2.4181610
H	-3.3693662	-2.6837305	1.5174920
H	-3.8240222	1.4575405	2.4899610
H	-2.4982022	2.2055615	0.1009080
H	-0.0577492	0.4830895	3.1564400

71

4-H_2O-endo

C	1.7070850	-7.4157590	4.1868610
C	2.7257320	-7.4431060	5.1504840
C	2.4750880	-7.3401400	6.5116310
C	1.1743460	-7.1977180	6.9840050
C	0.1341560	-7.1646730	6.0721960
C	0.4049180	-7.2786720	4.7024550
B	2.0431220	-7.4241330	2.5665480
H	3.7502790	-7.5485580	4.8103660
H	3.3021560	-7.3660740	7.2135290
H	0.9750800	-7.1086750	8.0455260
H	-0.8833770	-7.0365100	6.4301320
P	-1.0026820	-7.2243140	3.5944640
C	1.2466870	-3.2653420	1.1025590
C	0.7770390	-4.3667250	0.3962230
C	1.0456590	-5.6544270	0.8539070
C	1.7746340	-5.8940540	2.0241680
C	2.2411700	-4.7631500	2.7085050

C	1.9897980	-3.4711430	2.2611870
H	1.0437190	-2.2592040	0.7513320
H	0.2068270	-4.2222860	-0.5165010
H	0.6727620	-6.5079520	0.2935930
H	2.8094230	-4.8984550	3.6255010
H	2.3683990	-2.6205070	2.8193790
C	6.1809220	-8.9170930	1.8360230
C	5.1840490	-9.7580390	2.3199560
C	3.9055950	-9.2613870	2.5407770
C	3.5705690	-7.9214810	2.2904820
C	4.5925010	-7.1029440	1.8006440
C	5.8790110	-7.5867020	1.5768400
H	7.1820770	-9.2970210	1.6615650
H	5.4067440	-10.8001330	2.5275430
H	3.1385220	-9.9286200	2.9266580
H	4.3784330	-6.0611490	1.5822840
H	6.6471160	-6.9220520	1.1938740
C	-3.7966120	-10.0359640	4.9442220
C	-2.9440320	-8.7637890	4.9436820
C	-2.0273090	-8.7373390	3.7120500
C	-1.1358920	-9.9900020	3.6569360
C	-1.9956640	-11.2540070	3.6592630
C	-2.9299760	-11.2940530	4.8695380
H	-4.4762790	-10.0105290	4.0829820
H	-4.4237620	-10.0558420	5.8398860
H	-2.3289180	-8.7458420	5.8509600
H	-3.5921830	-7.8824250	4.9719950
H	-2.6525450	-8.7108980	2.8094110
H	-0.4762080	-9.9438160	2.7885990
H	-0.4810470	-9.9895480	4.5363600
H	-2.5902330	-11.2919610	2.7375710
H	-1.3475470	-12.1343130	3.6504710
H	-2.3287130	-11.3700770	5.7840820
H	-3.5640870	-12.1842730	4.8337560
C	-3.9656540	-4.3699230	3.0507610
C	-3.1643100	-5.6628400	2.8801670
C	-2.0101130	-5.7335880	3.8940680
C	-1.1113420	-4.4878290	3.8094990
C	-1.9298300	-3.2088930	3.9771970
C	-3.0648230	-3.1380920	2.9560380
H	-4.4642900	-4.3823490	4.0283120
H	-4.7545260	-4.3285340	2.2946080
H	-2.7463540	-5.6923510	1.8655660
H	-3.8258030	-6.5292060	2.9815540
H	-2.4221260	-5.8132090	4.9079000
H	-0.3208640	-4.5408920	4.5612090
H	-0.6110580	-4.4675530	2.8356300
H	-2.3475160	-3.1672940	4.9916260
H	-1.2666130	-2.3466610	3.8701940
H	-2.6344650	-3.0815590	1.9485800
H	-3.6546800	-2.2289790	3.1021460
H	-0.5517690	-7.1489970	2.2768760
O	1.0835510	-8.3888210	1.9109020
H	1.5369320	-8.8350520	1.1979070

71

4-H_2O-exo'

C	-1.2139520	-4.2602620	5.3670450
C	-1.0359200	-4.8326110	6.6296630
C	-1.2497270	-6.1832780	6.8576220
C	-1.6483330	-7.0165200	5.8158650

C	-1.8289010	-6.4820590	4.5519450
C	-1.6114950	-5.1148250	4.3318770
B	-1.0066730	-2.6881840	5.0604850
H	-0.7106020	-4.1969170	7.4471300
H	-1.1009940	-6.5951740	7.8502490
H	-1.8135670	-8.0738140	5.9873630
H	-2.1375750	-7.1307650	3.7386520
P	-1.8788610	-4.5284480	2.6365500
C	-4.5515800	-0.1677850	6.0372850
C	-4.5573050	-1.5141710	6.3771390
C	-3.4399250	-2.3052030	6.1196160
C	-2.2872740	-1.7874630	5.5219600
C	-2.2985050	-0.4161200	5.2259520
C	-3.4095040	0.3832400	5.4637480
H	-5.4211000	0.4511420	6.2306590
H	-5.4351420	-1.9520650	6.8418180
H	-3.4718050	-3.3576930	6.3878880
H	-1.4047030	0.0398570	4.8042040
H	-3.3835790	1.4391090	5.2141010
C	2.8613430	-1.2434910	6.7120780
C	2.7846960	-1.7052300	5.4037090
C	1.5681270	-2.1405180	4.8889450
C	0.3949570	-2.1274530	5.6507870
C	0.4985700	-1.6428610	6.9603400
C	1.7098120	-1.2127150	7.4904210
H	3.8076370	-0.9038890	7.1194720
H	3.6756060	-1.7259140	4.7836040
H	1.5215120	-2.4927870	3.8622500
H	-0.3963470	-1.5865920	7.5750830
H	1.7548400	-0.8433020	8.5100370
C	-4.7118260	-1.5542780	1.5616380
C	-3.3633480	-2.0904140	2.0484730
C	-3.4161040	-3.6131590	2.1843950
C	-4.5450840	-4.0496630	3.1332300
C	-5.8907950	-3.4785750	2.6815900
C	-5.8299280	-1.9589540	2.5226120
H	-4.9288840	-1.9408630	0.5570910
H	-4.6582050	-0.4653140	1.4791340
H	-3.1572970	-1.6430650	3.0226120
H	-2.5665120	-1.7937590	1.3571930
H	-3.6302010	-4.0330200	1.1907650
H	-4.5977790	-5.1418260	3.1894610
H	-4.3149450	-3.6856880	4.1384990
H	-6.1768660	-3.9342100	1.7245000
H	-6.6618270	-3.7550200	3.4061990
H	-5.6397640	-1.5007200	3.5008200
H	-6.7920920	-1.5778910	2.1685190
C	0.3889120	-4.7449010	-0.8593010
C	-0.8037790	-4.9163410	0.0863840
C	-0.4730740	-4.3388260	1.4683730
C	0.7831800	-4.9924240	2.0617230
C	1.9695480	-4.8292470	1.1109030
C	1.6548570	-5.3800110	-0.2811090
H	0.5635150	-3.6749300	-1.0274860
H	0.1527170	-5.1825530	-1.8333530
H	-1.0394240	-5.9835460	0.1789870
H	-1.6890010	-4.4286560	-0.3357090
H	-0.2901190	-3.2669330	1.3749290
H	1.0042110	-4.5529200	3.0364570
H	0.5925750	-6.0607710	2.2290130
H	2.2203210	-3.7638280	1.0339900

H	2.8472810	-5.3288430	1.5295700
H	1.5125180	-6.4661350	-0.2140700
H	2.5001080	-5.2144650	-0.9549880
H	-2.3496820	-5.7760090	2.1338110
O	-0.9541890	-2.7097280	3.5332050
H	-1.1078480	-1.8418540	3.1609950

71

4-H_2O-exo

P	1.5341898	-0.1144146	-0.0505569
H	2.8577188	-0.5993656	-0.2211249
O	-0.6346482	0.7860674	0.1213461
H	-1.0651012	0.8569344	0.9738941
B	-1.5262012	0.0393954	-0.8664399
C	0.9063128	-0.4782406	-1.7042169
C	-0.4576812	-0.3755346	-2.0142939
C	-0.8247132	-0.6809266	-3.3279849
H	-1.8705602	-0.5956646	-3.6056569
C	0.1056608	-1.0712296	-4.2801669
H	-0.2177572	-1.2973616	-5.2908079
C	1.4537828	-1.1687596	-3.9485869
H	2.1845708	-1.4714396	-4.6894259
C	1.8550718	-0.8727036	-2.6570109
H	2.9052428	-0.9491126	-2.3933799
C	-2.6854552	1.0207934	-1.4312289
C	-3.8842722	0.5287474	-1.9603599
C	-4.8511032	1.3729314	-2.4965629
H	-5.7700982	0.9586444	-2.8991039
C	-4.6466812	2.7476364	-2.5072059
C	-3.4701812	3.2644694	-1.9768589
H	-3.3028512	4.3371834	-1.9745329
C	-2.5087372	2.4086584	-1.4511839
C	-2.1352262	-1.2827476	-0.1150049
C	-1.8495922	-2.6017066	-0.4837189
C	-2.2857862	-3.6892916	0.2692871
H	-2.0441292	-4.6988756	-0.0480709
C	-3.0222352	-3.4852376	1.4290971
C	-3.3406632	-2.1855156	1.8110961
H	-3.9348382	-2.0125356	2.7027781
C	-2.9113132	-1.1115976	1.0410191
C	0.9587158	-1.2333576	1.2829241
H	-0.0914202	-1.4077156	1.0495621
C	1.0363618	-0.6264996	2.6872091
H	0.5140428	0.3340224	2.7224851
H	2.0818178	-0.4348266	2.9614931
C	0.4134528	-1.5971176	3.6948351
H	0.4787038	-1.1777296	4.7029001
H	-0.6515822	-1.7113246	3.4584211
C	1.0929358	-2.9667716	3.6412421
H	0.6069618	-3.6527146	4.3405781
H	2.1354718	-2.8654026	3.9706541
C	1.0628548	-3.5501156	2.2273941
H	0.0245018	-3.7476826	1.9371081
H	1.5939728	-4.5058056	2.1983351
C	1.6794158	-2.5846176	1.2120991
H	2.7481308	-2.4525576	1.4258221
H	1.5978678	-3.0000176	0.2024181
C	1.9359368	1.6297604	0.3741081
H	1.1961228	1.9380234	1.1150881
C	3.3423868	1.7528654	0.9750191
H	3.4442418	1.1172744	1.8604581

H	4.0839448	1.4034174	0.2458291
C	3.6400488	3.2107364	1.3357461
H	2.9551028	3.5307384	2.1308151
H	4.6527818	3.2910534	1.7408021
C	3.4745458	4.1287454	0.1233771
H	3.6544628	5.1683604	0.4111901
H	4.2341218	3.8726934	-0.6262089
C	2.0855238	3.9827314	-0.5004049
H	2.0015248	4.6070874	-1.3939839
H	1.3281398	4.3422964	0.2073231
C	1.7773778	2.5287524	-0.8605299
H	2.4641108	2.1935954	-1.6486299
H	0.7646128	2.4320904	-1.2544449
H	-4.0702652	-0.5421996	-1.9406529
H	-5.3998722	3.4105944	-2.9196969
H	-1.5955972	2.8241254	-1.0340219
H	-3.1993312	-0.1055486	1.3410781
H	-3.3574052	-4.3289046	2.0226901
H	-1.2574472	-2.7872526	-1.3754659

71

4-H_2O-LA

P	0.8086598	-0.6214212	-0.2130191
B	-2.0628262	-0.1337932	1.2341319
C	-0.8057852	-0.5865352	-1.1090721
C	-0.8543742	-0.7205802	-2.4988441
H	0.0591478	-0.8599042	-3.0663881
C	-2.0609152	-0.6856732	-3.1838381
H	-2.0762842	-0.7893892	-4.2631691
C	-3.2387232	-0.5199542	-2.4717011
H	-4.1914252	-0.4920452	-2.9896721
C	-3.1976322	-0.3981032	-1.0884851
H	-4.1294702	-0.2790362	-0.5461311
C	-1.9990002	-0.4284352	-0.3655571
C	-1.4355542	1.3165278	1.5865189
C	-0.5693112	1.5669678	2.6540509
C	-0.0731372	2.8392218	2.9222839
H	0.6064538	2.9908058	3.7543319
C	-0.4455552	3.9121968	2.1245989
C	-1.3167462	3.6972298	1.0617189
H	-1.6173732	4.5268458	0.4305129
C	-1.7963402	2.4203908	0.8008369
C	-3.4816422	-0.4256842	1.9393639
C	-4.2063672	0.5595408	2.6154729
C	-5.4331282	0.2825468	3.2126519
H	-5.9739702	1.0699688	3.7270829
C	-5.9622942	-0.9993722	3.1546059
C	-5.2587812	-2.0009432	2.4926799
H	-5.6651892	-3.0056672	2.4401869
C	-4.0404462	-1.7116922	1.8935419
C	1.8704438	-1.7517332	-1.2321301
H	1.9037388	-1.3796992	-2.2633451
C	1.2586028	-3.1618792	-1.2402341
H	1.1710178	-3.5159392	-0.2043391
H	0.2452418	-3.1330302	-1.6481641
C	2.1192668	-4.1447622	-2.0356651
H	1.6712378	-5.1420292	-1.9987751
H	2.1291118	-3.8426102	-3.0907581
C	3.5533388	-4.1835932	-1.5081711
H	3.5496968	-4.5826212	-0.4856911
H	4.1637238	-4.8632902	-2.1099481

C	4.1696458	-2.7851112	-1.4988091
H	4.2668148	-2.4272772	-2.5318801
H	5.1801708	-2.8152282	-1.0806291
C	3.3133208	-1.7932012	-0.7069521
H	3.7671818	-0.8009212	-0.7613161
H	3.3055808	-2.0803352	0.3526589
C	1.4551418	1.1075238	-0.4403451
H	0.5702008	1.7133048	-0.2121041
C	2.5210988	1.4715208	0.6045909
H	2.1414968	1.2525138	1.6062199
H	3.4202348	0.8611188	0.4612879
C	2.8994568	2.9504328	0.5004839
H	2.0243708	3.5546438	0.7666749
H	3.6830888	3.1864018	1.2272159
C	3.3570508	3.3129228	-0.9130311
H	4.2851348	2.7729688	-1.1434821
H	3.5909328	4.3800258	-0.9739411
C	2.2931628	2.9448468	-1.9481261
H	2.6448978	3.1788708	-2.9576241
H	1.3958118	3.5516448	-1.7744601
C	1.9132498	1.4640968	-1.8579041
H	2.7838378	0.8547548	-2.1335301
H	1.1211558	1.2401508	-2.5777701
H	-0.1688572	-1.1844422	1.5161199
O	-1.0888922	-1.2947652	1.9163789
H	-1.0632472	-1.2194372	2.8745519
H	-6.9168572	-1.2189932	3.6206759
H	-0.0613572	4.9056498	2.3281469
H	-3.5074352	-2.4995262	1.3688719
H	-3.8053092	1.5663548	2.6769689
H	-2.4602982	2.2699098	-0.0452541
H	-0.2481972	0.7567568	3.3048389

71

4-H_2O-TS

P	3.1939650	2.0597660	2.8420540
B	0.4898730	2.3694510	4.4314550
C	1.5892640	2.0889860	1.9903350
C	1.5094880	2.0211020	0.5974120
H	2.4102930	1.9453690	-0.0038410
C	0.2751190	2.0398990	-0.0334540
H	0.2134470	1.9885340	-1.1145860
C	-0.8764450	2.1190530	0.7395660
H	-1.8496050	2.1294120	0.2599690
C	-0.7897300	2.1773250	2.1248580
H	-1.7026410	2.2291850	2.7087620
C	0.4364780	2.1659970	2.8003450
C	1.0298280	3.8801780	4.7462080
C	1.9209720	4.1612350	5.7868810
C	2.3846010	5.4486080	6.0418470
H	3.0807800	5.6228910	6.8561410
C	1.9589330	6.5094530	5.2532420
C	1.0624110	6.2659840	4.2176850
H	0.7166790	7.0857240	3.5962060
C	0.6115470	4.9740630	3.9745940
C	-0.9185680	2.0370910	5.1574900
C	-1.7501330	3.0152130	5.7101830
C	-2.9677350	2.6917400	6.3029060
H	-3.5901830	3.4756900	6.7223020
C	-3.3840920	1.3685510	6.3638430
C	-2.5735930	0.3735290	5.8266930

H	-2.8890240	-0.6642200	5.8685670
C	-1.3636050	0.7093860	5.2333200
C	4.2452370	0.8411760	1.9474130
H	4.2970740	1.1626110	0.8993520
C	3.5683830	-0.5396420	2.0048780
H	3.4384960	-0.8294790	3.0553410
H	2.5678480	-0.4867500	1.5693580
C	4.4052800	-1.5991030	1.2870400
H	3.9144210	-2.5724990	1.3720020
H	4.4489910	-1.3602910	0.2168040
C	5.8249980	-1.6646740	1.8492320
H	5.7840780	-1.9988190	2.8936840
H	6.4175020	-2.4047640	1.3040500
C	6.5009260	-0.2957340	1.7848010
H	6.6300460	-0.0061820	0.7341160
H	7.5017810	-0.3409250	2.2234880
C	5.6772680	0.7792530	2.4990060
H	6.1709250	1.7472180	2.3865940
H	5.6443810	0.5604220	3.5741030
C	3.9109820	3.7523020	2.7421920
H	3.0437770	4.3787980	2.9848010
C	4.9738610	4.0177190	3.8194170
H	4.5664740	3.7768910	4.8045640
H	5.8486080	3.3772990	3.6617240
C	5.4126580	5.4829230	3.7803130
H	4.5581870	6.1106090	4.0577700
H	6.1920970	5.6551420	4.5284710
C	5.9108380	5.8806020	2.3899730
H	6.8194560	5.3112580	2.1529830
H	6.1904650	6.9379380	2.3769070
C	4.8516440	5.6034350	1.3220810
H	5.2322470	5.8606710	0.3292610
H	3.9786170	6.2415670	1.5049220
C	4.4039200	4.1394190	1.3431260
H	5.2480910	3.4996100	1.0542490
H	3.6106500	3.9828170	0.6064630
H	2.6120480	1.6036940	4.2663170
O	1.5290290	1.3254240	4.9564560
H	1.5580780	1.3172690	5.9152930
H	-4.3312280	1.1126920	6.8269010
H	2.3175930	7.5149110	5.4453760
H	-0.7420400	-0.0758000	4.8121330
H	-1.4394790	4.0552650	5.6809820
H	-0.0757530	4.8051430	3.1504610
H	2.2812350	3.3553660	6.4217120

71

4-H_2O-upsd-TS1

P	1.1234860	-0.1711610	2.9639020
B	1.1826170	3.3072270	2.7257420
C	0.0438530	0.8990540	1.9206260
C	-0.8252500	0.2677310	1.0258410
H	-0.9098080	-0.8114070	1.0101460
C	-1.6079210	0.9896620	0.1352980
H	-2.2807010	0.4715080	-0.5383430
C	-1.5170010	2.3700250	0.1299820
H	-2.1241320	2.9597390	-0.5481870
C	-0.6468750	3.0018640	1.0099520
H	-0.5959860	4.0860440	0.9973870
C	0.1568380	2.3128750	1.9291610
C	2.6304410	3.4260940	2.0477670

C	3.7792340	3.6739980	2.8127520
C	5.0425730	3.7538220	2.2404680
H	5.9095390	3.9416980	2.8653330
C	5.1967930	3.5923030	0.8674700
C	4.0759970	3.3519960	0.0825110
H	4.1818550	3.2279410	-0.9904440
C	2.8173020	3.2646090	0.6679520
C	0.5390730	4.6080980	3.3819630
C	1.2194170	5.8327920	3.4346090
C	0.6535360	6.9551420	4.0287370
H	1.2016750	7.8914830	4.0451680
C	-0.6125820	6.8821800	4.5954530
C	-1.3098860	5.6779770	4.5586270
H	-2.3002930	5.6113630	4.9968810
C	-0.7402410	4.5639720	3.9610600
C	0.4485290	-1.9147060	2.8690580
H	0.3256720	-2.1827900	1.8111270
C	-0.9101740	-1.9804770	3.5830350
H	-0.7733610	-1.6532140	4.6217380
H	-1.6213840	-1.2845110	3.1318180
C	-1.4798100	-3.3999420	3.5734360
H	-2.4332540	-3.4180470	4.1090070
H	-1.6931700	-3.6978520	2.5388300
C	-0.4994990	-4.3943390	4.1950700
H	-0.3715720	-4.1551470	5.2583800
H	-0.9029160	-5.4097790	4.1462470
C	0.8595430	-4.3311750	3.4989640
H	0.7502950	-4.6730090	2.4618570
H	1.5678040	-5.0115370	3.9807760
C	1.4328510	-2.9112030	3.4968070
H	2.3811180	-2.9056430	2.9552090
H	1.6526950	-2.6034330	4.5274760
C	2.7220720	-0.1253140	2.0414010
H	2.7949020	0.9381920	1.7920850
C	3.9638490	-0.4566530	2.8799550
H	3.9745260	0.1530260	3.7890890
H	3.9510620	-1.5051150	3.1974940
C	5.2332680	-0.1824840	2.0694800
H	5.2944220	0.8929290	1.8663110
H	6.1146420	-0.4491790	2.6604160
C	5.2279150	-0.9485830	0.7457380
H	5.2569990	-2.0274530	0.9494770
H	6.1285390	-0.7122540	0.1717200
C	3.9801940	-0.6240140	-0.0766530
H	3.9722250	-1.2035930	-1.0046580
H	4.0000530	0.4355090	-0.3568910
C	2.6999910	-0.8990030	0.7176390
H	2.6187510	-1.9767280	0.9081480
H	1.8253280	-0.6075130	0.1281340
O	1.3984540	1.3244200	4.1172050
H	-1.0557750	7.7566010	5.0596440
H	6.1807690	3.6532630	0.4155020
H	-1.2958880	3.6310310	3.9374110
H	2.2057700	5.9125480	2.9893200
H	3.6758230	3.8039270	3.8868890
H	1.9585080	3.0578370	0.0362960
H	2.0467360	1.1066570	4.8144230
H	1.4604820	2.6601530	3.9852680

71

4-H_2O-upsd-TS2

P	-0.0172670	-1.1994659	-0.0885454
B	-0.3734570	2.1776611	0.7275476
C	-1.0699750	0.0833131	-0.8679084
C	-1.7589880	-0.3405249	-2.0080974
H	-1.6528370	-1.3644579	-2.3541584
C	-2.5894050	0.5140301	-2.7156414
H	-3.1064970	0.1633391	-3.6014044
C	-2.7498580	1.8158011	-2.2679444
H	-3.3964310	2.5038431	-2.8011204
C	-2.0843450	2.2374911	-1.1253674
H	-2.2301540	3.2581481	-0.7902154
C	-1.2210060	1.4036711	-0.3945524
C	1.1816090	2.3165051	0.4460746
C	2.2039370	1.9328751	1.3171626
C	3.5403210	2.1128541	0.9829146
H	4.3140540	1.7885221	1.6703686
C	3.8882220	2.6983821	-0.2291494
C	2.8874240	3.1051901	-1.1023274
H	3.1446210	3.5653391	-2.0504104
C	1.5531330	2.9094941	-0.7667304
C	-1.0965340	3.3397221	1.5398476
C	-0.4055720	4.5044791	1.8887436
C	-1.0275000	5.5325121	2.5897606
H	-0.4712610	6.4286161	2.8424216
C	-2.3566020	5.4089801	2.9707456
C	-3.0632210	4.2549921	2.6447876
H	-4.1005480	4.1506371	2.9438566
C	-2.4388910	3.2405751	1.9345866
C	-1.1255930	-2.6560099	0.1720326
H	-1.2384240	-3.1707659	-0.7923094
C	-2.5149860	-2.2258829	0.6775886
H	-2.3864620	-1.6421879	1.5983266
H	-3.0042740	-1.5692599	-0.0439014
C	-3.4086800	-3.4347729	0.9616776
H	-4.3760340	-3.0931019	1.3415006
H	-3.6086900	-3.9601339	0.0185846
C	-2.7587630	-4.4031039	1.9484516
H	-2.6574210	-3.9099519	2.9232596
H	-3.3978150	-5.2779499	2.1004516
C	-1.3762240	-4.8295709	1.4584386
H	-1.4829200	-5.4182609	0.5378246
H	-0.8954710	-5.4810189	2.1941106
C	-0.4808520	-3.6190989	1.1872586
H	0.4997120	-3.9536939	0.8454946
H	-0.3069650	-3.0673359	2.1166516
C	1.2020930	-1.6043189	-1.4053814
H	0.6284350	-1.8838119	-2.2994754
C	2.0151160	-0.3355959	-1.7132744
H	1.3500120	0.4581641	-2.0595084
H	2.4731420	0.0259661	-0.7861944
C	3.1089020	-0.5884049	-2.7480634
H	2.6525490	-0.8390879	-3.7148834
H	3.6803420	0.3328441	-2.8939554
C	4.0279070	-1.7281819	-2.3134014
H	4.5498760	-1.4371579	-1.3932884
H	4.7955440	-1.9137929	-3.0705564
C	3.2210590	-2.9996929	-2.0557354
H	3.8759350	-3.8092659	-1.7199994
H	2.7657900	-3.3324439	-2.9978094
C	2.1182030	-2.7746599	-1.0173964
H	2.5694400	-2.5624949	-0.0412104

H	1.5349200	-3.6933489	-0.9125054
O	0.6319920	-0.7861079	1.2032696
H	-2.8424820	6.2069211	3.5217146
H	4.9317460	2.8365521	-0.4901754
H	-3.0017490	2.3466881	1.6804836
H	0.6360150	4.6082991	1.6032326
H	1.9549620	1.4491181	2.2541786
H	0.7845440	3.2125451	-1.4727334
H	-0.1536230	0.6838771	1.9701816
H	-0.4937830	1.2969671	2.2918026

71

4-H_2O-upsd

P	1.4451970	-0.0955210	2.7361590
B	1.1631450	3.1318410	3.4916660
C	0.2868750	1.0465980	1.9665530
C	-0.4863370	0.5303740	0.9163390
H	-0.3975040	-0.5084270	0.6164980
C	-1.3859450	1.3383140	0.2448820
H	-1.9794200	0.9368290	-0.5680330
C	-1.5166500	2.6637160	0.6399930
H	-2.2217820	3.3129170	0.1317400
C	-0.7499370	3.1649210	1.6813860
H	-0.8729430	4.2030070	1.9681240
C	0.1906910	2.3944020	2.3827300
C	2.6771200	3.2011670	2.9066720
C	3.7989010	2.9911830	3.7169670
C	5.0961800	3.0826910	3.2220090
H	5.9413730	2.9113850	3.8811770
C	5.3118360	3.3955470	1.8848950
C	4.2179780	3.6167860	1.0546040
H	4.3719860	3.8702790	0.0104430
C	2.9273860	3.5184840	1.5631290
C	0.5872510	4.5825710	3.9315630
C	1.1452220	5.7934180	3.5109940
C	0.6036280	7.0214040	3.8833330
H	1.0651270	7.9416240	3.5388980
C	-0.5201380	7.0720090	4.6967260
C	-1.0934200	5.8827630	5.1362920
H	-1.9683180	5.9070530	5.7784940
C	-0.5423840	4.6657390	4.7577720
C	0.7013740	-1.7610740	2.7862500
H	0.5104760	-2.0431390	1.7436030
C	-0.6372380	-1.7064330	3.5472440
H	-0.4459960	-1.3454730	4.5636170
H	-1.3136930	-0.9895040	3.0768150
C	-1.2907760	-3.0872440	3.6110350
H	-2.2214560	-3.0204650	4.1806480
H	-1.5635610	-3.4077800	2.5974090
C	-0.3535680	-4.1198740	4.2349620
H	-0.1599140	-3.8468980	5.2795800
H	-0.8265710	-5.1056220	4.2474540
C	0.9703240	-4.1798170	3.4755250
H	0.7866150	-4.5469730	2.4575970
H	1.6521900	-4.8921130	3.9479660
C	1.6478470	-2.8091260	3.3972490
H	2.5577510	-2.8934220	2.7999110
H	1.9462050	-2.4842420	4.4000450
C	3.0260780	-0.0700770	1.8328710
H	3.1632390	0.9981230	1.6270400
C	4.2335240	-0.5309450	2.6652490

H	4.2916610	0.0626270	3.5793680
H	4.1230810	-1.5781370	2.9630420
C	5.5184510	-0.3743360	1.8494600
H	5.6939280	0.6919450	1.6738610
H	6.3656550	-0.7465090	2.4325740
C	5.4315660	-1.1044890	0.5087200
H	5.3450300	-2.1851110	0.6843590
H	6.3509340	-0.9520290	-0.0634130
C	4.2252530	-0.6287510	-0.3016970
H	4.1580070	-1.1734210	-1.2476780
H	4.3493650	0.4322570	-0.5485710
C	2.9240560	-0.8017720	0.4852700
H	2.7439060	-1.8719280	0.6506120
H	2.0824780	-0.4142400	-0.0975340
O	1.6927430	0.2686070	4.2552970
H	-0.9432440	8.0267180	4.9908320
H	6.3214300	3.4708170	1.4949920
H	-1.0023110	3.7458350	5.1113900
H	2.0290460	5.7808430	2.8801690
H	3.6506340	2.7535830	4.7670660
H	2.0864180	3.6889070	0.8959330
H	1.5259650	1.2465060	4.4396400
H	1.1696880	2.5031790	4.5723200

139

[4-H][4-OH]-IP

P	1.6172496	-2.9890335	-1.7582525
B	-1.4766004	-3.4281035	-2.3953605
C	0.9716736	-4.4114005	-2.6459395
C	1.8593646	-5.3819785	-3.1160565
H	2.9316176	-5.2514245	-2.9995765
C	1.3729006	-6.5303405	-3.7169855
H	2.0572276	-7.2848305	-4.0857115
C	-0.0025874	-6.7107485	-3.8250895
H	-0.3940374	-7.6146575	-4.2780705
C	-0.8775564	-5.7424535	-3.3541175
H	-1.9469194	-5.9037015	-3.4472505
C	-0.4163784	-4.5574675	-2.7659565
C	-1.6993014	-2.3427965	-3.5089715
C	-1.9442144	-0.9995245	-3.1935665
C	-2.0290574	-0.0325015	-4.1863275
H	-2.2010384	0.9993345	-3.9051125
C	-1.9000134	-0.3893095	-5.5233595
C	-1.6798174	-1.7189305	-5.8636045
H	-1.5824174	-2.0063575	-6.9048275
C	-1.5661274	-2.6771515	-4.8650215
C	-2.3658274	-3.5947105	-1.1269615
C	-3.4800704	-2.7809175	-0.8798695
C	-4.2100954	-2.8984195	0.2955525
H	-5.0523094	-2.2400775	0.4726785
C	-3.8498584	-3.8449225	1.2456815
C	-2.7622634	-4.6820395	1.0150505
H	-2.4825034	-5.4255485	1.7533975
C	-2.0325494	-4.5531765	-0.1558675
C	2.6188356	-3.5663575	-0.3533485
H	3.5834636	-3.8604115	-0.7955615
C	1.9814416	-4.7904495	0.3307365
H	1.0132746	-4.4870355	0.7471265
H	1.7942846	-5.5917925	-0.3874965
C	2.8898216	-5.2975065	1.4530755
H	2.4177206	-6.1563515	1.9383005

H	3.8287986	-5.6606905	1.0144085
C	3.1872546	-4.2046855	2.4786785
H	2.2601826	-3.9356355	2.9940585
H	3.8828006	-4.5774165	3.2360475
C	3.7510836	-2.9505315	1.8132665
H	4.7522846	-3.1587795	1.4123565
H	3.8582866	-2.1511045	2.5504765
C	2.8415026	-2.4539835	0.6887115
H	3.2619906	-1.5515195	0.2449845
H	1.8727606	-2.1747955	1.1077795
C	2.5762706	-1.9628195	-2.9254755
H	3.0747026	-2.7119225	-3.5588575
C	1.6009136	-1.1598605	-3.8075565
H	0.8777586	-1.8249715	-4.2842515
H	1.0217926	-0.4752225	-3.1787515
C	2.3550156	-0.3585755	-4.8670495
H	2.8348296	-1.0487615	-5.5729755
H	1.6346276	0.2303705	-5.4402805
C	3.4135456	0.5455305	-4.2375235
H	2.9196256	1.3014735	-3.6160975
H	3.9624056	1.0851515	-5.0141095
C	4.3848476	-0.2649275	-3.3787255
H	5.1145986	0.3939195	-2.9006915
H	4.9504426	-0.9516835	-4.0219575
C	3.6574336	-1.0695345	-2.2977905
H	3.2020896	-0.3760835	-1.5856535
H	4.3788346	-1.6754145	-1.7421005
H	0.1187006	-0.0836095	-0.8313885
O	0.3081976	-2.2824345	-1.2622495
H	0.2562516	-1.2835175	-0.9746005
H	-4.4146304	-3.9310005	2.1673935
H	-1.9732274	0.3662495	-6.2980745
H	-1.1773694	-5.2016295	-0.3201625
H	-3.7766104	-2.0331845	-1.6059595
H	-2.0317454	-0.6856015	-2.1589945
H	-1.3589194	-3.7043575	-5.1487395
B	-0.2328074	0.5625375	0.2138965
C	-1.7819444	0.9509525	-0.1090305
C	-2.8927834	0.4851785	0.6017485
C	-2.0539174	1.7968045	-1.1953325
C	-4.1953694	0.8135875	0.2347825
H	-2.7413494	-0.1378775	1.4759565
C	-3.3470344	2.1413065	-1.5669905
H	-1.2178144	2.2014105	-1.7617315
C	-4.4321954	1.6393815	-0.8558915
H	-5.0297654	0.4348885	0.8175565
H	-3.5121554	2.8064695	-2.4095675
H	-5.4453634	1.9039955	-1.1385205
C	0.7696666	1.8464605	0.1403165
C	0.7387346	2.9424285	1.0353115
C	1.7522866	1.8813985	-0.8570735
C	1.7035586	3.9498535	0.9340905
C	2.7011466	2.8898615	-0.9630415
H	1.7609866	1.0800685	-1.5885395
C	2.6879236	3.9291805	-0.0457715
H	1.6963306	4.7779835	1.6340965
H	3.4451966	2.8622055	-1.7542865
H	3.4236416	4.7247395	-0.0961565
C	0.0208166	-0.4008015	1.4995435
C	-0.6724904	-1.6103355	1.6633225
C	0.9759356	-0.1043315	2.4805835

C	-0.4534884	-2.4518215	2.7491715
H	-1.4050894	-1.9126165	0.9214835
C	1.2038846	-0.9325955	3.5744765
H	1.5614636	0.8041125	2.3872785
C	0.4811456	-2.1106835	3.7203165
H	-1.0242484	-3.3706405	2.8372855
H	1.9431246	-0.6521825	4.3188465
H	0.6434926	-2.7539505	4.5791785
P	-0.6438264	3.0360965	2.2752055
C	0.1235006	4.0433125	3.6454645
H	0.5038286	4.9933875	3.2540085
C	1.2922906	3.2571635	4.2818775
H	1.0734666	2.1848995	4.2447925
H	2.1946916	3.4107285	3.6826475
C	1.5527036	3.6685895	5.7440655
H	0.9494376	3.0490555	6.4177405
H	2.5977966	3.4785015	6.0045475
C	1.1930866	5.1352455	5.9726515
H	1.4867886	5.4584295	6.9753445
H	1.7648166	5.7521435	5.2695825
C	-0.3164704	5.3589675	5.7607905
H	-0.4900034	6.3895455	5.4341275
H	-0.8467914	5.2412815	6.7114255
C	-0.9097814	4.3824195	4.7267625
H	-1.8113844	4.8187715	4.2914345
H	-1.2258794	3.4528465	5.2133775
C	-1.7498564	4.2599885	1.3709275
H	-1.6955004	3.9002125	0.3369685
C	-3.2156224	4.1108755	1.8059255
H	-3.5083114	3.0606585	1.7393035
H	-3.3294334	4.4084165	2.8564655
C	-4.1408454	4.9699455	0.9413975
H	-4.1168084	4.5846545	-0.0851125
H	-5.1747214	4.8750545	1.2893855
C	-3.7095354	6.4369325	0.9427445
H	-3.8194904	6.8452105	1.9564715
H	-4.3624234	7.0284425	0.2926575
C	-2.2517314	6.5800995	0.5031715
H	-1.9436874	7.6304195	0.5390255
H	-2.1576564	6.2571305	-0.5416105
C	-1.3195204	5.7297785	1.3717625
H	-1.3374874	6.1240975	2.3962035
H	-0.2918234	5.8243785	1.0096825

69

[4-H]-

B	1.4186570	-4.0783270	-0.3972200
H	1.1486620	-2.8792710	-0.2920660
C	2.9697110	-4.1401880	-0.8947640
C	3.3714180	-4.6222910	-2.1474790
C	3.9954650	-3.6196720	-0.0895530
C	4.6990240	-4.6102640	-2.5652060
H	2.6193580	-5.0198310	-2.8231090
C	5.3272970	-3.6038770	-0.4877570
H	3.7340340	-3.2111330	0.8837900
C	5.6911900	-4.1052040	-1.7336760
H	4.9588620	-4.9992900	-3.5466000
H	6.0859500	-3.1951860	0.1750180
H	6.7297330	-4.0972630	-2.0515040
C	1.1355150	-4.7098460	1.0877160
C	2.0643130	-5.4110370	1.8660930

C	-0.1398420	-4.5505800	1.6554160
C	1.7511890	-5.9125660	3.1276860
H	3.0581830	-5.5840470	1.4642250
C	-0.4731240	-5.0556450	2.9056900
H	-0.8948860	-4.0164730	1.0830250
C	0.4782610	-5.7414050	3.6568730
H	2.5041620	-6.4536880	3.6950090
H	-1.4758970	-4.9130170	3.3000590
H	0.2289190	-6.1377040	4.6367430
C	0.3576280	-4.6899460	-1.4899480
C	-0.5070920	-3.7917570	-2.1374910
C	0.2214420	-6.0541460	-1.8459330
C	-1.4492210	-4.1813020	-3.0794400
H	-0.4179600	-2.7399040	-1.8831100
C	-0.7291150	-6.4424690	-2.7987630
C	-1.5636470	-5.5224660	-3.4173520
H	-2.0886430	-3.4401220	-3.5513600
H	-0.8241790	-7.4856770	-3.0805110
H	-2.2903220	-5.8532190	-4.1534500
P	1.3357000	-7.3063200	-1.0408620
C	0.1586400	-8.0376070	0.2267850
C	1.4562040	-8.6140420	-2.3822990
H	-0.2064280	-7.1314990	0.7271970
C	0.9028260	-8.8520960	1.2965230
C	-1.0580960	-8.8077920	-0.2919890
H	0.4625680	-8.9395700	-2.7159230
C	2.2005610	-8.0125830	-3.5856440
C	2.1946850	-9.8603260	-1.8738560
H	1.3197250	-9.7669590	0.8575550
H	1.7427610	-8.2675460	1.6773600
C	-0.0328080	-9.2386920	2.4437540
C	-1.9925670	-9.2035780	0.8561000
H	-1.6052920	-8.2018500	-1.0184330
H	-0.7273560	-9.7177240	-0.8128560
H	1.6448700	-7.1568990	-3.9776660
H	3.1671920	-7.6222240	-3.2414030
C	2.4313340	-9.0388660	-4.6960690
C	2.4325070	-10.8845170	-2.9874390
H	3.1573420	-9.5573300	-1.4418680
H	1.6249570	-10.3327630	-1.0699470
H	0.5070310	-9.8385230	3.1854910
H	-0.3580740	-8.3221730	2.9495440
C	-1.2552280	-10.0029710	1.9319070
H	-2.4006150	-8.2898570	1.3063560
H	-2.8437070	-9.7772610	0.4711540
H	1.4617160	-9.3526040	-5.1062280
H	2.9880710	-8.5790910	-5.5196310
C	3.1744960	-10.2681670	-4.1729230
H	2.9884990	-11.7423340	-2.5930800
H	1.4632000	-11.2685960	-3.3334070
H	-0.9285550	-10.9614330	1.5041720
H	-1.9324670	-10.2414290	2.7598590
H	4.1793350	-9.9669240	-3.8492820
H	3.3053500	-11.0088030	-4.9698360

70

[4-OH]-

P	3.2543600	1.2941240	3.1547830
B	0.5779680	2.8277150	4.4667040
C	1.6612120	1.6238070	2.2412940
C	1.5242680	1.2781360	0.8896370

H	2.3566180	0.8511570	0.3442500
C	0.3396010	1.4755990	0.1938050
H	0.2724030	1.1945330	-0.8530260
C	-0.7451760	2.0313450	0.8541960
H	-1.6872220	2.1879600	0.3364620
C	-0.6119620	2.3885530	2.1882160
H	-1.4680760	2.8234840	2.6967720
C	0.5712810	2.2186020	2.9251190
C	0.8537270	4.4498790	4.4137190
C	1.4555060	5.0669280	5.5204070
C	1.7373240	6.4281660	5.5489540
H	2.2125920	6.8652410	6.4232360
C	1.4241810	7.2303940	4.4561460
C	0.8222570	6.6488110	3.3468570
H	0.5769900	7.2583960	2.4813640
C	0.5446270	5.2842070	3.3333680
C	-0.8685610	2.4930910	5.1876490
C	-1.6399110	3.4383670	5.8722280
C	-2.8146030	3.0961180	6.5386470
H	-3.3852110	3.8624550	7.0567490
C	-3.2594120	1.7801710	6.5437050
C	-2.5171140	0.8154210	5.8689270
H	-2.8541290	-0.2177320	5.8558020
C	-1.3512090	1.1752810	5.2043390
C	4.3576280	0.5688920	1.8148940
H	4.2569600	1.1254760	0.8737160
C	3.9678360	-0.9011790	1.5802510
H	4.0675090	-1.4361190	2.5339500
H	2.9166400	-0.9853410	1.2943410
C	4.8546090	-1.5747320	0.5311150
H	4.5551340	-2.6201780	0.3981330
H	4.7093200	-1.0785040	-0.4378880
C	6.3301010	-1.4919640	0.9235570
H	6.4883300	-2.0821560	1.8359750
H	6.9626890	-1.9345620	0.1459710
C	6.7438430	-0.0444140	1.1894250
H	6.6903560	0.5213510	0.2496610
H	7.7869680	-0.0030460	1.5222850
C	5.8372380	0.6298460	2.2238990
H	6.1554820	1.6656270	2.3540100
H	5.9631240	0.1392220	3.1986170
C	3.8378780	3.0642770	3.3037080
H	2.9388170	3.5395360	3.6980230
C	4.9059940	3.2669260	4.3846740
H	4.5545190	2.7984030	5.3079330
H	5.8483720	2.7769590	4.1075330
C	5.1643140	4.7588570	4.6177190
H	4.2593940	5.2083710	5.0410900
H	5.9672820	4.8929760	5.3522330
C	5.5097690	5.4842170	3.3155970
H	6.4624520	5.0972360	2.9247430
H	5.6559070	6.5532800	3.5065460
C	4.4157370	5.2796630	2.2656470
H	4.6814110	5.7933600	1.3341300
H	3.4825000	5.7279690	2.6278850
C	4.1768610	3.7900690	1.9999850
H	5.0749920	3.3613490	1.5338910
H	3.3549430	3.6637830	1.2880990
O	1.6710690	2.2761230	5.3163330
H	1.5210610	1.3496620	5.4949360
H	-4.1745910	1.5087600	7.0616770

H	1.6502060	8.2924630	4.4679470
H	-0.7993340	0.4097910	4.6619130
H	-1.3113880	4.4738040	5.8842350
H	0.0942630	4.8537080	2.4439040
H	1.7279210	4.4384690	6.3623020

70

[4-OH]+

P	1.8478920	0.5153690	2.1128270
B	-0.1125890	2.9602370	3.2611490
C	0.0626080	0.6550710	1.9091470
C	-0.5604000	-0.3586300	1.1746360
H	0.0126280	-1.1860370	0.7698810
C	-1.9307760	-0.3309100	0.9651560
H	-2.4057260	-1.1150200	0.3885080
C	-2.6849970	0.6934840	1.5192780
H	-3.7587820	0.7160790	1.3758600
C	-2.0683700	1.6805350	2.2773200
H	-2.6768310	2.4578230	2.7269740
C	-0.6821720	1.7063230	2.4779270
C	0.9396740	3.8745200	2.5348470
C	1.9707350	4.5236410	3.2396830
C	2.9294200	5.2848450	2.5795240
H	3.7215870	5.7645800	3.1422750
C	2.8554800	5.4482290	1.2011240
C	1.8306950	4.8389030	0.4837720
H	1.7615420	4.9813190	-0.5887370
C	0.8996440	4.0468260	1.1412530
C	-0.6416040	3.2698380	4.6795100
C	-0.6255990	4.5734490	5.2072930
C	-1.1119030	4.8399510	6.4781650
H	-1.1073480	5.8536460	6.8609750
C	-1.6055120	3.8021800	7.2614780
C	-1.6302030	2.5020010	6.7670940
H	-2.0182530	1.6964510	7.3792790
C	-1.1681320	2.2446940	5.4860810
C	2.2848120	-1.1827890	2.5699780
H	1.9950480	-1.8019640	1.7110910
C	1.4793780	-1.6319370	3.8060520
H	1.7099790	-0.9632030	4.6426110
H	0.4066120	-1.5525130	3.6131570
C	1.8419720	-3.0694200	4.1850090
H	1.2851330	-3.3546770	5.0805580
H	1.5195860	-3.7465720	3.3845010
C	3.3441770	-3.2285210	4.4146910
H	3.6439640	-2.6301380	5.2833440
H	3.5806610	-4.2682020	4.6519600
C	4.1386540	-2.7762720	3.1906280
H	3.9198270	-3.4406430	2.3456640
H	5.2117990	-2.8525150	3.3805070
C	3.8009780	-1.3350330	2.7991250
H	4.3598760	-1.0576020	1.9018920
H	4.1108270	-0.6542190	3.5986920
C	2.6654390	1.1061600	0.5912190
H	1.9761670	1.8707010	0.2161820
C	4.0202880	1.7874110	0.8505620
H	3.8995530	2.6203600	1.5486970
H	4.7210580	1.0809820	1.3070500
C	4.6007510	2.3123120	-0.4644930
H	3.9555280	3.1156900	-0.8399170
H	5.5796380	2.7579130	-0.2724450

C	4.7088930	1.2105040	-1.5178080
H	5.4265280	0.4524420	-1.1806380
H	5.1021850	1.6202390	-2.4508250
C	3.3533400	0.5501920	-1.7662930
H	3.4470450	-0.2578670	-2.4954410
H	2.6603000	1.2841390	-2.1945960
C	2.7571870	-0.0063350	-0.4706750
H	3.3924470	-0.8205880	-0.1051570
H	1.7678250	-0.4285710	-0.6719890
O	2.3398860	1.3574980	3.3780710
H	2.3838660	2.3188500	3.2544570
H	-1.9779230	4.0081350	8.2585160
H	3.5905210	6.0564140	0.6868400
H	-1.2050440	1.2298270	5.1030660
H	-0.2493740	5.3921910	4.6032440
H	2.0207900	4.4288160	4.3206430
H	0.1114150	3.5664180	0.5689890

69

4-O

P	0.0492486	-0.7537599	-0.2867758
B	-1.1043184	1.3125671	0.8072362
C	-1.6803024	-0.6588349	-0.7222288
C	-2.4458584	-1.4828079	-1.5441908
H	-2.0166584	-2.3560239	-2.0261598
C	-3.7841184	-1.1696149	-1.7232858
H	-4.4107214	-1.7932899	-2.3502768
C	-4.3242814	-0.0518299	-1.0846418
H	-5.3746164	0.1818101	-1.2233728
C	-3.5417494	0.7567651	-0.2711818
H	-3.9845404	1.6140871	0.2260552
C	-2.1898274	0.4705611	-0.0702258
C	-0.4881794	2.5755551	-0.0025798
C	0.5135006	3.3494641	0.5999062
C	1.1785536	4.3535071	-0.0909008
H	1.9545796	4.9297921	0.4025582
C	0.8484386	4.6250561	-1.4159488
C	-0.1576264	3.8918471	-2.0301368
H	-0.4352844	4.1052881	-3.0575408
C	-0.8133074	2.8832361	-1.3268748
C	-1.5986304	1.6348951	2.2980312
C	-2.3596494	2.7806671	2.5575442
C	-2.8668764	3.0497461	3.8236492
H	-3.4537504	3.9467381	3.9932032
C	-2.6117214	2.1775341	4.8753072
C	-1.8466784	1.0401051	4.6461312
H	-1.6338104	0.3574761	5.4628862
C	-1.3534404	0.7761931	3.3733002
C	0.5310116	-2.4196409	0.2521862
H	0.4050806	-3.0616279	-0.6318658
C	-0.4258134	-2.9001969	1.3579612
H	-0.3765364	-2.1959859	2.1954882
H	-1.4575974	-2.8864809	0.9976892
C	-0.0450944	-4.2993259	1.8426112
H	-0.7184674	-4.6000689	2.6496922
H	-0.1892894	-5.0185769	1.0261042
C	1.4087036	-4.3511939	2.3122232
H	1.5270836	-3.7036819	3.1897472
H	1.6697826	-5.3647359	2.6290762
C	2.3579846	-3.8825979	1.2098722
H	2.3114216	-4.5862779	0.3687482

H	3.3914196	-3.8867909	1.5672422
C	1.9975706	-2.4815349	0.7106992
H	2.6665006	-2.1992699	-0.1062908
H	2.1477466	-1.7521269	1.5148182
C	1.0513626	-0.1610209	-1.6898238
H	0.3951486	0.6056471	-2.1217828
C	2.3418506	0.5547991	-1.2643908
H	2.1051136	1.3303151	-0.5337528
H	3.0295016	-0.1509359	-0.7841298
C	3.0150006	1.1676961	-2.4932638
H	2.3643306	1.9556411	-2.8915208
H	3.9492756	1.6523021	-2.1969218
C	3.2794296	0.1153411	-3.5721038
H	4.0043426	-0.6164289	-3.1917558
H	3.7344606	0.5791171	-4.4518168
C	1.9954216	-0.6145079	-3.9728818
H	2.2119326	-1.3916049	-4.7115498
H	1.3089546	0.0955181	-4.4496448
C	1.2990566	-1.2343149	-2.7579138
H	1.9317556	-2.0305549	-2.3458758
H	0.3553836	-1.6980379	-3.0659628
O	0.1051016	0.2462561	0.8972912
H	-2.9998474	2.3862671	5.8665072
H	1.3645206	5.4096551	-1.9589438
H	-0.7499724	-0.1108989	3.2084682
H	-2.5461994	3.4860021	1.7517802
H	0.7794736	3.1477951	1.6335392
H	-1.5952794	2.3193351	-1.8290428

68
4

C	-4.4677100	-5.6709250	-4.0650280
C	-5.0720570	-6.5565070	-4.9641520
C	-4.6681640	-7.8822470	-5.0439790
C	-3.6660490	-8.3529840	-4.2033750
C	-3.0665990	-7.4905710	-3.2961910
C	-3.4446570	-6.1469690	-3.2287990
B	-4.9397990	-4.1627680	-3.9827440
H	-5.8783840	-6.2074580	-5.6028880
H	-5.1464780	-8.5536000	-5.7488440
H	-3.3595830	-9.3922110	-4.2479530
H	-2.3054710	-7.8753090	-2.6266670
P	-2.7678170	-4.9151020	-2.0322510
C	-2.5498790	-1.2184550	-6.1663490
C	-2.7393270	-2.4926220	-6.6884520
C	-3.5071880	-3.4174170	-5.9935110
C	-4.1320450	-3.0858500	-4.7842140
C	-3.9413430	-1.7882710	-4.2876560
C	-3.1479920	-0.8688230	-4.9601290
H	-1.9334560	-0.4999500	-6.6955910
H	-2.2714340	-2.7702630	-7.6265710
H	-3.6127450	-4.4233750	-6.3879080
H	-4.3903640	-1.5120400	-3.3394370
H	-2.9938410	0.1210730	-4.5449200
C	-8.6405900	-3.2596020	-1.8311590
C	-7.9365490	-4.4281160	-1.5585980
C	-6.7696870	-4.7077970	-2.2539430
C	-6.2586700	-3.8223790	-3.2158590
C	-6.9921410	-2.6548070	-3.4746490
C	-8.1721790	-2.3769330	-2.7969740
H	-9.5580540	-3.0410440	-1.2953520

H	-8.3039790	-5.1220980	-0.8106610
H	-6.2344720	-5.6284120	-2.0471740
H	-6.6393100	-1.9607210	-4.2298240
H	-8.7267050	-1.4721730	-3.0201260
C	0.5846630	-3.7278940	-4.2482320
C	-0.8656040	-4.0468710	-3.8826840
C	-0.9694490	-4.7281830	-2.5104580
C	-0.0864330	-5.9798800	-2.4657310
C	1.3675050	-5.6464840	-2.8140770
C	1.4696810	-4.9727000	-4.1829280
H	0.9725440	-2.9724350	-3.5524940
H	0.6210150	-3.2830510	-5.2473980
H	-1.2930090	-4.7113830	-4.6436600
H	-1.4636570	-3.1325750	-3.8991640
H	-0.5880670	-4.0157830	-1.7646010
H	-0.1294780	-6.4535760	-1.4800730
H	-0.4622900	-6.7094110	-3.1919960
H	1.7771620	-4.9749390	-2.0482850
H	1.9739250	-6.5573490	-2.7910840
H	1.1484670	-5.6830520	-4.9556370
H	2.5100430	-4.7147640	-4.4034590
C	-4.0902720	-7.0525990	1.3187760
C	-4.1052980	-6.2873970	-0.0059500
C	-2.6868530	-5.9168090	-0.4626420
C	-1.9477780	-5.1402260	0.6361450
C	-1.9395020	-5.9072480	1.9608870
C	-3.3560190	-6.2695770	2.4075380
H	-3.5918730	-8.0193870	1.1709610
H	-5.1152760	-7.2721380	1.6327240
H	-4.6963240	-5.3694280	0.1088720
H	-4.5987530	-6.8871410	-0.7768410
H	-2.1376480	-6.8484920	-0.6488250
H	-0.9189840	-4.9289370	0.3283530
H	-2.4399500	-4.1694760	0.7808960
H	-1.3535810	-6.8275120	1.8389820
H	-1.4366990	-5.3138230	2.7305350
H	-3.9114170	-5.3480010	2.6238480
H	-3.3255770	-6.8458510	3.3371300

105
[5-H][4-OH]-TS
P 0.101215 4.632910 0.201660
B -2.860145 4.012538 -0.194561
C -0.437494 2.984886 -0.275483
C 0.452024 1.937437 -0.512721
H 1.521493 2.083984 -0.399639
C -0.035895 0.691731 -0.877301
H 0.648536 -0.127481 -1.063271
C -1.409092 0.498960 -0.983711
H -1.796940 -0.476404 -1.255745
C -2.288103 1.543871 -0.729590
H -3.357168 1.369659 -0.798397
C -1.824452 2.816709 -0.380744
C -2.996489 5.012422 -1.402648
C -3.364436 6.351505 -1.210144
C -3.396755 7.247747 -2.269668
H -3.666158 8.280627 -2.082505
C -3.082679 6.821320 -3.555727
C -2.729664 5.495162 -3.775250
H -2.486904 5.154487 -4.776076
C -2.677971 4.608043 -2.706408

C -3.887444 3.915633 0.979492
C -5.113208 4.592508 0.962149
C -6.013434 4.482296 2.014481
H -6.954947 5.018549 1.977863
C -5.703881 3.693748 3.114261
C -4.496654 3.002715 3.153415
H -4.253718 2.383023 4.009622
C -3.607506 3.110246 2.095675
C 1.467680 4.487988 1.393093
H 2.288095 3.987269 0.859336
C 1.032225 3.621775 2.589332
H 0.164182 4.090171 3.064724
H 0.721861 2.629691 2.250298
C 2.161864 3.500703 3.612962
H 1.808886 2.916116 4.466929
H 2.998703 2.944620 3.169210
C 2.645713 4.873458 4.077847
H 1.830595 5.375009 4.610007
H 3.473919 4.763855 4.783903
C 3.074353 5.738191 2.893433
H 3.957594 5.297368 2.412222
H 3.360883 6.736535 3.235087
C 1.948967 5.869064 1.866583
H 2.288154 6.472838 1.020099
H 1.111183 6.396496 2.333859
C 0.590392 5.510820 -1.324556
H -0.128251 5.119271 -2.055995
C 0.365181 7.029005 -1.249295
H -0.665073 7.238311 -0.954598
H 1.017652 7.475107 -0.491241
C 0.656124 7.667833 -2.607139
H -0.084930 7.307004 -3.330711
H 0.526253 8.750802 -2.533491
C 2.064834 7.329784 -3.096832
H 2.800811 7.775165 -2.415009
H 2.240776 7.772311 -4.081235
C 2.286279 5.817345 -3.156003
H 3.309287 5.591912 -3.470259
H 1.618177 5.382313 -3.909369
C 2.004814 5.156463 -1.803746
H 2.742580 5.506013 -1.071182
H 2.122638 4.070880 -1.889260
H -0.838298 7.393613 1.415453
O -1.145322 5.296422 0.832290
H -1.060362 6.505410 1.274508
H -6.402339 3.613348 3.940024
H -3.112599 7.521129 -4.383955
H -2.664856 2.573494 2.139433
H -5.369601 5.217378 0.114382
H -3.591293 6.711195 -0.212417
H -2.378131 3.580156 -2.889398
B -1.453148 7.906779 2.492748
C -2.948710 8.227692 1.968500
C -4.115806 7.685406 2.511614
C -3.109671 9.126348 0.901240
C -5.375396 8.003046 2.007294
H -4.051361 6.999652 3.349100
C -4.358344 9.453433 0.393396
H -2.226858 9.587330 0.466158
C -5.503292 8.882533 0.942449
H -6.258720 7.559100 2.454281

H -4.443819 10.159155 -0.427188
H -6.483218 9.132652 0.550259
C -0.538260 9.237268 2.588572
C -1.019018 10.336134 3.313755
C 0.738474 9.362579 2.035144
C -0.262716 11.487808 3.487410
H -2.013012 10.284750 3.748654
C 1.504818 10.513705 2.194361
H 1.152291 8.540511 1.459384
C 1.006893 11.582423 2.926611
H -0.666172 12.318061 4.057885
H 2.490789 10.575229 1.744562
H 1.598104 12.482498 3.055925
C -1.267960 6.901056 3.744977
C -1.907412 5.653873 3.804596
C -0.448395 7.233940 4.830648
C -1.739316 4.788953 4.878560
H -2.542794 5.339997 2.985299
C -0.276834 6.378304 5.915416
H 0.068053 8.188350 4.831058
C -0.919182 5.147370 5.942917
H -2.254393 3.833679 4.881877
H 0.363285 6.675347 6.740063
H -0.787946 4.477576 6.786241

34
5

B	0.7891100	-0.0735390	-0.0006250
C	0.0062410	1.2836400	0.0008930
C	0.4213100	2.3648030	0.7945490
C	-1.1383740	1.4662280	-0.7913510
C	-0.2790780	3.5634000	0.8095010
H	1.3020190	2.2575450	1.4192870
C	-1.8261160	2.6721710	-0.8038100
H	-1.4867330	0.6501310	-1.4159990
C	-1.4007260	3.7209470	0.0034630
H	0.0524670	4.3779670	1.4439160
H	-2.6980600	2.7933630	-1.4370300
H	-1.9430240	4.6602470	0.0044980
C	0.0165550	-1.4228320	-0.1930210
C	0.6017960	-2.5098560	-0.8616360
C	-1.2885030	-1.5924680	0.2959650
C	-0.0856020	-3.7017590	-1.0468500
H	1.6095000	-2.4124990	-1.2522560
C	-1.9702070	-2.7918990	0.1407040
H	-1.7707410	-0.7722980	0.8176770
C	-1.3713120	-3.8466830	-0.5384890
H	0.3827140	-4.5210840	-1.5810120
H	-2.9713160	-2.9032000	0.5423500
H	-1.9061400	-4.7807990	-0.6716950
C	2.3440530	-0.0810590	0.1910340
C	3.1441000	0.9639450	-0.2979150
C	2.9922590	-1.1323420	0.8586740
C	4.5237410	0.9535690	-0.1432300
H	2.6751230	1.7923760	-0.8187110
C	4.3682340	-1.1339390	1.0434970
H	2.4037040	-1.9563040	1.2487940
C	5.1371710	-0.0931870	0.5354020
H	5.1211440	1.7646860	-0.5446160
H	4.8433000	-1.9497720	1.5770360
H	6.2135870	-0.0978150	0.6683100

105
endo-4-H_2O-5-TS
P 1.634404 4.683728 -0.130610
B 0.423251 3.429882 2.258599
C -0.020720 3.803502 -0.216443
C -0.826001 3.790877 -1.359727
H -0.525891 4.295830 -2.271885
C -2.044100 3.126989 -1.348689
H -2.662723 3.120772 -2.239132
C -2.462473 2.462041 -0.199733
H -3.405756 1.926682 -0.197404
C -1.676305 2.496664 0.942143
H -2.009931 1.993604 1.844742
C -0.460693 3.182461 0.954842
C -0.044351 4.719586 3.105239
C 0.764929 5.241787 4.121971
C 0.450806 6.427194 4.775207
H 1.111174 6.805554 5.547995
C -0.699322 7.127062 4.431595
C -1.528016 6.626560 3.434837
H -2.431376 7.161748 3.160821
C -1.199405 5.442665 2.783207
C 0.977626 2.138949 3.028368
C 0.897638 1.967921 4.412475
C 1.427485 0.843154 5.039657
H 1.350236 0.738624 6.116800
C 2.055808 -0.142057 4.290602
C 2.132656 -0.005723 2.907560
H 2.605938 -0.779030 2.311183
C 1.592402 1.114858 2.293055
C 2.268697 4.038692 -1.765956
H 1.430239 4.266907 -2.434286
C 2.398573 2.504716 -1.706941
H 3.169363 2.236364 -0.984312
H 1.460009 2.059931 -1.369306
C 2.790453 1.933346 -3.069175
H 2.913420 0.850128 -2.980702
H 1.976742 2.103517 -3.786624
C 4.073201 2.578954 -3.588594
H 4.895310 2.349208 -2.899954
H 4.342238 2.163640 -4.564248
C 3.905260 4.093405 -3.686594
H 3.134437 4.323836 -4.434020
H 4.830647 4.561831 -4.033263
C 3.513029 4.708529 -2.339331
H 3.337210 5.776137 -2.477075
H 4.347209 4.604448 -1.646677
C 0.995627 6.424299 -0.051709
H -0.050410 6.263726 0.229919
C 1.635469 7.273852 1.046727
H 1.566462 6.759752 2.007450
H 2.693763 7.428107 0.826195
C 0.935281 8.629845 1.141669
H -0.098342 8.475483 1.473621
H 1.423797 9.235180 1.910367
C 0.948164 9.356982 -0.202827
H 1.985736 9.586356 -0.478027
H 0.422189 10.312810 -0.125373
C 0.315768 8.497869 -1.298874
H 0.360659 9.011347 -2.263691

H -0.745668 8.340906 -1.070745
C 1.007896 7.136404 -1.411458
H 2.038657 7.291926 -1.744585
H 0.510881 6.528869 -2.174065
H 3.193897 5.129068 0.275361
O 1.783501 3.991459 1.423109
H 2.520224 4.351000 1.953416
H 2.474833 -1.016038 4.777364
H -0.948808 8.053662 4.937160
H 1.648750 1.202724 1.210844
H 0.412474 2.727756 5.016187
H 1.674909 4.720754 4.400921
H -1.855321 5.077892 1.998735
B 4.668879 5.061897 1.063522
C 4.310166 5.166332 2.637541
C 4.086405 6.430172 3.215715
C 4.393335 4.084311 3.530323
C 3.956337 6.605749 4.583423
H 4.050730 7.303381 2.575283
C 4.254307 4.248106 4.905461
H 4.602392 3.089285 3.161476
C 4.040493 5.510740 5.437594
H 3.796305 7.599763 4.986192
H 4.323533 3.383486 5.555650
H 3.942051 5.643534 6.509533
C 5.228812 3.666176 0.509387
C 6.360237 3.643617 -0.319040
C 4.671346 2.420961 0.835083
C 6.906048 2.454745 -0.789181
H 6.825066 4.580886 -0.605782
C 5.209595 1.226474 0.375223
H 3.771466 2.365319 1.436165
C 6.333223 1.237709 -0.442041
H 7.781861 2.480339 -1.428674
H 4.741346 0.287800 0.652336
H 6.757398 0.307990 -0.804983
C 5.384655 6.386092 0.512971
C 4.927681 7.240564 -0.489491
C 6.615353 6.714350 1.104981
C 5.645945 8.363324 -0.890313
H 3.980508 7.033926 -0.968169
C 7.347948 7.823589 0.706476
H 7.005676 6.084808 1.899529
C 6.863734 8.658082 -0.295602
H 5.250177 9.005995 -1.669868
H 8.296818 8.042389 1.184431
H 7.429621 9.529902 -0.604452

105
exo-4-H_2O-5-TS
P 1.546129 0.285703 1.659714
B 0.691353 2.654290 3.274601
C -0.166811 0.847451 1.578015
C -1.153238 0.251436 0.791650
H -0.920232 -0.599528 0.170032
C -2.432135 0.781741 0.788555
H -3.193325 0.325211 0.167487
C -2.724383 1.891730 1.574150
H -3.727475 2.303838 1.576084
C -1.739396 2.470694 2.361150
H -1.982042 3.324635 2.985585

C -0.435489 1.970736 2.377399
C 1.501066 3.870536 2.630044
C 2.739909 4.268474 3.155841
C 3.512979 5.248700 2.546735
H 4.471904 5.525869 2.970738
C 3.051130 5.878480 1.396112
C 1.815593 5.523545 0.870193
H 1.445356 6.014244 -0.023230
C 1.057781 4.528747 1.478952
C 0.441395 2.621875 4.843902
C 0.721878 3.711083 5.675644
C 0.464087 3.667652 7.042464
H 0.686586 4.529442 7.662207
C -0.079670 2.524491 7.611382
C -0.382694 1.432195 6.804236
H -0.823518 0.541376 7.238320
C -0.134133 1.490894 5.441117
C 1.918963 -1.348676 2.363525
H 1.723911 -2.061983 1.562127
C 0.988604 -1.678084 3.538549
H 1.128090 -0.940162 4.336589
H -0.054753 -1.628515 3.214219
C 1.301359 -3.080307 4.067017
H 0.658290 -3.292732 4.925704
H 1.054217 -3.812961 3.292507
C 2.772692 -3.224823 4.453446
H 2.999430 -2.563371 5.299869
H 2.973100 -4.246594 4.786685
C 3.683613 -2.870269 3.278804
H 3.516953 -3.584126 2.462957
H 4.735560 -2.947859 3.567280
C 3.399818 -1.456033 2.765108
H 4.048084 -1.237582 1.912657
H 3.629060 -0.733847 3.559314
C 2.701695 0.864194 0.361323
H 3.022414 -0.060372 -0.127172
C 1.985393 1.736594 -0.677848
H 1.141269 1.185079 -1.091158
H 1.593570 2.635963 -0.192888
C 2.946720 2.131351 -1.799506
H 3.230425 1.231069 -2.354047
H 2.425913 2.790281 -2.499810
C 4.202722 2.811722 -1.257234
H 3.930863 3.763161 -0.781676
H 4.888088 3.044547 -2.076782
C 4.899261 1.921492 -0.229455
H 5.783786 2.420475 0.176478
H 5.234680 0.994978 -0.709980
C 3.947047 1.575784 0.919497
H 3.658252 2.513517 1.409182
H 4.471454 0.948197 1.648589
O 1.902980 1.267370 2.988353
H 2.815425 1.575307 3.024389
H -0.278400 2.486650 8.676818
H 3.648833 6.645577 0.916740
H -0.404996 0.642956 4.819830
H 1.136488 4.618232 5.249056
H 3.111214 3.799545 4.064550
H 0.104471 4.249690 1.040786
B 1.031685 -2.063367 -0.935996
H 0.959660 -1.054846 -0.149847

C 2.528790 -1.944997 -1.563563
C 2.756416 -1.449185 -2.853976
C 3.679765 -2.221964 -0.805545
C 4.037891 -1.219844 -3.347257
H 1.903677 -1.231176 -3.489136
C 4.966460 -1.993564 -1.280690
H 3.567690 -2.633751 0.193152
C 5.152571 -1.480745 -2.559828
H 4.165618 -0.834073 -4.353871
H 5.824398 -2.224389 -0.656291
H 6.151940 -1.302709 -2.942043
C 0.749457 -3.388764 -0.025358
C 1.581575 -4.514288 0.012897
C -0.432545 -3.477116 0.729619
C 1.271227 -5.645070 0.767050
H 2.491272 -4.521687 -0.578461
C -0.759789 -4.597226 1.480631
H -1.134302 -2.648078 0.712139
C 0.099384 -5.692169 1.508595
H 1.943771 -6.497077 0.761387
H -1.687878 -4.620734 2.043035
H -0.148911 -6.571713 2.092761
C -0.138121 -1.897271 -2.052951
C -0.651880 -0.662327 -2.466330
C -0.655418 -3.026480 -2.704356
C -1.619533 -0.548525 -3.460455
H -0.292119 0.248249 -1.997115
C -1.615919 -2.930872 -3.703559
H -0.295792 -4.009686 -2.415195
C -2.106892 -1.687557 -4.086897
H -1.990734 0.431060 -3.745522
H -1.987244 -3.830920 -4.183323
H -2.860897 -1.608971 -4.862830

71
[4-H][4-OH]-TS
P 1.965096 0.500778 2.393278
B 0.368975 2.662441 3.227526
C 0.203421 0.501255 1.875007
C -0.429746 -0.464266 1.092364
H 0.100242 -1.343441 0.739009
C -1.769368 -0.309775 0.762501
H -2.267854 -1.058260 0.157037
C -2.471462 0.801056 1.221556
H -3.521227 0.913446 0.972213
C -1.838806 1.754871 2.008355
H -2.399057 2.606002 2.383301
C -0.491557 1.624232 2.344299
C 1.053584 3.842583 2.361785
C 2.167134 4.529087 2.864554
C 2.843343 5.481367 2.112621
H 3.707986 5.987892 2.528741
C 2.409665 5.788003 0.826683
C 1.293724 5.140882 0.312146
H 0.940540 5.379015 -0.685908
C 0.630689 4.181590 1.072925
C -0.281730 3.072278 4.630893
C -0.583580 4.397136 4.955535
C -1.192674 4.730961 6.162417
H -1.417305 5.768611 6.386560
C -1.507247 3.738788 7.080561

C -1.215466 2.411110 6.781251
H -1.459865 1.628930 7.492712
C -0.617588 2.089382 5.571201
C 2.262916 -1.269064 2.784790
H 1.779904 -1.801556 1.955320
C 1.518711 -1.622543 4.082627
H 1.951188 -1.040252 4.905456
H 0.465778 -1.337579 4.006466
C 1.644428 -3.116774 4.386394
H 1.148179 -3.339672 5.334884
H 1.114402 -3.685108 3.611700
C 3.106617 -3.562442 4.431276
H 3.605907 -3.073061 5.276734
H 3.167736 -4.639830 4.608829
C 3.838248 -3.195513 3.139327
H 3.410096 -3.764516 2.304169
H 4.892183 -3.479428 3.205323
C 3.729653 -1.696297 2.847519
H 4.243792 -1.452669 1.913316
H 4.231701 -1.130389 3.638251
C 2.793317 1.193622 0.903652
H 2.100763 2.001187 0.635914
C 4.170166 1.820718 1.124548
H 4.119237 2.550022 1.935594
H 4.886345 1.048885 1.429077
C 4.642359 2.504063 -0.160004
H 3.968283 3.341097 -0.380009
H 5.636410 2.932127 -0.003040
C 4.662145 1.530125 -1.338715
H 5.412822 0.751357 -1.150944
H 4.966253 2.046217 -2.253817
C 3.296339 0.872521 -1.537453
H 3.336468 0.143326 -2.351857
H 2.564878 1.635954 -1.828841
C 2.806250 0.187024 -0.258048
H 3.471842 -0.651277 -0.018862
H 1.807903 -0.224259 -0.426274
H 3.494742 0.842217 3.400152
O 1.617021 1.621071 3.567415
H 2.735103 1.538925 3.796027
H -1.977796 3.995272 8.023723
H 2.934072 6.530589 0.235093
H -0.402344 1.048325 5.345321
H -0.335399 5.185558 4.251335
H 2.514738 4.307120 3.870176
H -0.228005 3.672763 0.644376

34

[5-H]-

B	0.7891100	-0.0735390	-0.0006250
C	0.0062410	1.2836400	0.0008930
C	0.4213100	2.3648030	0.7945490
C	-1.1383740	1.4662280	-0.7913510
C	-0.2790780	3.5634000	0.8095010
H	1.3020190	2.2575450	1.4192870
C	-1.8261160	2.6721710	-0.8038100
H	-1.4867330	0.6501310	-1.4159990
C	-1.4007260	3.7209470	0.0034630
H	0.0524670	4.3779670	1.4439160
H	-2.6980600	2.7933630	-1.4370300
H	-1.9430240	4.6602470	0.0044980

C	0.0165550	-1.4228320	-0.1930210
C	0.6017960	-2.5098560	-0.8616360
C	-1.2885030	-1.5924680	0.2959650
C	-0.0856020	-3.7017590	-1.0468500
H	1.6095000	-2.4124990	-1.2522560
C	-1.9702070	-2.7918990	0.1407040
H	-1.7707410	-0.7722980	0.8176770
C	-1.3713120	-3.8466830	-0.5384890
H	0.3827140	-4.5210840	-1.5810120
H	-2.9713160	-2.9032000	0.5423500
H	-1.9061400	-4.7807990	-0.6716950
C	2.3440530	-0.0810590	0.1910340
C	3.1441000	0.9639450	-0.2979150
C	2.9922590	-1.1323420	0.8586740
C	4.5237410	0.9535690	-0.1432300
H	2.6751230	1.7923760	-0.8187110
C	4.3682340	-1.1339390	1.0434970
H	2.4037040	-1.9563040	1.2487940
C	5.1371710	-0.0931870	0.5354020
H	5.1211440	1.7646860	-0.5446160
H	4.8433000	-1.9497720	1.5770360
H	6.2135870	-0.0978150	0.6683100

69
[4-H]+
P 2.962117 2.481764 2.575825
B 0.095422 2.930784 4.039924
C 1.365630 2.511150 1.747803
C 1.357299 2.331884 0.361882
H 2.282573 2.185312 -0.186187
C 0.154485 2.328070 -0.327339
H 0.147063 2.200893 -1.402856
C -1.036499 2.469268 0.373823
H -1.981199 2.453027 -0.156767
C -1.023269 2.612536 1.755214
H -1.964008 2.694042 2.288940
C 0.173771 2.660199 2.480132
C 0.846094 4.179584 4.608615
C 1.413687 4.183167 5.894478
C 2.096516 5.290969 6.378855
H 2.533289 5.268311 7.370514
C 2.204904 6.436125 5.596529
C 1.642971 6.464214 4.324484
H 1.713619 7.361204 3.719671
C 0.987644 5.342822 3.833547
C -0.710487 1.951884 4.926517
C -1.252003 2.344846 6.164016
C -1.974315 1.457343 6.946812
H -2.398657 1.784160 7.888788
C -2.154020 0.145486 6.520525
C -1.628552 -0.272684 5.302461
H -1.775910 -1.293580 4.970055
C -0.930266 0.625841 4.510504
C 3.899467 0.998143 2.082404
H 4.123225 1.117488 1.014691
C 3.023490 -0.254418 2.277929
H 2.717109 -0.318861 3.330474
H 2.110320 -0.178510 1.682694
C 3.797409 -1.519457 1.899986
H 3.166963 -2.392817 2.081880
H 4.009299 -1.502581 0.824154

```
C 5.107945 -1.631312 2.677401
H 4.888656 -1.753922 3.745049
H 5.655520 -2.523232 2.365208
C 5.972996 -0.388192 2.476985
H 6.280333 -0.320743 1.426455
H 6.888815 -0.457250 3.068431
C 5.222836 0.890030 2.861738
H 5.856636 1.759561 2.673029
H 5.009056 0.874034 3.938113
C 3.862715 4.054387 2.348971
H 3.054725 4.788872 2.259142
C 4.692142 4.425130 3.589996
H 4.046994 4.451730 4.472612
H 5.466304 3.670691 3.768862
C 5.352360 5.789597 3.382962
H 4.569825 6.556031 3.331561
H 5.970295 6.028005 4.251838
C 6.188293 5.822086 2.103195
H 7.025270 5.119045 2.197092
H 6.626851 6.813022 1.966204
C 5.348801 5.449754 0.880679
H 5.965467 5.443196 -0.021027
H 4.570828 6.206817 0.726342
C 4.686298 4.079396 1.050975
H 5.462964 3.306924 1.084236
H 4.049822 3.864714 0.186599
H 2.721333 2.332070 3.948483
H -2.712150 -0.550896 7.135963
H 2.722600 7.307880 5.980089
H -0.542360 0.295006 3.551978
H -1.125052 3.367147 6.503216
H 0.556716 5.374928 2.836907
H 1.326198 3.299847 6.518682
```

Parahydrogen experiments

General. NMR experiments with parahydrogen were performed on a 400 MHz Bruker AV 400 NMR spectrometer equipped with a broad-band 5 mm RF probe. The standard temperature control unit of the NMR spectrometer was used for heating the samples. Parahydrogen-enriched H_2 gas (92%) referred to in the main text as simply parahydrogen was produced with a Bruker parahydrogen generator BPHG 90.

Experimental procedure. To perform experiments, parahydrogen was bubbled through 0.03 M solutions of the ansa-phosphinoborane **1** in degassed solvents (dry CD_2Cl_2 and moist (3 eq. H_2O) 1:1 CD_2Cl_2+CD_3CN) in a 5 mm sample tube inside the NMR magnet for ca. 5 s. Immediately after the parahydrogen flow was abruptly switched off the NMR measurement was taken. The bubbling procedure was performed under a 3.2 bar parahydrogen pressure in the same manner as explained in detail in Ref.[15] Parahydrogen was supplied to the bottom of the sample tube through a 1/32” PTFE tubing. The sample temperature was varied in experiments when it was required. Both π/4-pulses and π/2-pulses were used to probe the flip angle effects. We didn’t observe any specific influence of ^{1}H RF-pulse flip angle on the shape of the signals in the current study. The ^{1}H π /2-pulses provided

stronger antiphase signals than π/4-pulses. The opposite should be observed in the case of classical PASADENA effect for a weakly-coupled spin system. However, since J_{HH} is likely very small, we just cannot observe the normal effect, but do observe effects that stem from the heteronuclear two-spin orders (strong effect for the $^{31}P^{1}H$ pair and much weaker for the $^{11}B^{1}H$ pair of **1**-H_2 adduct) hyperpolarized via nuclear spin relaxation-driven transitions.

Since investigated ansa-phosphinoborane **1** is sensitive to moisture, the sample preparation procedures were done under inert Ar atmosphere to avoid any destructive influence on the results of the experiments.

References

1 Schlosser, M.; Heiss, C.; Marzi, E.; Scopelliti, R. Proton Mobility in 2-Substituted 1,3-Dichlorobenzenes: "Ortho" or "Meta" Metalation? European J. Org. Chem. 2006, 2006 (19), 4398–4404. https://doi.org/10.1002/ejoc.200600350.

2 Naumann, D.; Tyrra, W.; Pfolk, D. Die Ersten Nicht-fluorierten Arylxenon-Kationen: Reaktionen von Xenondifluorid Mit Chlorphenylbor-, -lithium-, -silicium- Und -zinn-Derivaten. ZAAC - J. Inorg. Gen. Chem. 1994, 620 (6), 987–992. https://doi.org/10.1002/zaac.19946200606.

3 Sheldrick, G. M. A Short History of SHELX. Acta Crystallogr. Sect. A Found. Crystallogr. 2008, 64 (1), 112–122. https://doi.org/10.1107/S0108767307043930.

4 Sheldrick, G. M. SHELXT - Integrated Space-Group and Crystal-Structure Determination. Acta Crystallogr. Sect. A Found. Crystallogr. 2015, 71 (1), 3–8. https://doi.org/10.1107/S2053273314026370.

5 Sheldrick, G. M. Crystal Structure Refinement with SHELXL. Acta Crystallogr. Sect. C Struct. Chem. 2015, 71 (1), 3–8. https://doi.org/10.1107/S2053229614024218.

6 Spek, A. L. Structure Validation in Chemical Crystallography. Acta Crystallogr. Sect. D Biol. Crystallogr. 2009, 65 (2), 148–155. https://doi.org/10.1107/S090744490804362X.

7 Spek, A. L. PLATON SQUEEZE: A Tool for the Calculation of the Disordered Solvent Contribution to the Calculated Structure Factors. Acta Crystallogr. Sect. C Struct. Chem. 2015, 71 (1), 9–18. https://doi.org/10.1107/S2053229614024929.

8 Hoops, S.; Sahle, S.; Gauges, R.; Lee, C.; Pahle, J.; Simus, N.; Singhal, M.; Xu, L.; Mendes, P.; Kummer, U. COPASI--a COmplex PAthway SImulator. Bioinformatics 2006, 22 (24), 3067–3074. https://doi.org/10.1093/bioinformatics/btl485.

9 Gaussian 09, Revision E.01, M. J. Frisch, G. W. Trucks, H. B. Schlegel, G. E. Scuseria, M. A. Robb, J. R. Cheeseman, G. Scalmani, V. Barone, B. Mennucci, G. A. Petersson, H. Nakatsuji, M. Caricato, X. Li, H. P. Hratchian, A. F. Izmaylov, J. Bloino, G. Zheng, J. L. Sonnenberg, M. Hada, M. Ehara, K. Toyota, R. Fukuda, J. Hasegawa, M. Ishida, T. Nakajima, Y. Honda, O. Kitao, H. Nakai, T. Vreven, J. A. Montgomery, Jr., J. E. Peralta, F. Ogliaro, M. Bearpark, J. J. Heyd, E. Brothers, K. N. Kudin, V. N. Staroverov, T. Keith, R. Kobayashi, J. Normand, K. Raghavachari, A. Rendell, J. C. Burant, S. S. Iyengar, J. Tomasi, M. Cossi, N. Rega, J. M. Millam, M. Klene, J. E. Knox, J. B. Cross, V. Bakken, C. Adamo, J. Jaramillo, R. Gomperts, R. E. Stratmann, O. Yazyev, A. J. Austin, R. Cammi, C. Pomelli, J. W. Ochterski, R. L. Martin, K. Morokuma, V. G. Zakrzewski, G. A. Voth, P. Salvador, J. J. Dannenberg, S. Dapprich, A. D. Daniels, O. Farkas, J. B. Foresman, J. V. Ortiz, J. Cioslowski, and D. J. Fox, Gaussian, Inc., Wallingford CT, 2013.

10 CYLview20; Legault, C. Y., Université de Sherbrooke, 2020 (http://www.cylview.org)

11 Chai, J.-D.; Head-Gordon, M. Long-Range Corrected Hybrid Density Functionals with Damped Atom–Atom Dispersion Corrections. Phys. Chem. Chem. Phys. 2008, 10 (44), 6615. https://doi.org/10.1039/b810189b.

12 Ditchfield, R.; Hehre, W. J.; Pople, J. A. Self-Consistent Molecular-Orbital Methods. IX. An Extended Gaussian-Type Basis for Molecular-Orbital Studies of Organic Molecules. J. Chem. Phys. 2003, 54 (2), 724. https://doi.org/10.1063/1.1674902.

13 Marenich, A. V.; Cramer, C. J.; Truhlar, D. G. Universal Solvation Model Based on Solute Electron Density and on a Continuum Model of the Solvent Defined by the Bulk Dielectric Constant and Atomic Surface Tensions. J. Phys. Chem. B 2009, 113 (18), 6378–6396. https://doi.org/10.1021/JP810292N.

14 Schrödinger Release 2019: MacroModel, Schrödinger, LLC, New York, NY, 2019.

15 Zhivonitko, V. V.; Sorochkina, K.; Chernichenko, K.; Kotai, B.; Foldes, T.; Papai, I.; Telkki, V.-V.; Repo, T.; Koptyug, I. Nuclear Spin Hyperpolarization with ansa-Aminoboranes: A Metal-Free Perspective for Parahydrogen-Induced Polarization. Phys. Chem. Chem. Phys. 2016, 18, 27784-27795.
